# Tunable Emission and Structural Insights of 6-Arylvinyl-2,4-bis(2′-hydroxyphenyl)pyrimidines and Their O^∧^N^∧^O-Chelated Boron Complexes

**DOI:** 10.1021/acsaom.4c00251

**Published:** 2024-09-20

**Authors:** Rodrigo Plaza-Pedroche, M. Paz Fernández-Liencres, Sonia B. Jiménez-Pulido, Nuria A. Illán-Cabeza, Sylvain Achelle, Amparo Navarro, Julián Rodríguez-López

**Affiliations:** † Universidad de Castilla-La Mancha, Área de Química Orgánica, Facultad de Ciencias y Tecnologías Químicas, Avda. Camilo José Cela 10, 13071 Ciudad Real, Spain; ‡ Departamento de Química Física y Analítica, Facultad de Ciencias Experimentales, Universidad de Jaén, Campus Las Lagunillas, 23071 Jaén, Spain; § Departamento de Química Inorgánica y Orgánica, Facultad de Ciencias Experimentales, Universidad de Jaén, Campus Las Lagunillas, 23071 Jaén, Spain; ∥ Univ. Rennes, CNRS, Institut des Sciences Chimiques de Rennes (ISCR), UMR 6226, F-35000 Rennes, France

**Keywords:** pyrimidines, boron complexes, ESIPT, fluorescence, TD-DFT

## Abstract

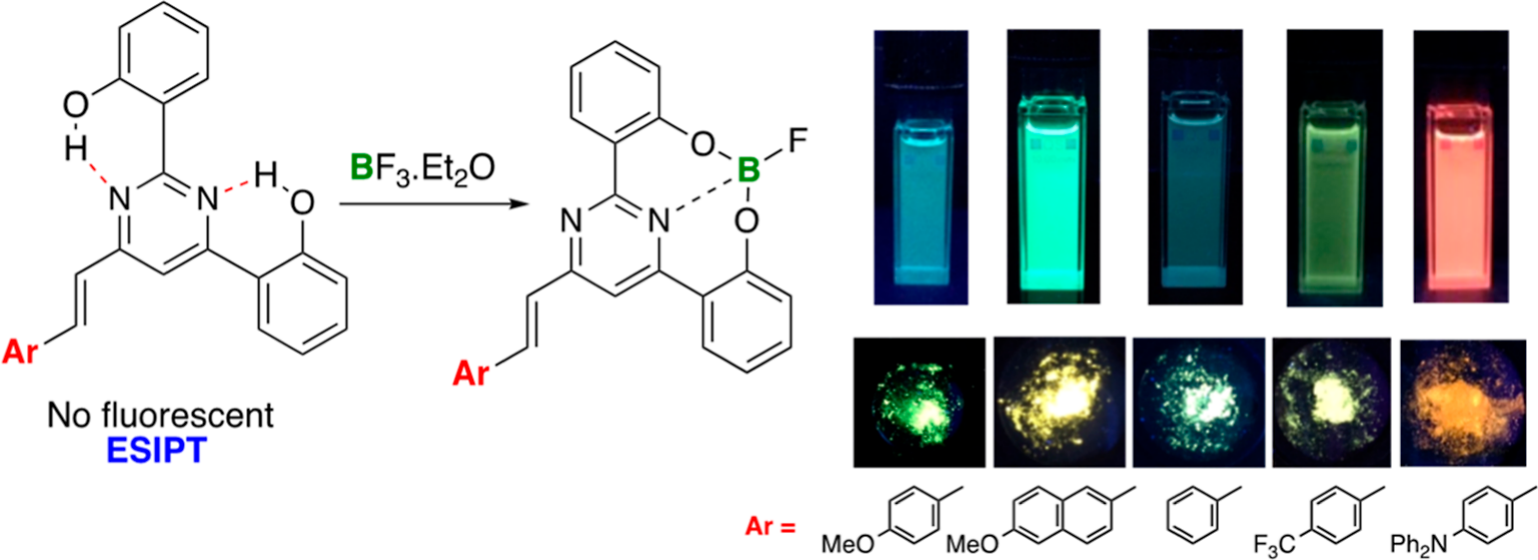

In this study, we present the synthesis and photophysical characteristics of a novel series of 6-arylvinyl-2,4-bis(2′-hydroxyphenyl)pyrimidines. These compounds exhibit nonemissive properties attributed to the potential occurrence of a excited-state intramolecular proton transfer process from the OH groups to the nitrogen atoms of the pyrimidine ring. The introduction of an acid for protonation of the pyrimidine ring results in a significant enhancement of the fluorescence response, easily perceptible to the naked eye. Notably, these molecules serve as intriguing rigid O^∧^N^∧^O ligands for boron chelation. The incorporation of boron atoms promotes structural planarity, increases rigidity, and successfully restores fluorescence in both solution and the solid state. Moreover, the photoluminescence was found to be strongly influenced by the nature of the end groups on the arylvinylene fragment, allowing for the modulation of the emission color and covering the optical spectrum from blue to red. Strong emission solvatochromism was observed in various solvents, a finding that supports the formation of intramolecular charge-separated emitting states, particularly when terminal electron-donating groups are present in the structure. X-ray diffraction analysis enables the determination of inter- and intramolecular interactions, as well as molecular packing structures, aiding in the rationalization of distinct luminescent behaviors in the solid state. All experimental findings are elucidated through extensive density functional theory (DFT) and time-dependent DFT calculations.

## Introduction

Four-coordinate organoboron compounds with π-conjugated structures have evolved as a promising class of light-emitting and electron-transporting materials due to their intense luminescence and high carrier mobility.^1^ These features have been found to open up great potential for practical application in optoelectronic devices such as organic light-emitting diodes (OLEDs), organic field-effect transistors, as well as photoresponsive, sensory, and imaging materials.^2−5^ In tetracoordinated boron chelates, the boron atom enhances the planarity of the structure, leading to improved conjugation and charge transfer throughout the π system.^6−8^ Chelation also induces rigidity into the framework and higher fluorescence quantum yields, along with increased chemical and thermal stability. On the other hand, the π-conjugated structure can be suitably modified at either the ligands or the boron centers for the effective optimization and fine-tuning of the properties.

In this context, 2,6-bis(2′-hydroxyphenyl)pyridine derivatives appear as interesting O^∧^N^∧^O ligand for boron chelation. The corresponding complexes exhibit strong emission in both solution and the solid state and can be used as emitting materials to fabricate highly efficient multicolor^9^ and white electroluminescent devices.^10,11^ Recently, a novel class of four-coordinate fluoroboron-containing push–pull structures incorporating this ligand as an acceptor has emerged as compelling thermally activated delayed fluorescence compounds. These materials exhibit high efficiency in OLEDs while demonstrating prolonged operational stabilities.^12^

Building upon these findings and drawing from our experience with pyrimidine-based donor–acceptor chromophores,^13−15^ in this contribution, we focus our attention on the study of structurally related 2,4-bis(2′-hydroxyphenyl)pyrimidines. The presence of a second nitrogen atom within the central ring of the ligand offers particularly interesting prospects. Pyrimidine derivatives substituted with electron-donating fragments through π-conjugated linkers are highly fluorescent and have demonstrated to be highly sensitive to environmental stimuli.^16,17^ The potential for protonation, complexation, and hydrogen bonding of the pyrimidine ring is an excellent tool for developing new sensing and luminescent materials.

On the other hand, 2-(2′-hydroxyphenyl)pyrimidine derivatives characterized by short OH···N intramolecular hydrogen bonds represent a class of compounds capable of displaying excited-state intramolecular proton transfer (ESIPT) reactions upon photoexcitation, a process that has been extensively study in different molecules from both spectroscopic and theoretical perspectives in the last years.^18−22^ The inherent versatility in their synthetic design, the ability to fine-tune their properties, and the extreme sensitivity to both external and internal stimuli render them captivating substrates for a diverse range of practical applications. Recently, we reported that 2-(2′-hydroxyphenyl)pyrimidines featuring aryl or arylvinylene substituents at the 4- and 6- positions of the pyrimidine ring exhibit minimal to negligible luminescence both in solution and in the solid state. This phenomenon was elucidated by the occurrence of ESIPT from the OH group to the nitrogen atoms of the pyrimidine ring.^23^ The fluorescence response could be reversibly switched on by inhibiting the ESIPT process through protonation. Similarly, 2-(2′-hydroxyphenyl)-4-(1*H*-pyrazol-1-yl)-6-methylpyrimidine derivatives were found to lack luminescence in solution. Nevertheless, they exhibited dual emission associated with phosphorescence and fluorescence in the solid state. Phosphorescence is not related to the ESIPT process and takes place in the enol form of the molecule, whereas fluorescence occurs in the keto form.^24^

Herein, we have expanded our interest to encompass the synthesis and investigation of the photophysical properties of a novel series of 6-arylvinyl-2,4-bis(2′-hydroxyphenyl)pyrimidines. The incorporation of a second 2′-hydroxyphenyl group at the 4-position of the pyrimidine could facilitate the ESIPT process. As expected, these molecules demonstrated nonemissive characteristics and exhibited acidochromic behavior similar to that of their 2-(2′-hydroxyphenyl)pyrimidine counterparts. Nevertheless, of utmost significance is their capability to act as rigid tridentate O^∧^N^∧^O chelating ligands, enabling the synthesis of a novel class of four-coordinate donor–acceptor organoboron compounds in which fluorescence was successfully restored. The elucidation of the emissive behavior of these compounds has been achieved through density functional theory (DFT) calculations at the M06-2X/6-31 + G** level of theory.

## Results and Discussion

### Synthesis

**Scheme 1:**
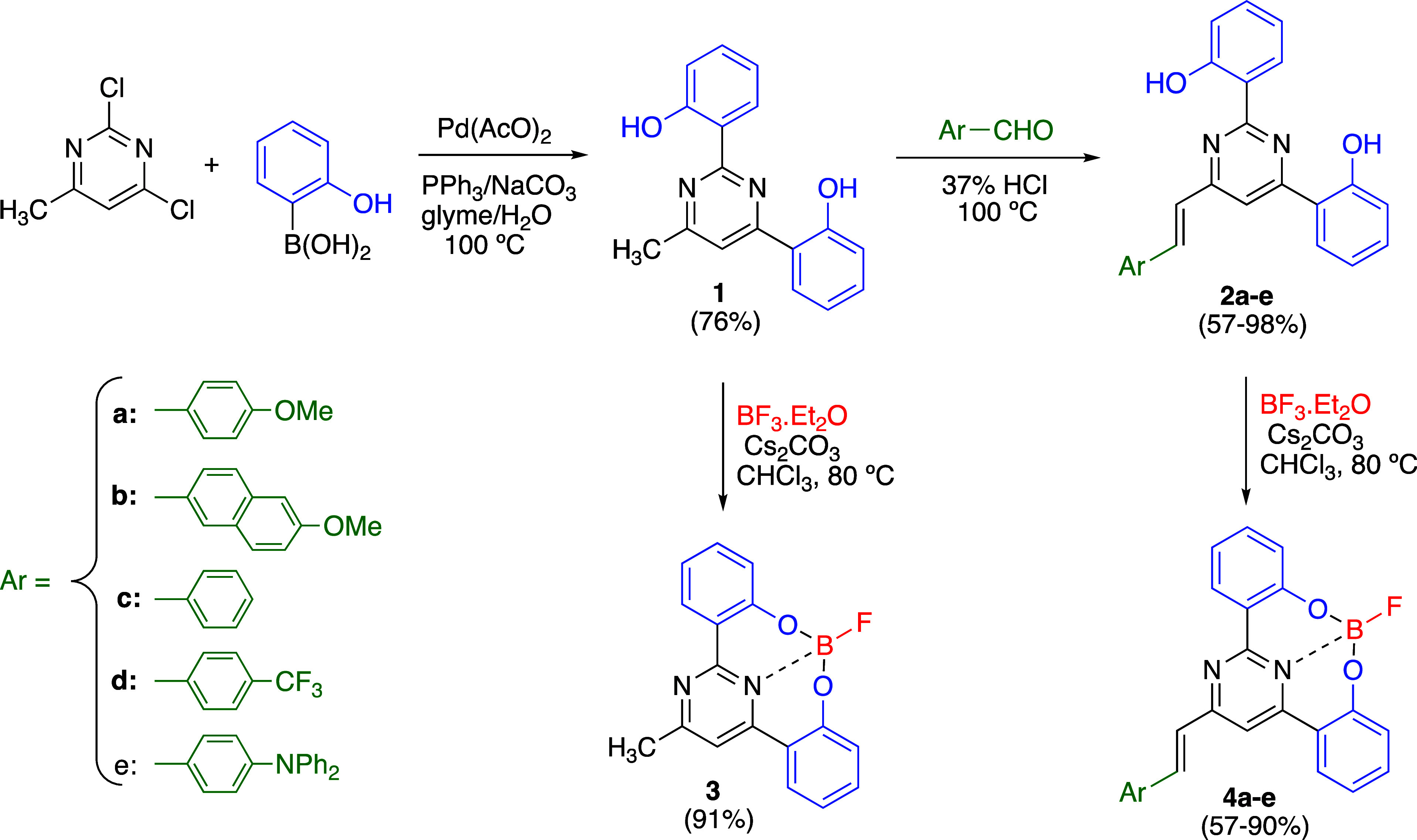
Synthesis of 2,4-Bis(2′-hydroxyphenyl)pyrimidines **1–2** and Their Corresponding Organoboron Complexes **3–4**

The 6-arylvinyl-2,4-bis(2′-hydroxyphenyl)pyrimidines were prepared from commercially available 2,4-dichloro-6-methylpyrimidine following the synthetic protocol outlined in Scheme 1. The Suzuki–Miyaura cross-coupling reaction with two equivalents of 2-hydroxyphenylboronic acid gave **1**, whereas compounds **2a**–**d** were obtained through subsequent Knoevenagel condensation with the appropriate aromatic aldehyde in acidic media. The expected ^3^*J*_H,H_ coupling constants of approximately 16 Hz for the vinylic protons in the ^1^H NMR spectra clearly supported the selective formation of an *E*-configured double bond. Finally, the synthesis of the corresponding organoboron complexes **4a**–**e** required treatment with BF_3_·Et_2_O in the presence of Cs_2_CO_3_. Similarly, complex **3** could be easily prepared from 2,4-bis(2′-hydroxyphenyl)-6-methylpyrimidine **1** (Scheme 1). Detailed synthetic procedures for all compounds, along with the corresponding analytical data, can be found in the Supporting Information (Figures S1–S42).

### X-ray Crystallography

The solid-state structures of the compounds significantly influence their physical properties. To understand these relationships, suitable single crystals of compounds **1**, **2a**, and **3** for X-ray diffraction were obtained in CH_2_Cl_2_/CH_3_CN (**2a**) and CH_2_Cl_2_/MeOH (**1** and **3**) solvent systems. Crystallographic data are provided in Table S1. These compounds exhibit distinct crystal packing arrangements; compound **1** crystallizes in the monoclinic space group *P*2_1_/*c*, **2a** crystallizes in the orthorhombic space group *P*2_1_2_1_2_1_, and the boron complex **3** crystallizes in the triclinic space group *P*1̅.

**Figure 1:**
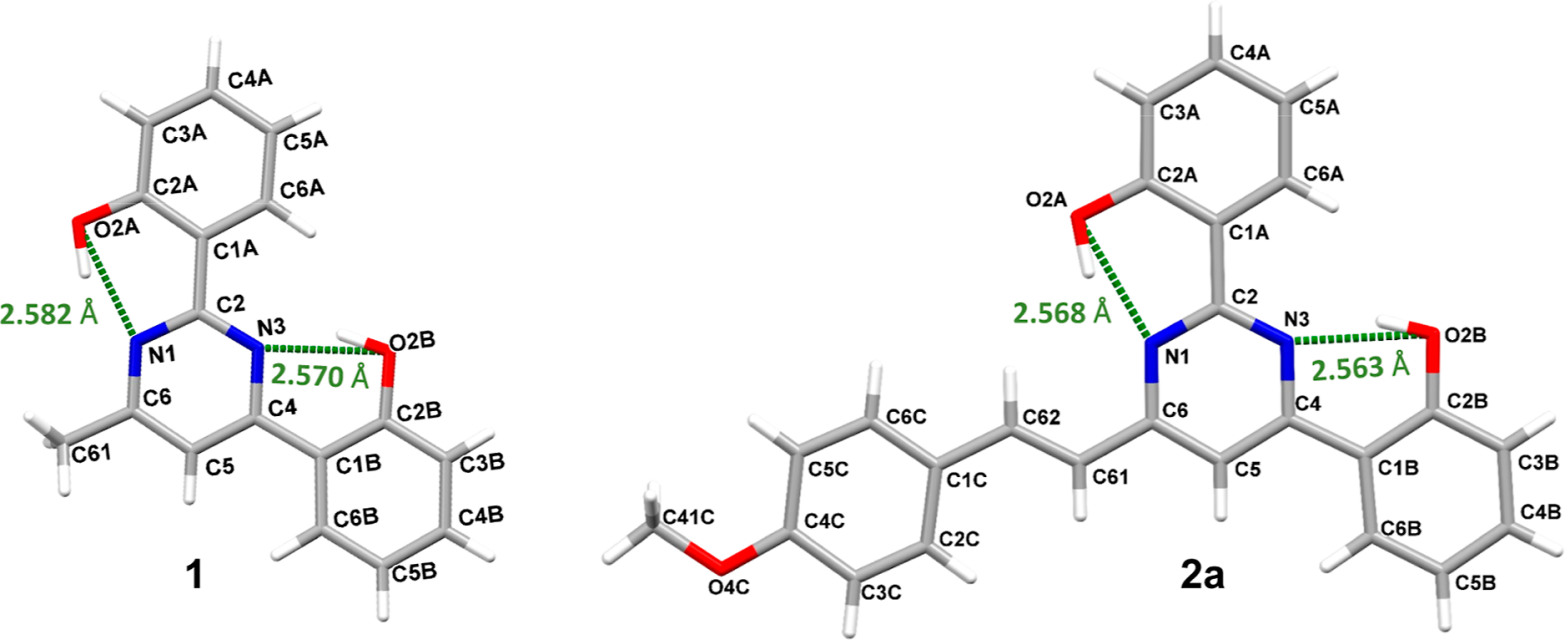
Atom-numbering scheme and molecular structures of compounds **1** and **2a** featuring intramolecular H-bonds.

The molecular structures of compounds **1** and **2a** are very similar (Figure 1) to each other, exhibiting a nearly planar conformation between the phenol units and the pyrimidine ring, with dihedral angles of 1–8° (**1**) and ca. 2° (**2a**). Additionally, intramolecular hydrogen bonds are present between the hydrogen atoms of the OH groups and the N atoms of the pyrimidine ring, with OH···N bond angles of 153° and hydrogen bond distances of approximately 2.56 Å.

**Figure 2:**
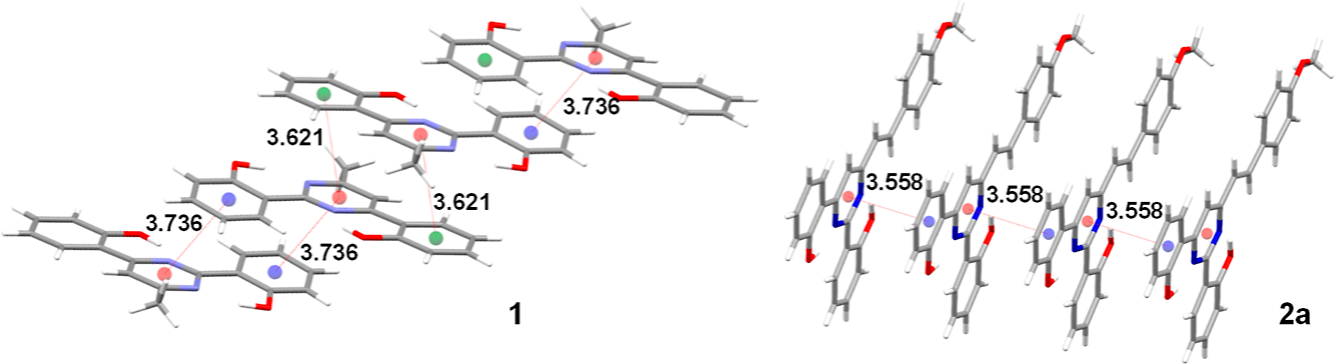
Molecular packing of **1** and **2a** illustrating π–π-interaction distances (in Å).

The crystal packing diagram presented in Figure 2 reveals that the arrangement in the solid state (for both compounds) involves π–π-interactions, in which the aromatic rings participate (selected geometrical features are given in Table S2).^25^ In compound **1**, the molecules are arranged in a face-to-tail fashion at distances of 3.6 and 3.7 Å, while a face-to-face arrangement is observed in compound **2a**, leading to a slight decrease in the distance between molecules (3.558 Å).

**Figure 3:**
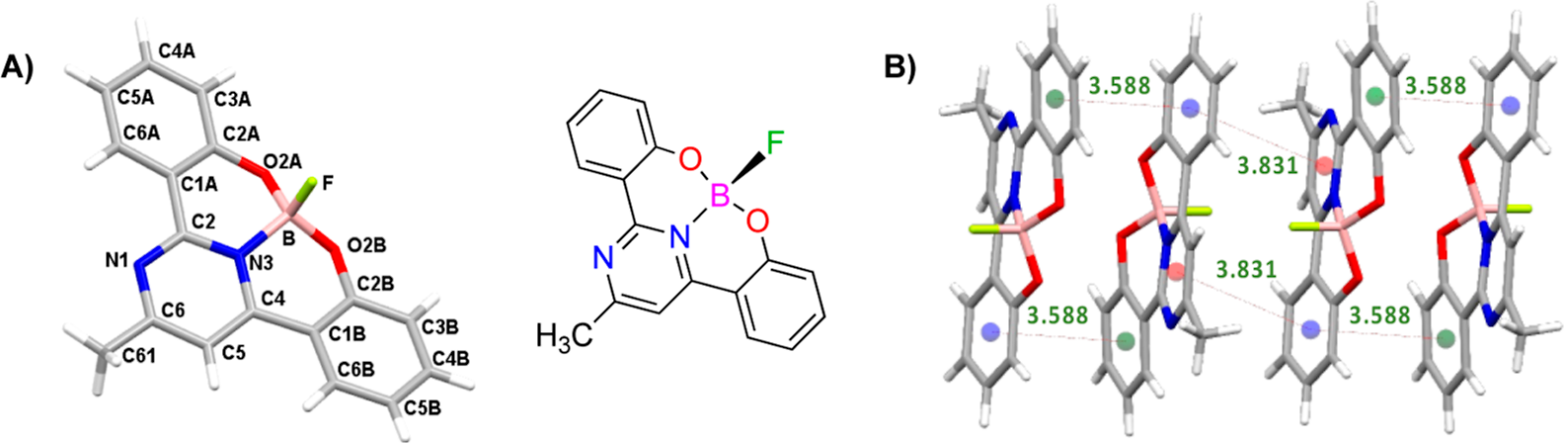
(A) Molecular structure of compound **3** and the rigid framework established through the boron coordination. (B) Packing diagram of **3** showing the π–π-interactions (in Å).

The boron center in compound **3** is four-coordinate and adopts a typical tetrahedral geometry (O, N, O, F). The ligand chelates the boron atom in a tridentate fashion through O2A, O2B, and N3 atoms, while a fluorine atom occupies the remaining coordination site (Figure 3A). The angles formed by the boron center and the donor atoms range between 108 and 111°, which are similar to the ideal tetrahedral geometry angle of 109°. The B–N bond length is 1.59 Å, a typical value for a B–N single bond (1.58 Å).

The structure forms a slightly distorted fused tetracyclic core, comprising the two phenolate rings and the two six-membered chelate rings. Distances in this compound closely resemble those of the free ligand **1**, with inter-ring torsion angles between the pyrimidine and phenolate rings of approximately 12°, although they remain essentially planar. The C–N (1.35 Å), C–O (1.35 Å), and C–C (1.40–1.46 Å) bond lengths within the boron heterocycle align with the expected values for single and double bonds of the respective atoms, indicating significant delocalization of the π-electron systems. It is noteworthy that the boron atom is noticeably displaced from the plane defined by the heterobicycle.^26^

The perpendicular orientation of the axial fluorine atom with respect to the π-conjugated tetracyclic plane prevents strong interactions, such as hydrogen bonds or other possible short-contact interactions within the molecules. In the crystalline packing of compound **3**, the molecules are arranged in an antiparallel orientation. Thus, dimers are formed by π–π interactions between the phenolate rings, with distances of 3.588 Å. These dimers further interact with each other through π-stacking between the pyrimidine and the phenolate rings of neighboring molecules, with the distance to the adjacent molecule being 3.831 Å (Figure 3B).^27^

### Photophysical Properties

The optical properties of compounds **1**–**4** were investigated by UV/vis and photoluminescence (PL) spectroscopy in CH_2_Cl_2_ solution at room temperature, as well as in the solid state. The spectra are depicted in Figures S43–S53, and the collected data are summarized in Table 1.

**Table 1:** UV/Vis and PL Data of Prepared Compounds^a^

	CH_2_Cl_2_				solid (powder)	
compd.	UV/vis λ_max_, nm (ε, mM^–1^·cm^–1^)	PL λ_max_, nm	Φ_F_^b^	Stokes shift^c^cm^–1^	PL λ_max_, nm	Φ_F_^b^
**1**	268 (31.2), 337 (17.2)					
**2a**	272 (33.5), 378 (45.1)					
**2b**	272 (48.5), 390 (45.8)					
**2c**	277 (41.8), 337 (42.5), 366 (34.5)^d^					
**2d**	280 (43.6), 308 (32.0)^d^, 332 (26.6)^d^, 366 (19.4)^d^					
**2e**	275 (79.6), 297 (65.4)^d^, 336 (37.7)^d^, 441 (78.4)	591	<0.01		682	<0.01
**3**	271 (28.2), **338** (16.0), 359 (14.2)^d^	441	0.17	6910	425	0.09
**4a**	260 (27.6), **403** (25.1)	484	0.06	4153	528	0.14
**4b**	276 (49.3), **415** (37.1)	522	0.44	4939	578	0.33
**4c**	277 (28.6), **352** (44.4), 391 (23.7)^d^	507	0.03	8685	508	0.03
**4d**	280 (13.1), **347** (17.8), 397 (7.1)^d^	521	0.01	9625	521	0.03
**4e**	275 (59.1), 335 (25.2)^d^, **483** (47.8)	649	0.61	5296	597	0.02

a All spectra were registered at room temperature (*c* = 1.23–4.50 × 10^–6^ M).

b Fluorescence quantum yield calculated with a Jasco ILF-835/100 mm integrating sphere.

c Stokes shift calculated from the lowest energy absorption (indicated in bold) and emission maxima.

d Shoulder.

In solution, all compounds exhibited absorption maxima in the violet-blue region of the spectrum, accompanied by additional bands of higher energy. Complexation (i.e., transition from **2** to **4**) resulted in the red-shift of the lower energy bands, associated with the flattening of the ligand frame and the involvement of the lone electron pair of one of the nitrogen atoms, which increases the electron deficiency of the pyrimidine ring. These bands can be attributed to intramolecular charge transfer (ICT) transitions through the double bond between the donor aryl groups and the acceptor pyrimidine moiety. Additionally, a red-shift was also observed with an increase in the electron-donating character of the Ar group. The spectral profile were similar for both free ligands and boron complexes. Only the absorption spectrum of **2d** exhibited a distinct profile due to the diminished donor–acceptor characteristics of the structure. This difference stems from the presence of the electron-withdrawing CF_3_ group and, consequently, the modest ICT nature of the primary π–π* transition after excitation (Figure S46).

Compounds **1** and **2** were nonfluorescent in both solution and the solid state, except for a subtle emission observed in **2e**. Similar to their analogs of 2-(2′-hydroxyphenyl)pyrimidine,^23^ the absence of emission can be explained by the possibility of an intramolecular proton transfer reaction from the OH groups to the nitrogen atoms of the pyrimidine ring, forming a keto form in the excited state. This keto tautomer may undergo a charge-transfer process leading to nonradiative deactivation.

**Figure 4:**
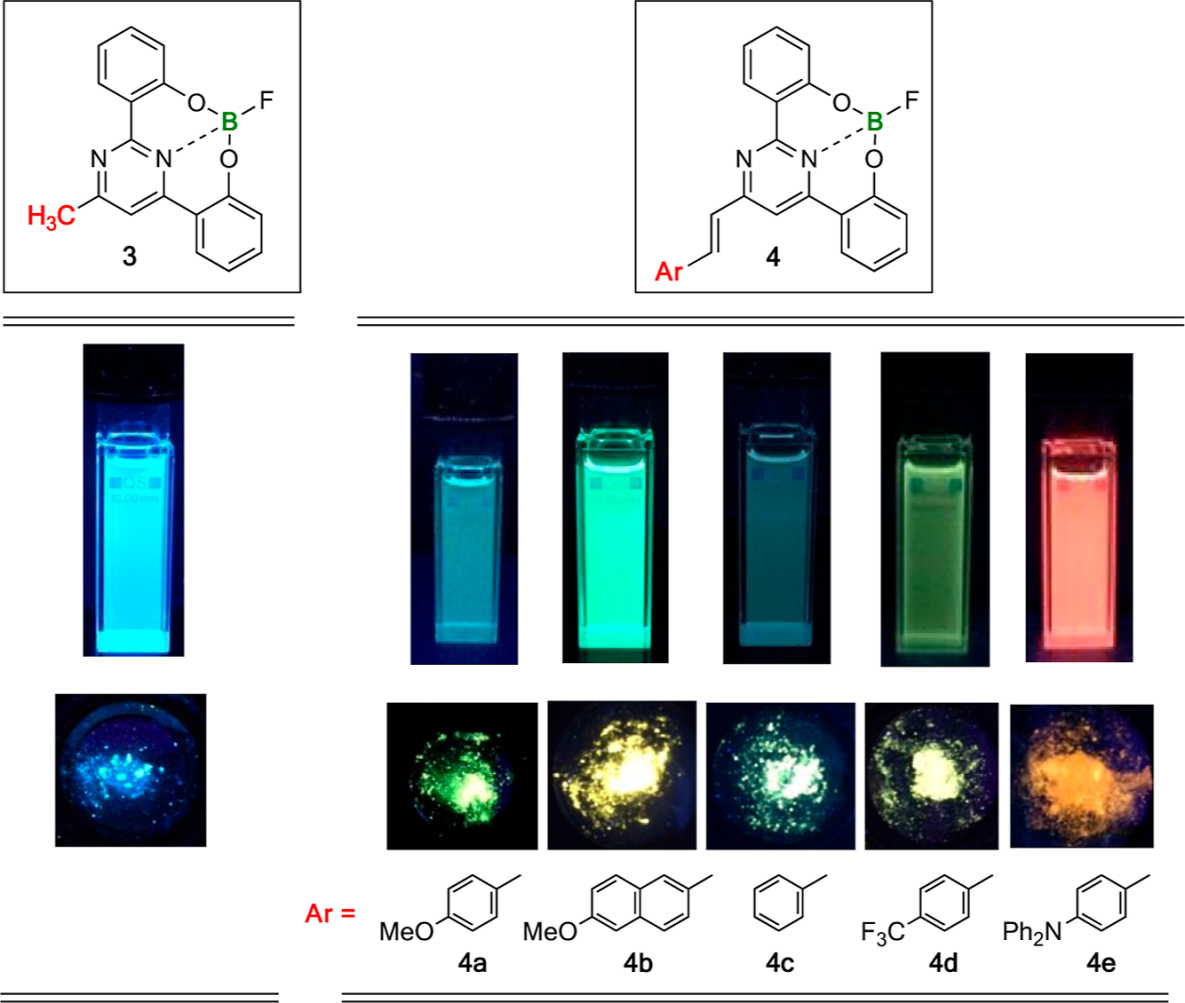
Color photographs of boron complexes **3** and **4** under 365 nm UV light in CH_2_Cl_2_ solution (top) and as powdered samples (bottom).

Unlike 2,4-bis(2′-hydroxyphenyl)pyrimidines **1** and **2**, their corresponding boron complexes **3**–**4** showed noticeable emission in the visible light region (Figures S48–S53). As anticipated, chelation induced rigidity into the framework, along with enhanced π-conjugation and efficient PL. In general, significant Stokes shifts occurred in all cases, which is also indicative of charge transfer. The presence of electron-donating groups on the aryl ring, as well as the extension of the π-conjugated system results in a red-shift of the emission wavelength (Table 1). Thus, it is possible to effectively fine-tune the emission color by appropriately choosing the substituent, covering the entire optical spectrum from blue to red (Figure 4). In solution, the highest fluorescence quantum yields (Φ_F_) were observed for complexes **4b** (44%) and **4e** (61%). However, their Φ_F_ were substantially lower in the solid state, particularly in **4e**, where a decrease from 61 to 2% was observed. The π–π stacking interactions described for compound **3** could also occur in compounds **4b** and **4e**, potentially accounting for this drop, as they all contain the rigid boron platform. These π–π interactions may also be responsible for the observed blue shift in the emission of compounds **3** and **4e** from solution to solid state as a result of short-range electronic couplings. These features are typical in H-type aggregates, where the decrease in emission is accompanied by a hypsochromic shift in the emission wavelength. Nevertheless, the absence of crystals does not definitively allow us to draw conclusive conclusions.

### Solvatochromism

**Figure 5:**
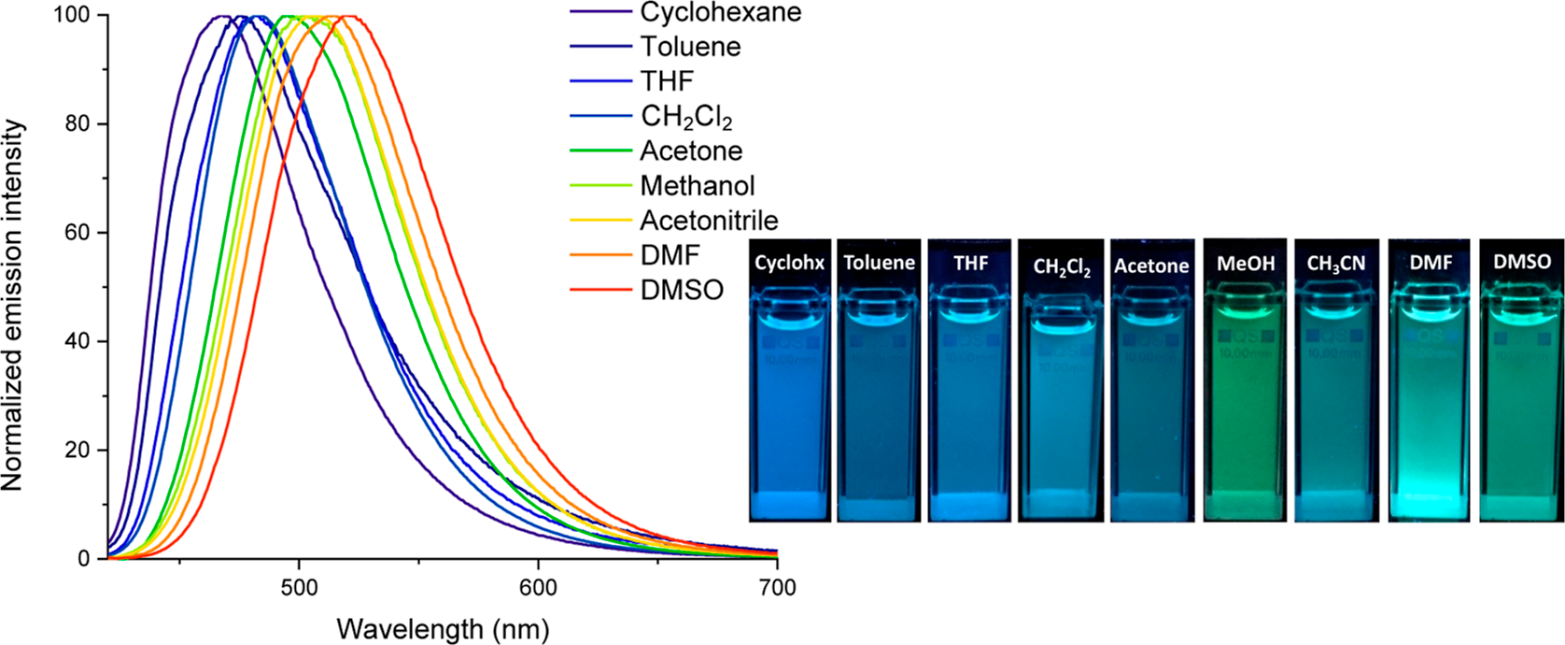
Left: normalized emission spectra of **4a** in various solvents. Right: color photographs under 365 nm UV light.

Emission solvatochromism emerges as one of the potential methods for evaluating ICT.^28,29^ As illustrative examples, we investigated the emission properties of complexes **3**, **4a**, **4d**, and **4e** in solvents of varying polarity (Table 2). A distinct positive solvatofluorochromism (red-shift) was observed with increasing solvent polarity for **4a**, indicating a highly polar excited state. The maximum emission wavelength at λ_em_ = 468 nm in the least polar solvent (cyclohexane) was red-shifted by 53 nm (Δν_em_ = 2174 cm^–1^) in DMSO (λ_em_ = 521 nm), resulting in a color change from blue to bluish-green (Figure 5). The strong donor NPh_2_ group in **4e** led to a more pronounced color transformation, transitioning from green in less polar solvents (cyclohexane, toluene) to orange-red in moderately polar solvents (THF, CH_2_Cl_2_, acetone), and ultimately to deep red in highly polar solvents (acetonitrile, DMF, DMSO) (Δλ_em_ = 219 nm, Δν_em_ = 5970 cm^–1^). In this scenario, an increase in solvent polarity resulted in a broad, structureless emission and a gradual decrease in fluorescence intensity (Figure S54). In contrast, the position of the emission band for complex **3** was not significantly affected (Δλ_em_ = 27 nm, Δν_em_ = 1356 cm^–1^), indicating a lower ICT due to the absence of the donor arylvinylene group (Figure S55). A similar trend was observed for compound **4d**, attributed to the presence of the acceptor CF_3_ group. Unfortunately, the low Φ_F_ of **4d** and the aforementioned loss of intensity prevented the registration of spectra at low concentration in the most polar solvents (Figure S56). Table S3 shows the dipole moment values calculated in the first excited state in CH_2_Cl_2_ solution. As can be seen, high values are predicted for compounds **4a** (13.9 D) and **4e** (15.6 D), which could explain the observed solvatochromism, while a lower value (7.7 D) was predicted for compound **3**.

**Table 2:** Emission Maxima of **3**, **4a**, **4d**, and **4e** in Various Solvents

comp	cyclohexane (30.9)^a^	toluene (33.9)^a^	THF (37.4)^a^	CH_2_Cl_2_ (40.7)^a^	acetone (42.2)^a^	MeOH (55.4)^a^	MeCN (45.6)^a^	DMF (43.2)^a^	DMSO (45.1)^a^
**3**	433	441	443	441	448	447	457	454	460
**4a**	468	476	482	484	496	504	506	513	521
**4d**	495	519	525	521					
**4e**	506, 536	554	631	649	686	699	723	716	725

a *E*_T_(30) Reichardt empirical solvent polarity parameter in kcal mol^–1^.

While there is generally a good correlation of the emission maxima with the solvent-dependent Δ*E*_T_(30) Reichardt’s polarity scale,^30^ the results cannot be explained solely on the basis of solvent polarity when methanol is used. This suggests that specific interactions, such as hydrogen bonding between the molecule and the surrounding solvent in the excited state, may play an important role, causing the excited state to become more energetic. On the other hand, the absorption spectra were nearly independent of solvent polarity, except for slight, irrelevant changes that indicated an insignificant electronic interaction between the donor and acceptor moieties in the ground state.

### Computational Insights

The fluorescence behavior was thoroughly investigated from a theoretical standpoint by conducting DFT and time-dependent DFT (TD-DFT) calculations using PBE0/6-31 + G** and M06-2X/6-31 + G** in CH_2_Cl_2_ solution, as well as in the crystal when data were available. Solvent effects were included using the polarizable continuum model to account for implicit solvation.

Initially, the study focused on compounds **1** and **2a** to elucidate the origin of the null emission in solution and the role of ESIPT in the electronic relaxation. Their molecular structure was fully optimized in CH_2_Cl_2_ solution, considering various tautomeric forms. Additionally, our theoretical study included the most emissive boron complexes in solution: **3**, **4a**, **4b**, and **4e**. The optimized molecular geometries for S_0_ and S_1_ are depicted in Figures S57–S62, while Table S4 presents the energy and relative energy (Δ*E*) in the ground state S_0_ and the first excited state S_1_.

As expected, the double enol form (EE) for **1** and **2a** was found to be the most stable in S_0_, consistent with the X-ray diffraction data. In the ground state, two moderate hydrogen bonds are predicted in compounds **1** and **2a** according to Jeffrey’s criteria,^31^ which could facilitate proton transfer in the excited state. The geometric parameters of the hydrogen bonds in S_0_ are approximately 150° (O–H–N bond angle) and 1.62 Å (O–H···N bond distance) with PBE0 and around 148° (O–H–N bond angle) and 1.68 Å (O–H···N bond distance) with M06-2X. The presence of two intramolecular hydrogen bonds causes the planarization of the molecule in that region. The six-membered cyclic structures formed differ from each other by the nitrogen involved in the hydrogen bond.

Upon excitation, an intramolecular proton transfer occurs and the hydrogen bond involving the transferred proton weakens, favoring the stabilization of keto forms. Two stable forms were found, namely KE and EK, wherein only one of the two protons is transferred to N1 (KE) or N3 (EK). The most pronounced situation occurs using PBE0, which predicts a considerable twist of the phenyl ring at position 4 (approximately 85°) in the EK tautomer (see Figures S63 and S64). The KE form is slightly more stable than the EK form (see Table S4). While the resulting stable forms are exclusively those presented in Table S4, it is noteworthy that the KK and EE tautomers were also considered as initial tautomeric forms in the geometry optimization of the excited state. The EE form of **2a** is also predicted in S_1_, although it is less stable than KE and EK. Geometry optimizations were also performed using CAM-B3LYP/6-31 + G** and ωB97x-D/6-31 + G** to validate these results, confirming KE and EK as the most stable tautomeric forms in solution (see Table S5).

**Figure 6:**
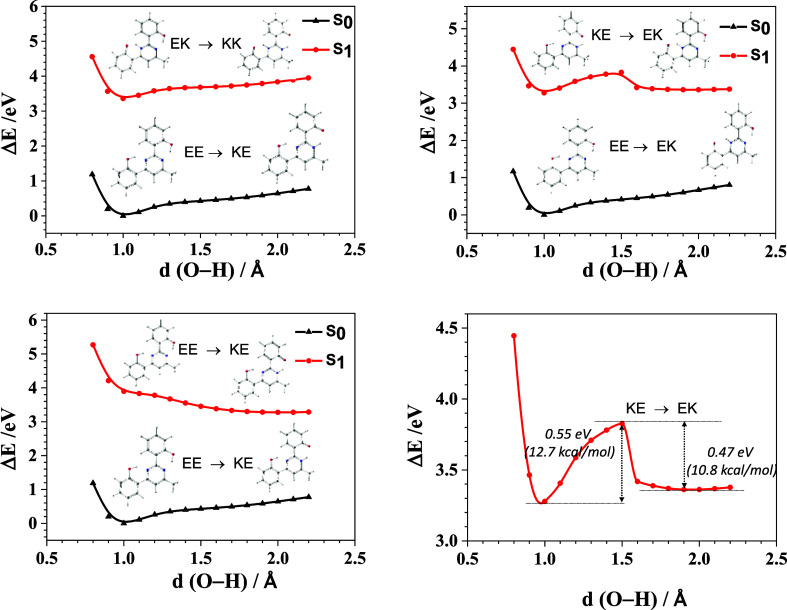
PES curves computed for compound **1** on the Δ*E* scale at the M06-2X/6-31 + G** level of theory in CH_2_Cl_2_ solution (relaxed scan). The enol (EE) and keto (KE) forms are indicated at short and long O–H distances, respectively. An enlarged view of the energy barriers for KE → EK in S_1_, calculated in the excited state, is presented at the bottom right.

**Figure 7:**
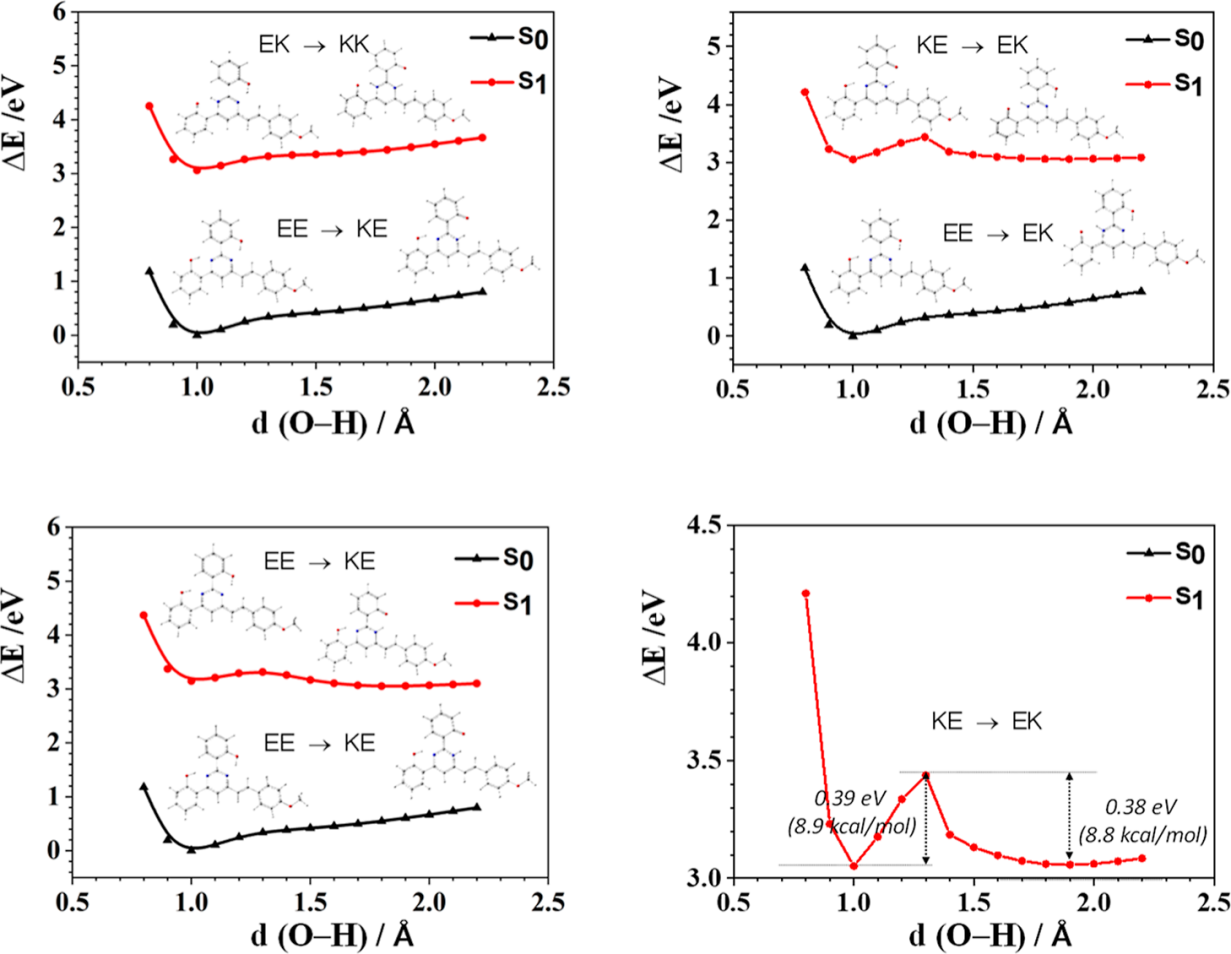
PES curves computed for **2a** on the Δ*E* scale at the M06-2X/6-31 + G** level of theory in CH_2_Cl_2_ solution (relaxed scan). The enol (E) and keto (K) forms are indicated at short and long O–H distances, respectively. An enlarged view of the energy barriers for KE → EK in S_1_, calculated in the excited state, is presented at the bottom right.

The relaxed potential energy scans (PES) were computed in CH_2_Cl_2_ solution by extending the O–H bond length toward the nitrogen atom of the pyrimidine ring. This permitted the analysis of the relative stabilization of the tautomeric forms in both the ground and excited states and the visualization of the energy barrier height of the intramolecular proton transfer. Figure 6 shows that as the O–H distance is increased in the excited state to facilitate ESIPT, the KE and EK forms experience stabilization, with the KE form being more stable. A similar result was obtained for compound **2a** in Figure 7.

**Figure 8:**
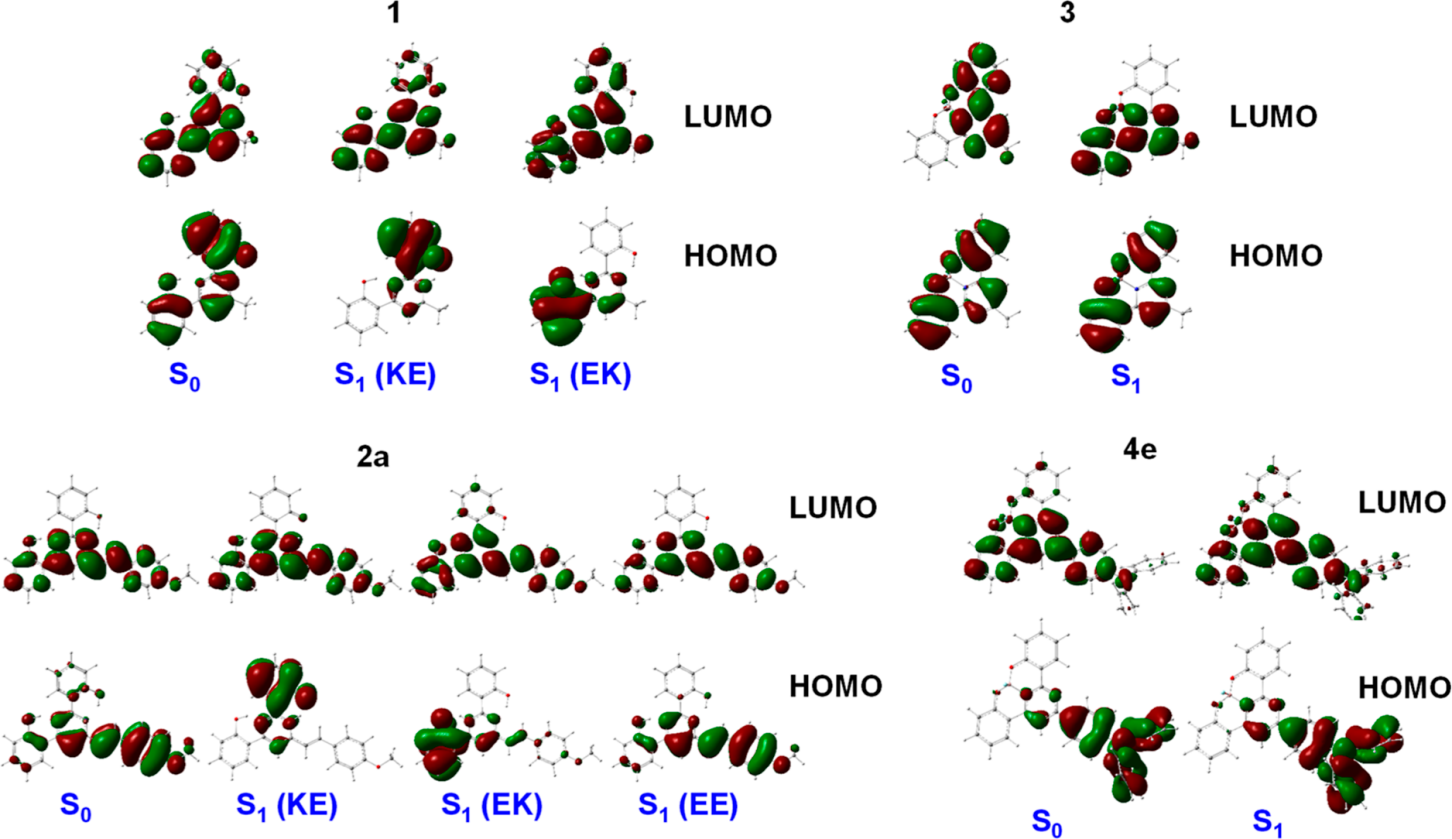
Molecular orbitals in CH_2_Cl_2_ solution calculated for the ground and excited states at the M06-2X/6-31 + G** level of theory (isocontour plots 0.02 au) for **1**, **2a**, **3**, and **4e**.

For compounds **4a**, **4b** and **4e**, planar structures were predicted in which the arylvinyl moiety is in the same plane as the boron platform, except the NPh_2_ group in **4e**. On the other hand, the vertical electronic transitions and oscillator strength (*f*) were also calculated in CH_2_Cl_2_ solution for compounds **1**, **2a**, **3**, **4a**, **4b**, and **4e** (EE tautomeric forms in **1** and **2a**). Table 3 reveals that PBE0 is in better agreement with the experimental absorption data than M06-2X. The maximum experimental absorption for compounds **1**, **2a**, **4a**, **4b**, and **4e** has been assigned to the lowest energy transition S_0_ → S_1_. This is predicted to be the strongest (except for compound **1** with PBE0) with a high contribution of HOMO → LUMO and therefore ICT character. However, the maximum of the absorption band observed in compound **3** at 338 nm has been assigned to the S_0_ → S_2_ transition, while the shoulder at 359 nm corresponds to the S_0_ → S_1_ transition. The HOMO and LUMO molecular orbitals are illustrated in Figures 8 and S65. In compound **1**, the HOMO is located over the phenol rings, while the LUMO is more localized on the pyrimidine ring. A similar electronic distribution for HOMO and LUMO is observed in compound **3**, where the oxygen atoms are coordinated to boron. In compounds **2a** and **4a**–**4e**, the HOMO is located over the styryl arm, while the LUMO is more localized on the pyrimidine ring.

**Table 3:** Experimental Maximum Absorption Wavelengths (λ_ab_^exp^), Calculated Vertical Electronic Transitions (λ_vert-ab_^calc^), Oscillator Strength (*f*), and Main Components of the S_0_ → S_1_ Transition (% Contribution) at the TD-PBE0/6-31 + G** and TD-M06-2X/6-31 + G** Levels of Theory in CH_2_Cl_2_ Solution^a^

compd.	λ_ab_^exp^ nm (eV)	λ_vert-ab_^calc^ nm (eV)	transition	*f*	% contribution
PBE0					
**1**	337 (3.68)	333 (3.72)	S_0_ → S_1_	0.12	H → L (87)
		330 (3.75)	S_0_ → S_2_	0.24	H – 1 → L (87)
		316 (3.93)	S_0_ → S_3_	0.21	H → L + 1 (89)
**2a**	378 (3.28)	395 (3.14)	S_0_ → S_1_	1.14	H → L (93)
		363 (3.42)	S_0_ → S_2_	0.10	H – 1 → L (93)
		331 (3.75)	S_0_ → S_4_	0.14	H → L + 1 (94)
**3**	359 (3.45)	360 (345)	S_0_ → S_1_	0.16	H → L (86), H → L + 1 (11)
	338 (3.67)	331 (3.75)	S_0_ → S_2_	0.13	H → L + 1 (62), H – 1 → L (23), H → L (12)
		325 (3.82)	S_0_ → S_3_	0.12	H – 1 → L (72), H → L + 1 (21)
**4a**	403 (3.08)	413 (3.00)	S_0_ → S_1_	1.20	H → L (90)
		387 (3.21)	S_0_ → S_2_	0.20	H – 1 → L (90)
		326 (3.80)	S_0_ → S_5_	0.19	H – 1 → L + 1 (84)
**4b**	415 (2.99)	442 (2.81)	S_0_ → S_1_	1.34	H → L (96)
		357 (3.47)	S_0_ → S_3_	0.11	H → L + 1 (64), H – 2 → L (32)
		346 (3.59)	S_0_ → S_5_	0.17	H – 3 → L (85)
**4e**	483 (2.57)	508 (2.44)	S_0_ → S_1_	1.30	H → L (98)
		392 (3.16)	S_0_ → S_3_	0.12	H – 1 → L (72), H → L + 1 (26)
		354 (3.50)	S_0_ → S_4_	0.11	H – 2 → L (95)
M06-2X					
**1**	337 (3.68)	300 (4.13)	S_0_ → S_1_	0.42	H – 1 → L (55), H → L (25), H → L + 1 (12)
		291 (4.26)	S_0_ → S_2_	0.29	H → L (41), H → L + 1 (37), H – 1 → L (11)
**2a**	378 (3.28)	349 (3.55)	S_0_ → S_1_	1.36	H → L (90)
		306 (4.05)	S_0_ → S_2_	0.15	H – 1 → L (38), H – 2 → L (37)
		289 (4.29)	S_0_ → S_3_	0.32	H – 1 → L + 1 (50), H → L + 1 (22), H – 2 → L + 1 (10)
**3**	359 (3.45)	319 (3.88)	S_0_ → S_1_	0.31	H → L (58), H → L + 1 (19), H – 1 → L (16)
	338 (3.67)	296 (4.19)	S_0_ → S_2_	0.31	H – 1 → L (61), H → L (17), H – 1 → L + 1 (13)
		261 (4.75)	S_0_ → S_4_	0.11	H – 1 → L + 1 (52), H – 2 → L + 1 (21)
**4a**	403 (3.08)	367 (3.38)	S_0_ → S_1_	1.43	H → L (86)
		328 (3.78)	S_0_ → S_2_	0.20	H – 1 → L (74)
		296 (4.19)	S_0_ → S_3_	0.13	H – 1 → L + 1 (43), H – 2 → L (38)
**4b**	415 (2.99)	379 (3.27)	S_0_ → S_1_	1.67	H → L (83)
		333 (3.72)	S_0_ → S_2_	0.10	H – 1 → L (74)
		297 (4.17)	S_0_ → S_4_	0.17	H – 1 → L + 1 (39), H – 3 → L (22), H – 2 → L (17)
**4e**	483 (2.57)	424 (2.93)	S_0_ → S_1_	1.54	H → L (84)
		336 (3.69)	S_0_ → S_2_	0.16	H – 1 → L (75)
		297 (4.17)	S_0_ → S_5_	0.24	H – 1 → L + 1 (39), H – 2 → L (34)

a EE tautomeric forms in **1** and **2a**.

Additionally, the theoretical emission was calculated from the optimized excited states using the state-specific solvation (specific solvation (SS)), linear response (LR), and corrected LR approaches (cLR) at the TD-M06-2X/6-31 + G** and TD-PBE0/6-31 + G** levels of theory (see Tables 4 and S6). Table 4 shows the results from the methods that provided values closest to the experimental data. The predicted values for the S_1_ → S_0_ transition with M06-2X are in good agreement with the experimental observations, except for compound **3**, where a greater deviation is observed using both the M06-2X and PBE0 methods with the SS approach. A large red shift is observed in the calculated S_1_ → S_0_ transition for the KE form compared to the EK form in compounds **1** and **2a**. The predicted low oscillator strength (*f* < 0.2) for the KE tautomeric form using M06-2X (SS approach) and values close to zero (0.05–0.00) using PBE0 (LR approach), combined with the greater stability of this tautomer, could partly explain the absence of emission from these compounds in solution. However, low *f* values alone are not sufficient to fully explain the relaxation mechanism, and other alternative nonradiative processes might be involved. On the other hand, the lower stability of the EE form in S_1_ and the high value for *f* (approximately 1.5) lead us to dismiss this form as an emissive state in solution. For compounds **4a**, **4b**, and **4e**, a red-shift was also predicted, as observed experimentally, with an increase in the electron-donating character of the Ar group.

**Table 4:** Experimental (λ_em_^exp^) and Calculated (λ_vert-em_^calc^) Maximum Emission Wavelengths for the S_1_ → S_0_ Transition at the TD-M06-2X/6-31 + G** (SS) and TD-M06-2X/6-31 + G** (LR) Levels of Theory in CH_2_Cl_2_ Solution

compd.	S_1_	λ_em_^exp^ nm (eV)	Φ_F_	λ_vert-em_^calc^ nm (eV)	*f*	% contr.	λ_vert-em_^calc^ nm (eV)	*f*	% contr.
				TD-M06-2X/6-31 + G** (SS)			TD-PBE0/6-31 + G** (LR)		
**1**	KE			680 (1.83)	0.18	H → L (94)	747 (1.66)	0.05	H → L (99)
	EK			558 (2.22)	0.27	H → L (90)	1155 (1.07)	0.00	H → L (97)
**2a**	KE			883 (1.41)	0.10	H → L (96)	795 (1.56)	0.02	H → L (100)
	EK			597 (2.08)	0.70	H → L (92)	1330 (0.93)	0.00	H → L (99)
	EE			430 (2.89)	1.64	H → L (96)	434 (2.86)	1.54	H → L (97)
**3**		441 (2.81)	0.17	369 (3.37)	0.44	H → L (87)	463 (2.68)	0.04	H → L (98)
**4a**		484 (2.56)	0.06	446 (2.78)	1.75	H → L (95)	447 (2.78)	1.60	H → L (97)
**4b**		522 (2.38)	0.44	497 (2.50)	2.07	H → L (93)	482 (2.57)	1.76	H → L (97)
**4e**		649 (1.91)	0.61	566 (2.19)	1.90	H → L (90)	572 (2.17)	1.19	H → L (97)

**Figure 9:**
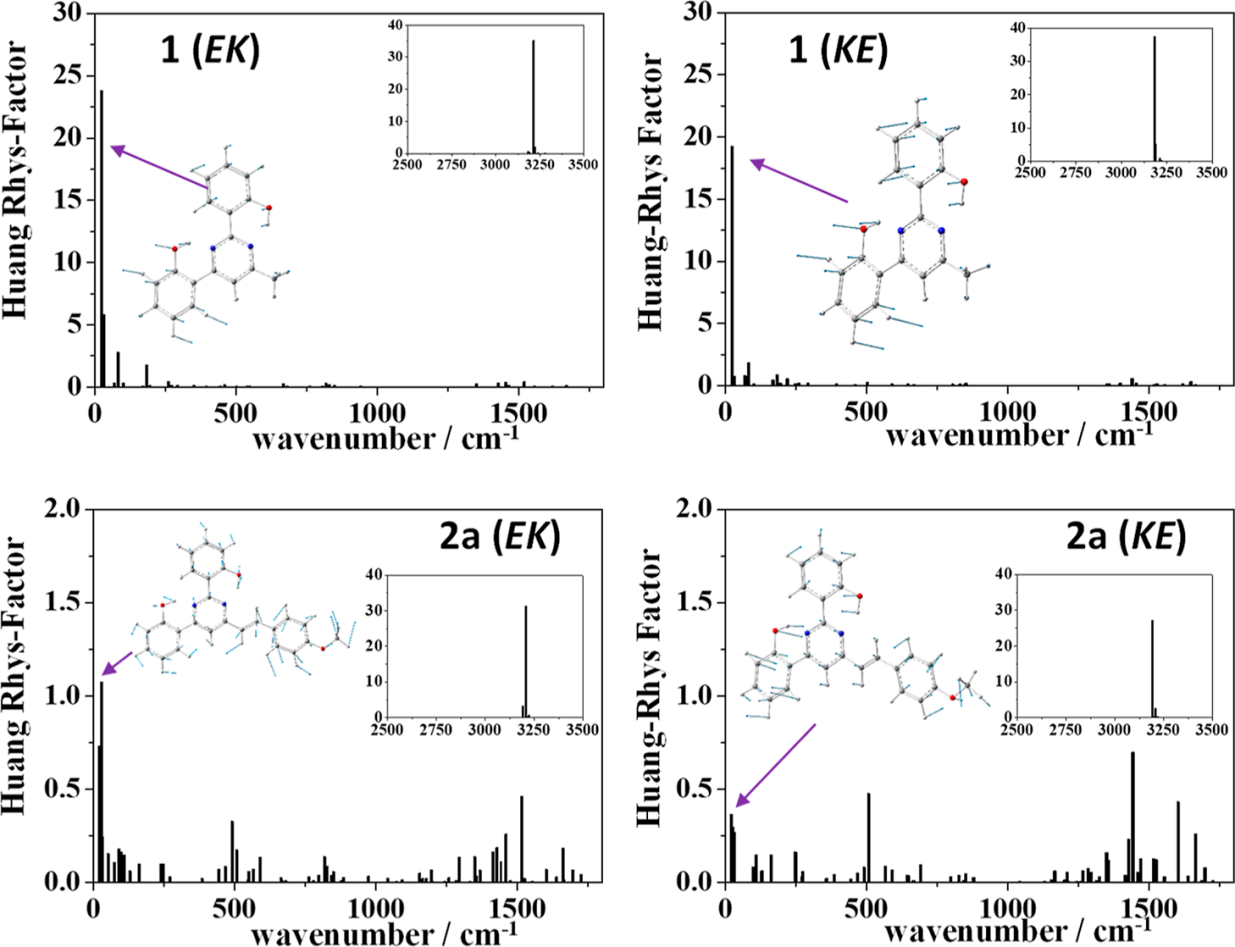
Huang–Rhys factors calculated for the ground state of compounds **1** and **2a** in CH_2_Cl_2_ at the M06-2X/6-31 + G** level of theory. The tautomeric form of the corresponding excited state S_1_ is indicated in parentheses.

The Huang–Rhys factors (HR) enable the prediction of the contribution of nonradiative vibrational relaxation from the excited state to the deactivation process in CH_2_Cl_2_ solution (see Table S7). Figure 9 shows notably large HR factors for the vibrational mode associated with the OH stretching for compounds **1** (3213 cm^–1^, HR = 35) and **2a** (3193 cm^–1^, HR = 27). Additionally, a vibrational mode in the low-energy region of compound **1** contributes slightly to the electronic relaxation (23 cm^–1^, HR = 24). This vibrational mode could trigger the proton transfer in the excited state and might explain the absence of emission by these compounds in solution. In contrast to the lack of emission observed for **1** and **2a** as a consequence of the ESIPT process, significant quantum yields of 44 and 61% were measured in solution for **4b** and **4e**, respectively (see Table 1), in agreement with the high values of *f* (approximately 2) predicted for these compounds (Table 4). These results are consistent with the small vibrational relaxation predicted for these compounds, indicated by the sum of HR factors of 2.2 and 3.2, respectively (see Table S7). A lower quantum yield of 17% aligns with the larger sum of HR factors (HR = 9.6) calculated for compound **3** (see Figure S66). However, unlike **4b** and **4e**, compound **4a** has a lower quantum yield (6%), despite also having low HR factors predicted for it (Figure S66). This behavior is similar to that observed in related pyrimidines,^13,14^ where increasing the electron-donating strength of the aryl group not only results in a red shift of the absorption and emission bands but also in a higher fluorescence quantum yield.

In any case, while the HR factors only account for nonradiative vibrational relaxation, the emission quenching in compounds **1** and **2** warrants further investigation and a deeper analysis of the radiative and nonradiative mechanisms involved in their photophysics. As reported in the literature for related systems, it may be necessary to explore other possible relaxation mechanisms, such as conical intersections and photoisomerization, the latter being particularly relevant for compounds with vinylene groups. This would provide a more comprehensive understanding of the complex photophysics of the studied compounds.^32−35^ Investigating these mechanisms would require a more advanced quantum-chemical study at the ab initio multireference level, such as CASSCF or XMS-CASPT2, which is beyond the scope of this work and could be addressed in future research.

**Figure 10:**
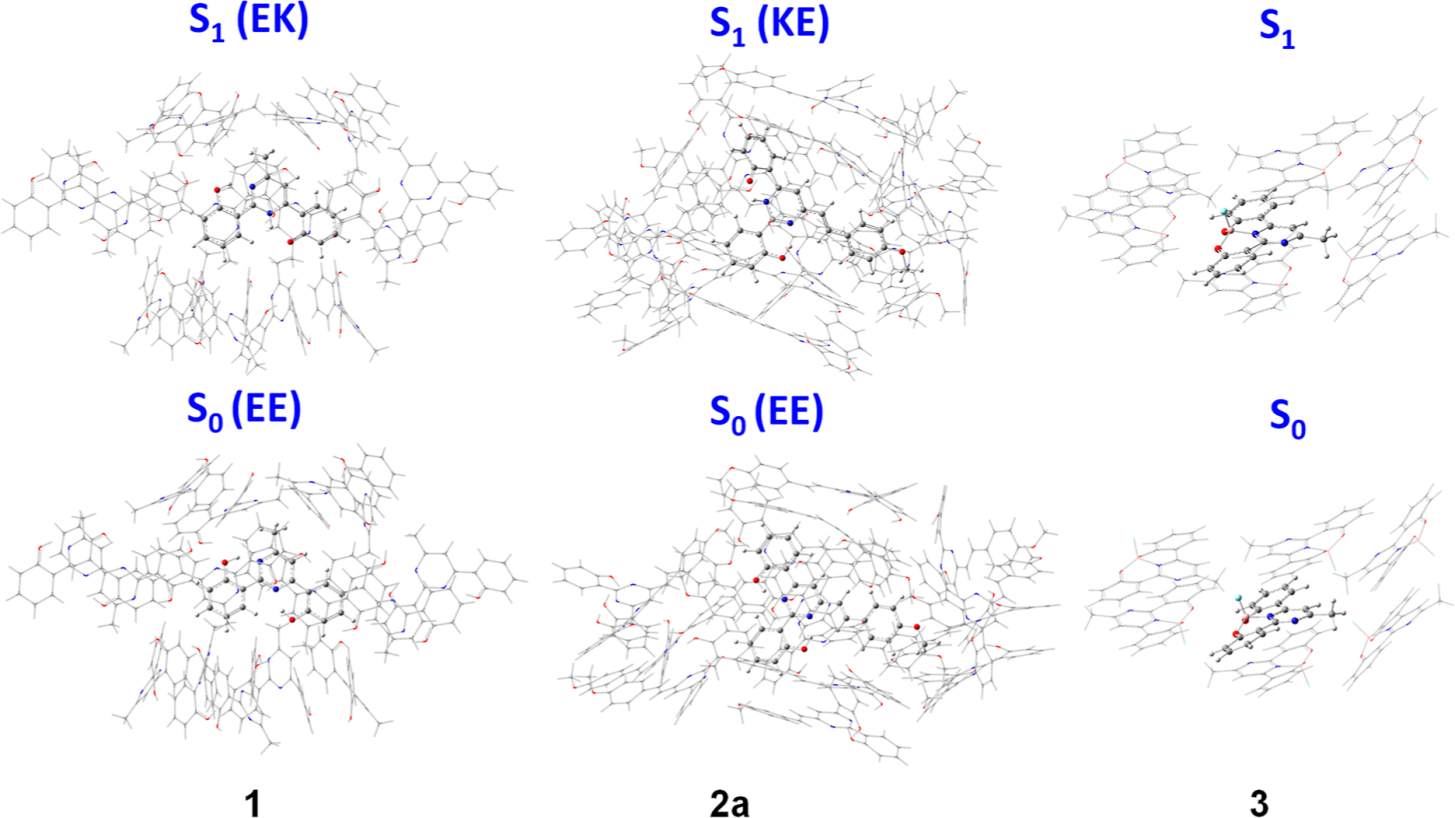
Molecular clusters computed at the QM/MM level for compounds **1**, **2a**, and **3**. The central molecule is treated as high level (M06-2X/6-31G**), while the surrounding molecules as low level (UFF41).

The possibility of ESIPT in the crystal was also investigated by performing TD-M062X/6-31 + G** calculations. As shown in Figure 10, a cluster of 16 molecules from the crystal structure was constructed for compounds **1** and **2a**. The central active molecule was optimized in the ground and first excited states using the ONIOM approach (see Supporting Information, Computational Details). According to the crystal structures, only the EE forms were considered as starting point. The molecular optimization of the first excited state resulted in the EK form for compound **1** and the KE form for compound **2a**, indicating that the ESIPT process would also be possible in the crystal. Table 5 lists the calculated emission of the central active molecule. The small oscillator strength predicted for this transition (*f* = 0.2) could justify the absence of emission in solid state.

**Table 5:** Experimental (λ_em_^exp^) and Calculated (λ_vert-em_^calc^) Maximum Emission Wavelengths for the S_1_ → S_0_ Electronic Transition at the M06-2X/6-31 + G** Level of Theory in Solid State

compd.	S_1_	λ_em_^exp^ nm (eV)	Φ_F_	λ_vert-em_^calc^ nm (eV)	*f*	% contr.
**1**	EK			709 (1.75)	0.02	H → L (98)
**2a**	KE			1564 (0.79)	0.02	H → L (96)
**3**		425 (2.92)	0.09	432 (2.87)	0.02	H → L (94)

On the other hand, a cluster of 7 molecules from the crystal structure was constructed for compound **3**. Again, the central active molecule was optimized in the ground and first excited state using the ONIOM approach. TD-M062X/6-31 + G** calculations predict the S_1_ → S_0_ transition at 432 nm, in good agreement with the observed emission band at 425 nm (Table 1). The decrease in emission from 17% in solution to 9% in the solid state might be explained by the π–π interactions described for compound **3** in the X-ray crystallography section, which usually quench the emission (see Figure 3b).

### Photophysical Properties of the Protonated Compounds

**Figure 11:**
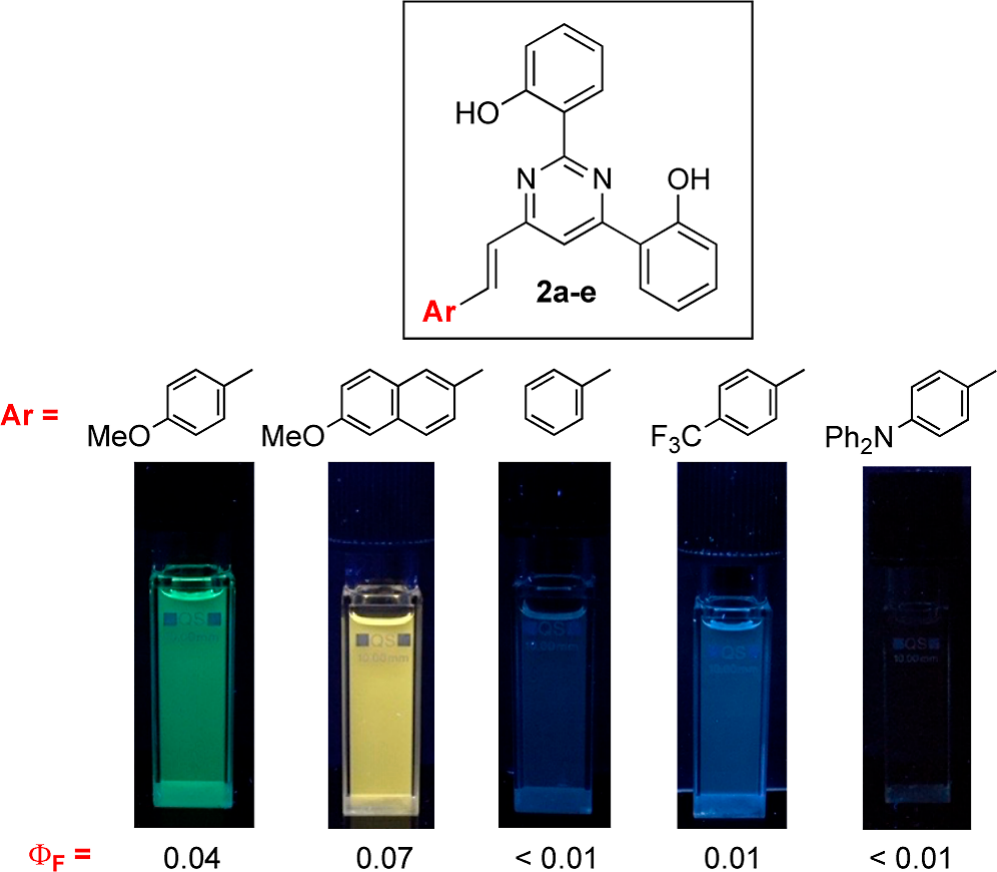
Color photographs of CH_2_Cl_2_ solutions of **2a**–**e** in acidic media (TFA, 1 M) under 365 nm UV light.

As mentioned in the introduction, it is feasible to induce substantial changes in the photophysical properties of push–pull pyrimidine derivatives by protonation (Table 6). In this respect, the fluorescence response of 6-arylvinyl-2,4-bis(2′-hydroxyphenyl)pyrimidines could be reversibly switched on by inhibiting the ESIPT process through protonation of the pyrimidine ring, effectively interrupting the nonradiative deactivation pathway of the excited state. The changes observed in the absorption and emission spectra after addition of trifluoroacetic acid (TFA) to the CH_2_Cl_2_ solutions are illustrated in Figures S43–S47. The presence of TFA (1 M) induced the appearance of a new red-shifted absorption band, which can be explained by the higher degree of ICT in the structure due to the reinforcement of the electron-withdrawing character of the pyrimidine ring.^36^ Although the neutral solutions did not exhibit luminescence, the acidic solutions showed emission bands ranging from blue to red. The highest fluorescence quantum yields were found for derivatives with moderate electron-donating groups **2a**–**b**, while **2c** and **2d** showed poor fluorescence (Φ_F_ ≤ 1%) although still detectable by the naked eye, which suggests a nonfluorescent nature of these protonated species.^37^ On the other hand, a greater ICT can explain the low emission of the protonated form of **2e** (Figure 11).

**Table 6:** UV/Vis and PL Data of CH_2_Cl_2_ Solutions of **2** in the Presence of TFA (1 M)^a^

	CH_2_Cl_2_ + TFA^b^		
compd.	UV/vis λ_max_, nm (ε, mM^–1^·cm^–1^)	PL λ_max_, nm	Φ_F_^c^
**2a**	325 (28.7), 447 (47.1)	528	0.04
**2b**	475 (45.5)	600	0.07
**2c**	325 (30.2), 404 (52.5)	461	<0.01
**2d**	357 (36.0), 378 (35.0) 396 (34.0)	461	0.01
**2e**	588 (51.3)	799	<0.01

a All spectra were registered at room temperature (*c* = 1.23–4.50 × 10^–6^ M).

b Data in the presence of TFA (1 M).

c Fluorescence quantum yield calculated with a Jasco ILF-835/100 mm integrating sphere.

**Figure 12:**
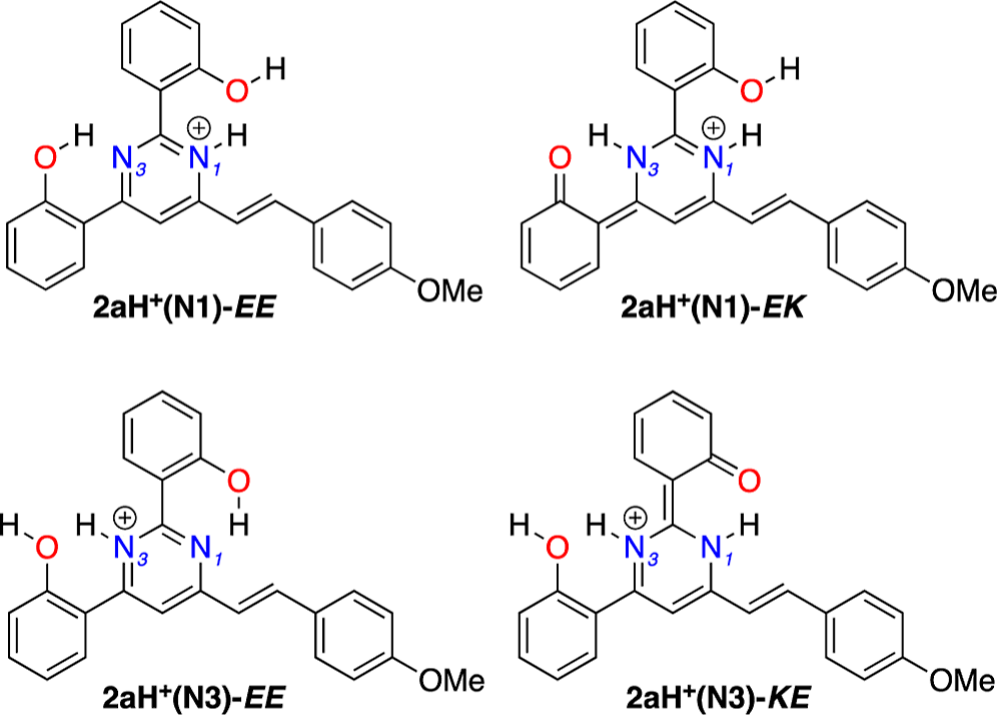
Scheme of protonation for compound **2a**.

TD-DFT calculations were performed on the protonated compound **2a**, exploring two protonation possibilities as illustrated in Figure 12, namely, **2aH**^**+**^**(N1)** for protonation at N1 and **2aH**^**+**^**(N3)** for protonation at N3. Molecular geometries for both ground and excited states were optimized at the M06-2X/6-31 + G** level of theory in CH_2_Cl_2_ solution (see geometrical parameters in Figures S67 and S68). The energy and relative energy (Δ*E*) are provided in Table S8. In the ground state S_0_, the protonated form **2aH**^**+**^**(N3)**-EE is slightly more stable than the protonated form **2aH**^**+**^**(N1)**-EE. On the other hand, in the first excited state S_1_, the forms **2aH**^**+**^**(N1)**-EE and **2aH**^**+**^**(N3)**-EE are more stable than their corresponding tautomers **2aH**^**+**^**(N1)**-EK and **2aH**^**+**^**(N3)**-KE (Δ*E* approximately 4–5 kcal/mol), respectively. Based on these results, emission is expected to occur from the EE tautomeric forms.

Table 7 lists the experimental absorption alongside the vertical electronic transitions. The theoretical predictions aligns well with the experimental observations, showing deviations of less than 0.3 eV. The lowest energy S_0_ → S_1_ transition is anticipated to be the strongest (with *f* approximately 1.4), displaying a high contribution from the HOMO → LUMO transition and, therefore, exhibiting ICT character. Figure S69 illustrates the HOMO and LUMO molecular orbitals for the protonated compounds.

**Table 7:** Experimental Maximum Absorption Wavelengths (λ_ab_^exp^), Calculated Vertical Electronic Transitions (λ_vert-ab_^calc^), and Oscillator Strength (*f*) Calculated at the TD-M062X/6-31 + G** Level of Theory in CH_2_Cl_2_ Solution

compd.	λ_ab_^exp^ nm (eV)	λ_vert-ab_^calc^ nm (eV)	transition	*f*	% contr. (≥10%)
**2aH**^**+**^**(N1)**-EE		409 (3.03)	S_0_ → S_1_	1.38	H → L (90)
		306 (4.05)	S_0_ → S_3_	0.29	H → L + 1 (74), H – 2 → L (14)
**2aH**^**+**^**(N3)**-EE	447 (2.77)	408 (3.04)	S_0_ → S_1_	1.46	H → L (92)
		339 (3.65)	S_0_ → S_2_	0.25	H – 1 → L (72)

**Figure 13:**
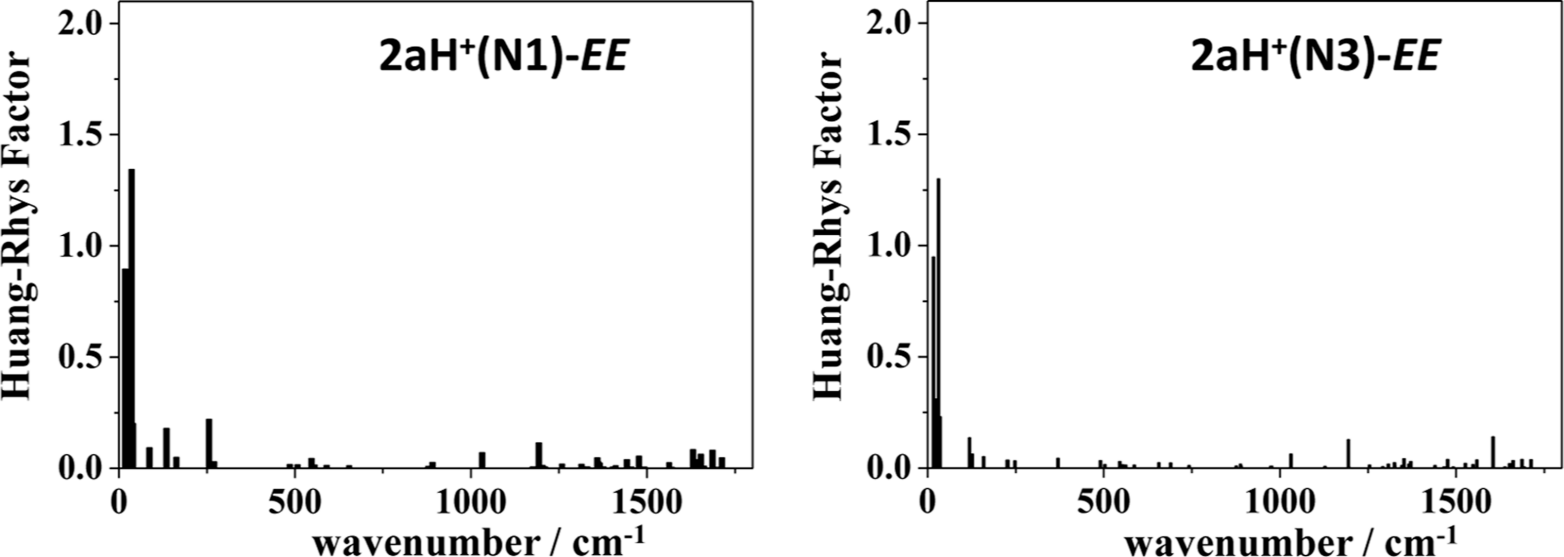
Huang–Rhys factors for the ground state of protonated forms **2aH**^**+**^**(N1)**-EE and **2aH**^**+**^**(N3)**-EE calculated at the M06-2X/6-31 + G** level of theory in CH_2_Cl_2_.

On the other hand, Table 8 presents the theoretical S_1_ → S_0_ electronic transition in conjunction with the experimental emission. Calculations predict that both protonation positions would result in emissive species, specifically **2aH**^**+**^**(N1)**-EE and **2aH**^**+**^**(N3)**-EE, each with a significant oscillator strength (*f* = 1.73), as well as the less stable form **2aH**^**+**^**(N1)**-EK (*f* = 1.46). Figure 13 illustrates small HR factors (HR < 1.5) calculated for the protonated forms **2aH**^**+**^**(N1)**-EE and **2aH**^**+**^**(N3)**-EE. The reduction in nonradiative vibrational relaxation compared to nonprotonated species could support the observed switch on fluorescence response for **2a** after the addition of acid (Φ_F_ = 4%) (see Table S9).

**Table 8:** Experimental (λ_em_^exp^) and Calculated (λ_vert-em_^calc^) Maximum Emission Wavelengths for the S_1_ → S_0_ Electronic Transition of Protonated Compound **2a** at the M06-2X/6-31 + G** Level of Theory in CH_2_Cl_2_ Solution

compound-S_1_	λ_em_^exp^ nm (eV)	λ_vert-em_^calc^ nm (eV)	*f*	% contr.
**2aH**^**+**^**(N1)**-EE	528 (2.35)	456 (2.72)	1.73	H → L (95)
**2aH**^**+**^**(N1)**-EK		503 (2.47)	1.46	H → L (92)
**2aH**^**+**^**(N3)**-EE		444 (2.80)	1.73	H → L (95)
**2aH**^**+**^**(N3)**-KE		1320 (0.94)	0.02	H → L (97)

## Experimental Section

### 2,4-Bis(2′-hydroxyphenyl)-6-methylpyrimidine (**1**)^38^

The reaction was performed in a screw-cap glass flask that was placed inside a metal heat transfer block. 2,4-Dichloro-6-methylpyrimidine (1 g, 6.13 mmol), 2-hydroxyphenylboronic acid (2.28 g, 16.55 mmol), and sodium carbonate (3.25 g, 30.65 mmol, dissolved in a minimum amount of water) were mixed with 1,2-dimethoxyethane (6 mL). Palladium acetate (140 mg, 0.62 mmol) and triphenylphosphine (320 mg, 1.22 mmol) were then added. The mixture was bubbled with argon for 5 min and heated at 100 °C for 60 h. The solvent was evaporated, water was added, and the mixture extracted with dichloromethane (×3). The combined organic extracts were dried (MgSO_4_), concentrated under vacuum, and filtered through a short pad of Celite and alumina. Finally, the solvent was evaporated and the crude product washed with boiling methanol to give a colorless solid (1.3 g, 76%). mp 145.6–147.7 °C. ^1^H NMR (CDCl_3_, 500 MHz) δ: 2.66 (s, 3H, CH_3_), 6.98 (m, 2H, ArH), 7.05–7.09 (m, 2H, ArH), 7.41–7.45 (m, 2H, ArH), 7.54 (s, 1H, pyr), 7.83 (dd, 1H, *J* = 8.0 Hz, *J* = 1.5 Hz, ArH), 8.15 (dd, 1H, *J* = 8.0 Hz, *J* = 1.5 Hz, ArH), 13.42 (broad s, 1H, OH), 13.86 (br s, 1H, OH). ^13^C NMR and DEPT (CDCl_3_, 125 MHz) δ: 165.4 (C), 165.1 (C), 162.8 (C), 161.1 (C), 161.0 (C), 134.0 (CH), 133.7 (CH), 128.1 (CH), 127.1 (CH), 119.4 (CH), 119.4 (CH), 118.9 (CH), 118.4 (CH), 117.3 (C), 116.7 (C), 111.7 (CH), 24.2 (CH_3_). IR (ATR) ν: 1597, 1534, 1354, 1296, 1242, 832, 780, 750, 736, 633 cm^–1^. MALDI-TOF MS (dithranol) *m*/*z*: 279.3 [M + H]^+^. HRMS (ESI) *m*/*z*: calcd for (C_17_H_15_N_2_O_2_) [M + H]^+^, 279.1128; found, 279.1124 (1 ppm).

### General Procedure for the Synthesis of (*E*)-2,4-Bis(2′-hydroxyphenyl)-6-arylvinylpyrimidines **2**

Reactions were performed in screw-cap glass flasks that were placed inside metal heat transfer blocks. A mixture of 2,4-bis(2′-hydroxyphenyl)-6-methylpyrimidine (**1**) and the corresponding aldehyde in concentrated hydrochloric acid (37%) was stirred at a 100 °C for 24 h. After cooling, saturated aqueous Na_2_CO_3_ was added until basic pH and the mixture was stirred at room temperature at least for 4 h. Then, the precipitated solid was collected by filtration, washed with water and methanol, and dried.

#### (*E*)-2,4-Bis(2′-hydroxyphenyl)-6-(4′-methoxystyryl)pyrimidine (**2a**)

Prepared from 139 mg (0.5 mmol) of **1** and 75 mg (0.55 mmol) of 4-methoxybenzaldehyde in 5 mL of hydrochloric acid. The solid was purified by trituration in boiling methanol. Yellow solid (190 mg, 96%). A further purification was obtained by crystallization from CHCl_3_/MeOH. mp decomposes >205 °C. ^1^H NMR (CDCl_3_, 500 MHz) δ: 3.87 (s, 3H, OCH_3_), 6.94–7.02 (m, 5H, CH= and ArH), 7.09 (A of AB_q_, 2H, *J* = 8.5 Hz, ArH), 7.42–7.46 (m, 2H, ArH), 7.58 (B of AB_q_, 2H, *J* = 8.5 Hz, ArH), 7.60 (s, 1H, pyr), 7.74 (B of AB_q_, 1H, *J* = 16.0 Hz, CH=), 7.88 (d, 1H, *J* = 8.0 Hz, ArH), 8.17 (d, 1H, *J* = 8.0 Hz, ArH), 13.74 (broad s, 1H, OH), 13.98 (br s, 1H, OH). ^13^C NMR and DEPT (CDCl_3_, 125 MHz) δ: 165.1 (C), 162.6 (C), 161.3 (C), 161.1 (C), 161.0 (C), 160.9 (C), 138.3 (CH), 134.0 (CH), 133.8 (CH), 129.6 (CH), 128.3 (CH), 127.7 (C), 127.1 (CH), 121.8 (CH), 119.5 (CH), 119.4 (CH), 118.9 (CH), 118.2 (CH), 117.4 (C), 116.9 (C), 114.5 (CH), 109.8 (CH), 55.4 (OCH_3_). IR (ATR) ν: 2633 (br), 1571, 1505, 1245, 1170, 844, 758, 634 cm^–1^. MALDI-TOF MS (dithranol) *m*/*z*: 397.4 [M + H]^+^. HRMS (ESI) *m*/*z*: calcd for (C_25_H_20_N_2_O_3_) [M]^+^, 396.1468; found, 396.1469 (0 ppm).

#### (*E*)-2,4-Bis(2′-hydroxyphenyl)-6-[2-(6′-methoxynaphthalen-2-yl)vinyl]pyrimidine (**2b**)

Prepared from 150 mg (0.54 mmol) of **1** and 123 mg (0.66 mmol) of 6-methoxy-2-naphthaldehyde in 5 mL of hydrochloric acid. In this case, the reddish-brown solid obtained was dissolved in dichloromethane and the solution was filtered through a short pad of alumina. After evaporation of the solvent, the yellow solid was purified by trituration in boiling methanol (160 mg, 66%). mp decomposes >185 °C. ^1^H NMR (CDCl_3_, 500 MHz) δ: 3.95 (s, 3H, OCH_3_), 7.00–7.05 (m, 2H, ArH), 7.10–7.15 (m, 3H, ArH), 7.18–7.20 (m, 1H, ArH), 7.19 (A of AB_q_, 1H, *J* = 16.0 Hz, CH=), 7.45–7.48 (m, 2H, ArH), 7.67 (s, 1H, pyr), 7.77–7.79 (m, 3H, ArH), 7.90–7.97 (m, 2H, ArH), 7.94 (B of AB_q_, 1H, *J* = 16.0 Hz, CH=), 8.20 (dd, 1H, *J* = 8.0 Hz, *J* = 1.0 Hz, ArH), 13.76 (s, 1H, OH), 13.99 (s, 1H, OH). ^13^C NMR and DEPT (CDCl_3_, 125 MHz) δ: 165.6 (C), 162.9 (C), 161.2 (C), 161.0 (C), 160.7 (C), 158.9 (C), 138.7 (CH), 135.5 (C), 134.0 (CH), 133.7 (CH), 130.3 (C), 130.1 (CH), 129.8 (CH), 128.8 (C), 128.3 (CH), 127.6 (CH), 127.1 (CH), 123.9 (CH), 123.2 (CH), 119.5 (CH), 119.5 (CH), 119.4 (CH), 119.0 (CH), 118.3 (CH), 117.6 (C), 117.0 (C), 110.0 (CH), 106.0 (CH), 55.4 (OCH_3_). IR (ATR) ν: 2722 (br), 1737, 1591, 1521, 1356, 1242, 1162, 1027, 967, 852, 752, 633 cm^–1^. MALDI-TOF MS (dithranol) *m*/*z*: 447.5 [M + H]^+^. HRMS (ESI) *m*/*z*: calcd for (C_29_H_22_N_2_O_3_) [M]^+^, 446.1625; found, 446.1629 (1 ppm).

#### (*E*)-2,4-Bis(2′-hydroxyphenyl)-6-styrylpyrimidine (**2c**)

Prepared from 150 mg (0.54 mmol) of **1** and 70 mg (0.66 mmol) of benzaldehyde in 5 mL of hydrochloric acid. The solid was purified by trituration in boiling methanol. Yellow solid (195 mg, 98%). mp decomposes >188 °C. ^1^H NMR (DMSO-*d*_6_, 500 MHz) δ: 7.01–7.08 (m, 4H, ArH), 7.42–7.52 (m, 6H, ArH), 7.82 (d, 2H, *J* = 7.0 Hz, ArH), 8.03 (B of AB_q_, 1H, *J* = 16.0 Hz, CH=), 8.05 (dd, 1H, *J* = 8.0 Hz, *J* = 1.5 Hz, ArH), 8.23 (s, 1H, pyr), 8.40 (dd, 1H, *J* = 8.0 Hz, *J* = 1.5 Hz, ArH), 11.85 (s, 1H, OH), 13.41 (s, 1H, OH). ^13^C NMR and DEPT (DMSO-*d*_6_, 125 MHz) δ: 163.3 (C), 162.8 (C), 161.7 (C), 160.0 (C), 158.1 (C), 137.7 (CH), 135.3 (C), 133.2 (CH), 133.0 (CH), 129.8 (CH), 129.3 (CH), 129.0 (CH), 128.7 (CH), 128.0 (CH), 125.8 (CH), 120.4 (C), 119.6 (CH), 119.1 (CH), 118.6 (C), 117.8 (CH), 117.5 (CH), 114.1 (CH). IR (ATR) ν: 2719 (br), 1587, 1568, 1355, 1298, 1242, 969, 837, 752, 691, 632 cm^–1^. MALDI-TOF MS (dithranol) *m*/*z*: 367.3 [M + H]^+^. HRMS (ESI) *m*/*z*: calcd for (C_24_H_18_N_2_O_2_) [M]^+^, 366.1363; found, 366.1365 (1 ppm).

#### (*E*)-2,4-Bis(2′-hydroxyphenyl)-6-[(4′-trifluoromethyl)styryl]pyrimidine (**2d**)

Prepared from 100 mg (0.36 mmol) of **1** and 70 mg (0.40 mmol) of 4-trifluoromethylbenzaldehyde in 6 mL of hydrochloric acid. The solid was purified by trituration in boiling hexanes. Yellow solid (150 mg, 96%). mp decomposes >190 °C. ^1^H NMR (CDCl_3_, 500 MHz) δ: 6.97–7.02 (m, 2H, ArH), 7.08 (ddd, *J* = 1.0 Hz, *J* = 5.0 Hz, *J* = 8.5 Hz, 2H, Ar), 7.18 (A of AB_q_, 1H, *J* = 16.0 Hz, CH=), 7.42–7.47 (m, 2H, ArH), 7.66 (s, 1H, pyr), 7.69 (A of AB_q_, 2H, *J* = 8.0 Hz, ArH), 7.73 (B of AB_q_, 2H, *J* = 8.0 Hz, ArH), 7.77 (B of AB_q_, 1H, *J* = 16.0 Hz, CH=), 7.87 (dd, 1H, *J* = 1.0 Hz, *J* = 8.0 Hz, ArH), 8.16 (dd, 1H, *J* = 1.5 Hz, *J* = 8.0 Hz, ArH), 13.46 (s, 1H, OH), 13.83 (s, 1H, OH). ^13^C NMR and DEPT (CDCl_3_, 125 MHz) δ: 166.0 (C), 163.1 (C), 161.3 (C), 160.9 (C), 159.8 (C), 138.3 (C), 136.4 (CH), 134.3 (CH), 134.0 (CH), 131.5 (q, *J* = 32.6 Hz, C), 128.3 (CH), 128.0 (CH), 127.1 (CH), 126.7 (CH), 126.0 (q, *J* = 3.6 Hz, CH), 123.9 (q, *J* = 269.7 Hz, CF_3_), 119.6 (CH), 119.5 (CH), 119.1 (CH), 118.3 (CH), 117.4 (C), 116.8 (C), 110.6 (CH). ^19^F NMR (CDCl_3_, 471 MHz) δ: −62.7. IR (ATR) ν: 3391 (br), 1568, 1322, 1241, 1065, 953, 855, 752, 633 cm^–1^. MALDI-TOF MS (dithranol) *m*/*z*: 435.5 [M + H]^+^. HRMS (ESI) *m*/*z*: calcd for (C_25_H_17_N_2_O_2_F_3_) [M]^+^, 434.1237; found, 434.1238 (0 ppm).

#### (*E*)-2,4-Bis(2′-hydroxyphenyl)-6-(4′-diphenylaminostyryl)pyrimidine (**2e**)

Prepared from 100 mg (0.36 mmol) of **1** and 147 mg (0.54 mmol) of 4-diphenylaminobenzaldehyde in 6 mL of hydrochloric acid. The resulting dark blue mixture was basified by the addition of saturated aqueous Na_2_CO_3_ and the precipitated black solid was collected by filtration, washed with water and methanol, and dried. The solid was then dissolved in dichloromethane and the solution was filtered through a small pad of Celite to obtain a yellow-orange solution. After evaporation of the solvent, the solid was triturated in boiling methanol and collected by filtration. Residual traces of the initial pyrimidine were detected by ^1^H NMR analysis, which were effectively removed by careful double recrystallization from THF/methanol. Yellow solid (110 mg, 57%). mp decomposes >264 °C. ^1^H NMR (CDCl_3_, 500 MHz) δ: 6.99 (A of AB_q_, 1H, *J* = 16.0 Hz, CH=), 6.96–7.04 (m, 2H, ArH), 7.06–7.09 (m, 4H, ArH), 7.11–7–15 (m, 2H, ArH), 7.16–7.18 (m, 4H, ArH), 7.31–7.35 (m, 4H, ArH), 7.42–7.47 (m, 2H, ArH), 7.50 (B of AB_q_, 2H, *J* = 8.5 Hz, ArH), 7.62 (s, 1H, pyr), 7.75 (B of AB_q_, 1H, *J* = 16.0 Hz, CH=), 7.89 (dd, 1H, *J* = 8.0 Hz, *J* = 1.5 Hz, ArH), 8.19 (dd, 1H, *J* = 8.0 Hz, *J* = 1.5 Hz, ArH), 13.75 (s, 1H, OH), 14.01 (s, 1H, OH). ^13^C NMR and DEPT (CDCl_3_, 125 MHz) δ: 165.3 (C), 162.8 (C), 161.2 (C), 161.0 (C), 160.9 (C), 149.7 (C), 146.9 (C), 138.1 (CH), 133.9 (CH), 133.6 (CH), 129.5 (CH), 129.1 (CH), 128.3 (CH), 128.0 (C), 127.0 (CH), 125.4 (CH), 124.0 (CH), 121.8 (CH), 121.6 (CH), 119.4 (CH), 119.3 (CH), 118.9 (CH), 118.2 (CH), 117.6 (C), 117.1 (C), 109.8 (CH). IR (ATR) ν: 2719 (br), 1565, 1484, 1274, 1247, 748, 697, 634 cm^–1^. MALDI-TOF MS (dithranol) *m*/*z*: 534.8 [M + H]^+^. HRMS (ESI) *m*/*z*: calcd for (C_36_H_27_N_3_O_2_) [M]^+^, 533.2098; found, 533.2101 (1 ppm).

### General Procedure for the Synthesis of Boron Complexes **3** and **4**

Reactions were performed in screw-cap glass flasks that were placed inside metal heat transfer blocks. Boron trifluoride diethyl etherate was added to a mixture of the corresponding 2,4-bis(2′-hydroxyphenyl)pyrimidine, cesium carbonate, and chloroform. The reaction mixture was stirred at 80 °C for 6 h. After cooling, it was diluted with dichloromethane and the insoluble cesium carbonate was removed by filtration. The solvent was evaporated under vacuum. Trituration in boiling methanol yielded a solid, which was collected by filtration and then dried under vacuum.

#### Complex **3**

Prepared from 100 mg (0.36 mmol) of 2,4-bis(2′-hydroxyphenyl)-6-methylpyrimidine (**1**), 586 mg (1.8 mmol) of cesium carbonate, and 0.22 mL (1.8 mmol) of boron trifluoride diethyl etherate in 5 mL of chloroform. Light yellowish solid (100 mg, 91%). mp 292–294 °C (dec.). ^1^H NMR (CDCl_3_, 500 MHz) δ: 2.78 (s, 3H, CH_3_), 7.06–7.11 (m, 2H, ArH), 7.20 (dd, 1H, *J* = 8.5 Hz, *J* = 1.0 Hz, ArH), 7.24 (dd, 1H, *J* = 8.5 Hz, *J* = 1.0 Hz, ArH), 7.55–7.59 (m, 2H, ArH), 7.62 (s, 1H, pyr), 7.85 (dd, 1H, *J* = 8.0 Hz, *J* = 1.5 Hz, ArH), 8.45 (dd, 1H, *J* = 8.0 Hz, *J* = 1.0 Hz, ArH). ^13^C NMR and DEPT (CDCl_3_, 125 MHz) δ: 172.2 (C), 157.8 (C), 157.5 (C), 156.1 (C), 153.6 (C), 136.4 (CH), 136.1 (CH), 128.5 (CH), 125.8 (CH), 121.3 (CH), 120.9 (CH), 120.7 (CH), 120.1 (CH), 116.7 (C), 114.7 (C), 111.0 (CH), 25.6 (CH_3_). ^19^F NMR (CDCl_3_, 471 MHz) δ: −135.9 (q, *J* = 39 Hz). ^11^B NMR (CDCl_3_, 160 MHz) δ: 1.55 (d, *J* = 39 Hz). MALDI-TOF MS (dithranol) *m*/*z*: 287.4 [M – 19]^+^. HRMS (ESI) *m*/*z*: calcd for (C_17_H_12_N_2_O_2_F^11^BNa) [M + Na]^+^, 329.0868; found, 329.0872 (1 ppm).

#### Complex **4a**

Prepared from 100 mg (0.25 mmol) of (*E*)-2,4-bis(2′-hydroxyphenyl)-6-(4′-methoxystyryl)pyrimidine (**2a**), 410 mg (1.26 mmol) of cesium carbonate, and 0.2 mL (1.6 mmol) of boron trifluoride diethyl etherate in 5 mL of chloroform. Yellow solid (105 mg, 98%). mp decomposes >240 °C. ^1^H NMR (CDCl_3_, 500 MHz) δ: 3.87 (s, 3H, OCH_3_), 6.97 (A of AB_q_, 2H, *J* = 8.5 Hz, ArH), 7.00–7.04 (m, 2H, CH= and ArH), 7.06–7.09 (m, 1H, ArH), 7.51 (s, 1H, pyr), 7.51–7.56 (m, 2H, ArH), 7.63 (B of AB_q_, 2H, *J* = 8.5 Hz, ArH), 7.80 (dd, 1H, *J* = 8.0 Hz, *J* = 1.0 Hz, ArH), 8.15 (B of AB_q_, 1H, *J* = 16.0 Hz, CH=), 8.50 (dd, 1H, *J* = 8.0 Hz, *J* = 1.5 Hz, ArH). ^13^C NMR and DEPT (CDCl_3_, 125 MHz) δ: 165.6 (C), 161.8 (C), 157.7 (C), 157.3 (C), 155.6 (C), 153.7 (C), 141.9 (CH), 136.0 (CH), 135.7 (CH), 130.2 (CH), 128.3 (CH), 127.6 (C), 125.7 (CH), 121.8 (CH), 121.1 (CH), 120.8 (CH), 120.5 (CH), 120.1 (CH), 117.0 (C), 115.2 (C), 114.6 (CH), 108.8 (CH), 55.5 (OCH_3_). ^19^F NMR (CDCl_3_, 471 MHz) δ: −135.9 (m). ^11^B NMR (CDCl_3_, 160 MHz) δ: 1.50 (d, *J* = 30 Hz). MALDI-TOF MS (dithranol) *m*/*z*: 405.3 [M – 19]^+^. HRMS (ESI) *m*/*z*: calcd for (C_25_H_18_N_2_O_3_F^11^BNa) [M + Na]^+^, 447.1287; found, 447.1291 (1 ppm).

#### Complex **4b**

Prepared from 100 mg (0.22 mmol) of (*E*)-2,4-bis(2′-hydroxyphenyl)-6-[2-(6′-methoxynaphthalen-2-yl)vinyl]pyrimidine (**2b**), 365 mg (1.12 mmol) of cesium carbonate, and 0.2 mL (1.6 mmol) of boron trifluoride diethyl etherate in 5 mL of chloroform. Orange solid (100 mg, 94%). mp decomposes >215 °C. ^1^H NMR (CDCl_3_, 500 MHz) δ: 3.97 (s, 3H, OCH_3_), 7.04–7.07 (m, 1H, ArH), 7.08–7.11 (m, 1H, ArH), 7.16–7.24 (m, 5H, CH= and ArH), 7.53–7.57 (m, 2H, ArH), 7.58 (s, 1H, pyr), 7.78–7.82 (m, 3H, ArH), 7.84 (dd, 1H, *J* = 8.0 Hz, *J* = 1.5 Hz, ArH), 8.02 (s, 1H, ArH), 8.34 (d, 1H, *J* = 15.5 Hz, CH=), 8.55 (dd, 1H, *J* = 8.0 Hz, *J* = 1.5 Hz ArH). ^13^C NMR and DEPT (CDCl_3_, 125 MHz) δ: 165.4 (C), 159.2 (C), 157.8 (C), 157.4 (C), 155.7 (C), 153.9 (C), 142.5 (CH), 136.2 (CH), 135.9 (C), 135.8 (CH), 130.6 (CH), 130.3 (CH), 130.2 (C), 128.8 (C), 128.4 (CH), 127.7 (CH), 125.8 (CH), 124.2 (CH), 123.2 (CH), 121.2 (CH), 120.8 (CH), 120.5 (CH), 120.1 (CH), 119.7 (CH), 117.0 (C), 115.2 (C), 109.1 (CH), 106.1 (CH), 55.4 (OCH_3_). ^19^F NMR (CDCl_3_, 471 MHz) δ: −135.9 (m). ^11^B NMR (CDCl_3_, 160 MHz) δ: 1.53 (d, *J* = 27 Hz). MALDI-TOF MS (dithranol) *m*/*z*: 455.5 [M – 19]^+^. HRMS (ESI) *m*/*z*: calcd for (C_29_H_20_N_2_O_3_F^11^BNa) [M + Na]^+^, 497.1443; found, 497.1445 (0 ppm).

#### Complex **4c**

Prepared from 100 mg (0.27 mmol) of (*E*)-2,4-bis(2′-hydroxyphenyl)-6-styrylpyrimidine (**2c**), 440 mg (1.35 mmol) of cesium carbonate, and 0.2 mL (1.6 mmol) of boron trifluoride diethyl etherate in 5 mL of chloroform. Yellow solid (100 mg, 94%). mp decomposes >270 °C. ^1^H NMR (CDCl_3_, 500 MHz) δ: 7.03–7.07 (m, 1H, ArH), 7.08–7.11 (m, 1H, ArH), 7.19 (A of AB_q_, 1H, *J* = 16.0 Hz, CH=), 7.19–7.23 (m, 2H, ArH), 7.44–7.50 (m, 3H, ArH), 7.53–7.57 (m, 2H, ArH), 7.60 (s, 1H, pyr), 7.69–7.71 (m, 2H, ArH), 7.84 (dd, 1H, *J* = 8.0 Hz, *J* = 1.5 Hz, ArH), 8.22 (B of AB_q_, 1H, *J* = 16.0 Hz, CH=), 8.52 (dd, 1H, *J* = 8.0 Hz, *J* = 1.5 Hz, ArH). ^13^C NMR and DEPT (CDCl_3_, 125 MHz) δ: 165.3 (C), 157.8 (C), 157.4 (C), 155.8 (C), 154.2 (C), 142.1 (CH), 136.3 (CH), 135.9 (CH), 134.8 (C), 130.7 (CH), 129.1 (CH), 128.4 (CH), 128.4 (CH), 125.8 (CH), 124.3 (CH), 121.2 (CH), 120.8 (CH), 120.6 (CH), 120.1 (CH), 116.9 (C), 115.1 (C), 109.3 (CH). ^19^F NMR (CDCl_3_, 471 MHz) δ: −136.1 (q, *J* = 35 Hz). ^11^B NMR (CDCl_3_, 160 MHz) δ: 1.53 (d, *J* = 37 Hz). MALDI-TOF MS (dithranol) *m*/*z*: 375.4 [M – 19]^+^. HRMS (ESI) *m*/*z*: calcd for (C_24_H_16_N_2_O_2_F^11^BNa) [M + Na]^+^, 417.1181; found, 417.1181 (0 ppm).

#### Complex **4d**

Prepared from 50 mg (0.115 mmol) of (*E*)-2,4-bis(2′-hydroxyphenyl)-6-[(4′-trifluoromethyl)styryl]pyrimidine (**2d**), 187 mg (0.575 mmol) of cesium carbonate, and 0.1 mL (0.8 mmol) of boron trifluoride diethyl etherate in 5 mL of chloroform. Yellow solid (50 mg, 94%). mp decomposes >220 °C. ^1^H NMR (CDCl_3_, 500 MHz) δ: 7.01–7.08 (m, 2H, ArH), 7.17–7.25 (m, 3H, ArH and CH=), 7.52–7.57 (m, 2H, ArH), 7.62 (s, 1H, pyr), 7.71 (A of AB_q_, 2H, *J* = 8.5 Hz, ArH), 7.78 (B of AB_q_, 2H, *J* = 8.5 Hz, ArH), 7.80 (d, 1H, *J* = 8.0 Hz, ArH), 8.19 (B of AB_q_, 2H, *J* = 15.5 Hz, CH=), 8.48 (dd, 1H, *J* = 1.5 Hz, *J* = 8.0 Hz, ArH). ^13^C NMR and DEPT (CDCl_3_, 125 MHz) δ: 164.6 (C), 157.8 (C), 157.5 (C), 155.9 (C), 154.5 (C), 140.0 (CH), 138.1 (C), 136.6 (CH), 136.1 (CH), 131.9 (q, *J* = 32.4 Hz, C), 128.4 (CH), 128.3 (CH), 126.6 (CH), 126.0 (q, *J* = 3.8 Hz, CH), 125.8 (CH), 123.8 (q, *J* = 270.7 Hz, CF_3_), 121.2 (CH), 120.9 (CH), 120.6 (CH), 120.1 (CH), 116.7 (C), 114.9 (C), 109.7 (CH). ^19^F NMR (CDCl_3_, 471 MHz) δ: −135.2 (m), −62.8. ^11^B NMR (CDCl_3_, 160 MHz) δ: 1.55 (d, *J* = 27.7 Hz). MALDI-TOF MS (dithranol) *m*/*z*: 443.5 [M – 19]^+^. HRMS (ESI) *m*/*z*: calcd for (C_25_H_15_N_2_O_2_F_4_^11^BNa) [M + Na]^+^, 485.1055; found, 485.1044 (2 ppm).

#### Complex **4e**

Prepared from 100 mg (0.19 mmol) of (*E*)-2,4-bis(2′-hydroxyphenyl)-6-(4′-diphenylaminostyryl)pyrimidine (**2e**), 335 mg (0.95 mmol) of cesium carbonate, and 0.2 mL (1.6 mmol) of boron trifluoride diethyl etherate in 5 mL of chloroform. Red solid (100 mg, 95%). mp decomposes >280 °C. ^1^H NMR (CDCl_3_, 500 MHz) δ: 7.05 (A of AB_q_, 1H, *J* = 15.5 Hz, CH=), 7.05–7.12 (m, 4H, ArH), 7.14–7.24 (m, 8H, ArH), 7.33–7.36 (m, 4H, ArH), 7.54–7.57 (m, 5H, ArH and pyr), 7.87 (dd, 1H, *J* = 8.0 Hz, *J* = 1.5 Hz, ArH), 8.19 (B of AB_q_, 1H, *J* = 15.5 Hz, CH=), 8.54 (dd, 1H, *J* = 8.0 Hz, *J* = 1.5 Hz, ArH). ^13^C NMR and DEPT (CDCl_3_, 125 MHz) δ: 165.7 (C), 157.8 (C), 157.3 (C), 155.6 (C), 153.6 (C), 150.4 (C), 146.7 (C), 142.0 (CH), 136.0 (CH), 135.7 (CH), 129.8 (CH), 129.6 (CH), 128.3 (CH), 127.7 (C), 125.7 (CH), 125.7 (CH), 124.4 (CH), 121.4 (CH), 121.3 (CH), 121.2 (CH), 120.7 (CH), 120.5 (CH), 120.1 (CH), 117.1 (C), 115.3 (C), 108.8 (CH). ^19^F NMR (CDCl_3_, 471 MHz) δ: −136.5 (m). ^11^B NMR (CDCl_3_, 160 MHz) δ: 1.50 (d, *J* = 25.3 Hz). MALDI-TOF MS (dithranol) *m*/*z*: 562.9 (M + H), 542.8 [M – 19]^+^. HRMS (ESI) *m*/*z*: calcd for (C_36_H_25_N_3_O_2_F^11^BNa) [M + Na]^+^, 584.1916; found, 584.1921 (1 ppm).

## Conclusions

A series of 6-arylvinyl-2,4-bis(2′-hydroxyphenyl)pyrimidines was readily synthesized through the combination of Suzuki–Miyaura cross-coupling and acid-catalyzed Knoevenagel condensation reactions. None of these compounds exhibited luminescence, which was attributed to a ESIPT process from the OH groups to the nitrogen atoms of the pyrimidine ring. As observed in analogous structures, protonation inhibited the ESIPT process, resulting in a significant increase in the fluorescence response. The compounds demonstrated their capacity to act as rigid tridentate chelating ligands through the successful preparation of four-coordinate organoboron compounds, achieving excellent yields. The enhanced structural planarity and increased rigidity into the framework of these boron complexes led to intense luminescence in both solution and the solid state, allowing for effective fine-tuning of the emission color by modifying the substituent on the arylvinylene moiety. The presence of donor and acceptor groups coupled by a π-conjugated spacer enables ICT processes that lead to highly polarized singlet excited states, as reflected by the significant red-shifts observed in the emission maxima upon increasing the solvent polarity. Comprehensive tools such as X-ray diffraction analysis, DFT, and TD-DFT calculations were pivotal in interpreting all the experimental results.

## Supporting Information Available

Additional experimental details, material, and methods; crystallographic and refinement data for compounds **1**, **2a**, and **3**; geometrical features of the intermolecular interactions (**1**, **2a**, and **3**); dipole moments for the ground state (S_0_) and the first excited state (S_1_), values of energies and relative energies for the tautomers, and theoretical emission wavelengths for the S_1_ → S_0_ transition (**1**, **2a**, **3**, **4a**–**b**, and **4e**); wavenumber, reorganization energy, and Huang–Rhys factor (**1**, **2a**, **3**, **4a**–**b**, and **4e**); values of energy and relative energies for the tautomers of protonated compound **2aH**^**+**^; wavenumber, reorganization energy, and Huang–Rhys factor for the protonated compound **2aH**^**+**^; NMR, IR, and MALDI-TOF MS spectra; absorption and emission spectra before and after the addition of TFA (**2a**–**e**); absorption and emission spectra in solution and in the solid state (**3** and **4a**–**e**); emission spectra and photographs of **3** and **4d**–**e** in various solvents; selected bond lengths and dihedral angles in the S_0_ and S_1_ states (**1**, **2a**, **3**, **4a**–**b**, and **4e**); molecular orbitals calculated for the ground and excited states (**4a** and **4b**); Huang–Rhys factors calculated for the ground state of compounds **3**, **4a**, **4b**, and **4e**; selected bond lengths and dihedral angles for compounds **2aH**^**+**^**(N1)** and **2aH**^**+**^**(N3)** in the S_0_ and S_1_ states; molecular orbitals calculated for **2aH**^**+**^**(N1)** and **2aH**^**+**^**(N3)** in the ground and excited states (PDF)
