# Supplementary material for: Tunable Emission and Structural Insights of 6-Arylvinyl-2,4-bis(2′-hydroxyphenyl)pyrimidines and Their O∧N∧O-Chelated Boron Complexes
Source: ACS Appl Opt Mater. 2024 Sep 20;2(10):2051–66. doi: 10.1021/acsaom.4c00251 (PMC11524413; doi:10.1021/acsaom.4c00251)
Supplement: Supplementary file 1 — ot4c00251_si_001.pdf [file ot4c00251_si_001.pdf]

SUPPORTING INFORMATION

Tunable Emission and Structural Insights of 6-Arylvinyl-2,4-bis(2′-hydroxyphenyl)pyrimidines and Their O^N^O-Chelated Boron Complexes

Rodrigo Plaza-Pedroche,[a] *M. Paz Fernández-Liencres,*[b] *Sonia B. Jiménez-Pulido,*[c] *Nuria A. Illán-Cabeza,*[c] *Sylvain Achelle,*[d] *Amparo Navarro,*[b,]* *and Julián Rodríguez-López.*[a,]*

[a] Universidad de Castilla-La Mancha, Área de Química Orgánica, Facultad de Ciencias y Tecnologías Químicas, Avda. Camilo José Cela 10, 13071 Ciudad Real, Spain. E-mail: julian.rodriguez@uclm.es

[b] Universidad de Jaén, Dpto. de Química Física y Analítica, Facultad de Ciencias Experimentales, Campus Las Lagunillas, 23071 Jaén (Spain). E-mail: anavarro@ujaen.es

[c] Universidad de Jaén, Dpto. de Química Inorgánica y Orgánica, Facultad de Ciencias Experimentales, Campus Las Lagunillas, 23071 Jaén (Spain).

[d] Univ. Rennes, CNRS, Institut des Sciences Chimiques de Rennes (ISCR), UMR 6226, F-35000 Rennes, France.

General information. All reagents obtained from commercial sources were used as received. All solvents were reagent grade for synthesis and spectroscopic grade for photophysical measurements. NMR spectra were recorded at room temperature on a Bruker Avance Neo 500 spectrometer. The chemical shifts (δ) are reported in ppm and are referenced internally to the solvent signals of $CDCl_3$ (^1H, 7.27 ppm; ^{13}C, 77.0 ppm) or DMSO-d_6 (^1H, 2.50 ppm; ^{13}C, 39.5 ppm), and externally to $CFCl_3$ (^{19}F, 0.0 ppm) and to $BF_3.Et_2O$ (^{11}B, 0.0 ppm). The coupling constants *J* are given in Hz. In the ^1H NMR spectra, the following abbreviations are used to describe the peak patterns: s (singlet), d (doublet), t (triplet), q (quartet), and m (multiplet). In the ^{13}C NMR spectra, the nature of the carbons (C, CH, CH_2 or CH_3) was determined by performing a DEPT experiment. Acidic impurities in $CDCl_3$ were removed by treatment with solid K_2CO_3. The IR spectra were recorded with a Jasco FT/IR-4700 spectrophotometer equipped with an attenuated total reflectance (ATR) accessory. MALDI-TOF mass spectra were obtained on a Bruker Autoflex II spectrometer in positive detection mode, using dithranol as matrix. High Resolution Mass Spectrometry (HRMS) analyses were performed at the “Centre Régional de Mesures Physiques de l'Ouest” (CRMPO, University of Rennes 1, France) using a Bruker MicroTOFQ II apparatus. UV-visible and fluorescence spectroscopy studies were conducted on a Jasco V-750 spectrophotometer and Jasco FP-8300 spectrofluorometer, respectively. Compounds were excited at their absorption maxima (band of lowest energy) to record the emission spectra. All solutions were measured with optical densities below 0.1.

Computational details. Full geometry optimization of the ground state and the first excited state was performed using the Gaussian16 (revision A.03) suite of programs.[1] The PBE0,[2] M06-2X,[3] CAM-B3LYP,[4] and ωB97x-D[5] functionals were chosen along with the 6-31+G** and 6-31G** basis sets The vibrational modes were calculated to check the absence of imaginary frequencies in S_0 and S_1. The solvent environment was described by the polarizable continuum model (PCM) as implemented in the Gaussian package.[6] The relaxed potential energy scan (PES) was computed in solution, constraining the oxygen···hydrogen bond length and optimizing all other coordinates at each scan point. The vertical electronic transitions (absorption and emission) were

(1) Frisch, M. J.; Trucks, G. W.; Schlegel, H. B.; Scuseria, G. E.; Robb, M. A.; Cheeseman, J. R.; Scalmani, G.; Barone, V.; Petersson, G. A.; Nakatsuji, H.; Li, X.; Caricato, M.; Marenich, A. V.; Bloino, J.; Janesko, B. G.; Gomperts, R.; Mennucci, B.; Hratchian, H. P.; Ortiz, J. V.; Izmaylov, A. F.; Sonnenberg, J. L.; Williams-Young, D.; Ding, F.; Lipparini, F.; Egidi, F.; Goings, J.; Peng, B.; Petrone, A.; Henderson, T.; Ranasinghe, D.; Zakrzewski, V. G.; Gao, J.; Rega, N.; Zheng, G.; Liang, W.; Hada, M.; Ehara, M.; Toyota, K.; Fukuda, R.; Hasegawa, J.; Ishida, M.; Nakajima, T.; Honda, Y.; Kitao, O.; Nakai, H.; Vreven, T.; Throssell, K.; Montgomery, Jr., J. A.; Peralta, J. E.; Ogliaro, F.; Bearpark, M. J.; Heyd, J. J.; Brothers, E. N.; Kudin, K. N.; Staroverov, V. N.; Keith, T. A.; Kobayashi, R.; Normand, J.; Raghavachari, K.; Rendell, A. P.; Burant, J. C.; Iyengar, S. S.; Tomasi, J.; Cossi, M.; Millam, J. M.; Klene, M.; Adamo, C.; Cammi, R.; Ochterski, J. W.; Martin, R. L.; Morokuma, K.; Farkas, O.; Foresman, J. B.; Fox, D. J. Gaussian 16, Revision A.03. Gaussian, Inc., Wallingford CT, 2016.

(2) Ernzerhof, M.; Perdew, J. P. Generalized Gradient Approximation to the Angle- and System-Averaged Exchange Hole. *J. Chem. Phys.* **1998**, *109*, 3313–3320. https://doi.org/10.1063/1.476928

(3) Zhao, Y.; Truhlar, D. G. The M06 Suite of Density Functionals for Main Group Thermochemistry, Thermochemical Kinetics, Noncovalent Interactions, Excited States, and Transition Elements: Two New Functionals and Systematic Testing of Four M06-Class Functionals and 12 Other Functionals. *Theor. Chem. Acc.* **2008**, 120, 215–241. https://doi.org/10.1007/s00214-007-0310-x

(4) Yanai T.; Tew, D. P.; Handy, N. C. A New Hybrid Exchange–Correlation Functional Using the Coulomb-Attenuating Method (CAM-B3LYP). *Chem. Phys. Lett.* **2004**, 393, 51–57. https://doi.org/10.1016/j.cplett.2004.06.011

(5) Chai, J.-D.; Head-Gordon, M. Systematic Optimization of Long-Range Corrected Hybrid Density Functionals. *J. Chem. Phys.* **2008**, *128*, 084106. https://doi.org/10.1063/1.2834918

(6) (a) Cossi, M.; Rega, N.; Scalmani, G.; Barone, V. Energies, Structures, and Electronic Properties of Molecules in Solution with the C-PCM Solvation Model. *J. Comput. Chem.* **2003**, *24*, 669–681. https://doi.org/10.1002/jcc.10189 (b) Tomasi, J.; Mennucci, B.; Cammi, R. Quantum Mechanical Continuum Solvation Models. *Chem. Rev.* **2005**, *105*, 2999–3094. https://doi.org/10.1021/cr9904009 (c) Cammi, R.; Corni, S.; Mennucci, B.; Tomasi, J. Electronic Excitation Energies of Molecules in Solution: State Specific and Linear Response Methods for Nonequilibrium Continuum Solvation Models. *J. Chem. Phys.* **2005**, *122*, 104513. https://doi.org/10.1063/1.1867373

computed using time dependent (TD)-DFT calculations. The vertical electronic transitions for $S_1 \rightarrow S_0$ were calculated in solution as $\Delta E_{em} = E_{S1}(G_{S1}) - E_{S0}(G_{S1})$, where $E_{S1}(G_{S1})$ is the energy of the S_1 state at its equilibrium geometry (state-specific solvation approach, SS)[7] and $E_{S0}(G_{S1})$ is the energy of the S_0 state at the S_1 state geometry with the static solvation from the excited state.[8] The $E_{S1}(G_{S1})$ energy was also calculated using linear response (LR) and corrected linear response (cLR) approaches.[9] The reorganization energy, λ_i, and Huang-Rhys (HR) factors for each vibrational mode, S_i, were calculated to account for non-radiative vibrational relaxation using the DUSHIN program,[10] which are related as follows: $\lambda = \sum_i \lambda_i = \sum_i \hbar\, \omega_i S_i$, where ω_i is the wavenumber associated to the vibrational mode i.
The ONIOM approach[11] was used to mimic the solid state. A model cluster was constructed based on the X-ray crystal structure, with one central molecule treated at a high level using M06-2X/6-31G**, and both S_0 and S_1 electronic state geometries were fully optimized. Surrounding molecules, treated at a low level, were subjected to molecular mechanics (MM) using the UFF[12] force field with their molecular geometries frozen. TD-DFT calculations were performed for the central molecule to predict the vertical electronic transitions $S_1 \rightarrow S_0$ from the excited state. Polarization effects were taken into account using the mechanical embedding (ME) approach, which is the method implemented by default in Gaussian16.[13]

Crystallography. Data processing and refinement parameters of compounds **1**, **2a**, and **3** are provided in Table S1. X-ray data were collected at 100 K on a Bruker-Apex-II CCD diffractometer equipped with graphite monochromated Mo-K_α (λ= 0.71073 Å) radiation. The raw data were integrated and corrected for Lorentz and polarization effects with the aid of the Bruker APEX II program suite. Absorption corrections were applied with SADABS.[14] The structures were solved through conventional direct methods and refined against F^2 using the full-matrix least-squares methods with the SHELXL-2016/4 program,[15] integrated into the WinGX program package (version 2021.3).[16] All non-H atoms were refined anisotropically, while hydrogen atoms were

---

(7) Improta, R.; Barone, V.; Scalmani, G.; Frisch, M. J. A State-Specific Polarizable Continuum Model Time Dependent Density Functional Theory Method for Excited State Calculations in Solution. *J. Chem. Phys.* **2006**, *125*, 054103. https://doi.org/10.1063/1.2222364

(8) Scalmani, G.; Frisch, M. J.; Mennucci, B.; Tomasi, J.; Cammi, R.; Barone, V. Geometries and Properties of Excited States in the Gas Phase and in Solution: Theory and Application of a Time-Dependent Density Functional Theory Polarizable Continuum Model. *J. Chem. Phys.* **2006**, *124*, 094107. https://doi.org/10.1063/1.2173258

(9) Caricato, M.; Mennucci, B.; Tomasi, J.; Ingrosso, F.; Cammi, R.; Corni, S.; Scalmani, G. Formation and Relaxation of Excited States in Solution: A New Time Dependent Polarizable Continuum Model Based on Time Dependent Density Functional Theory. *J. Chem. Phys.* **2006**, *124*, 124520. https://doi.org/10.1063/1.2183309

(10) Reimers, J. R. A Practical Method for the Use of Curvilinear Coordinates in Calculations of Normal-Mode-Projected Displacements and Duschinsky Rotation Matrices for Large Molecules. *J. Chem. Phys*. **2001**, *115*, 9103-9109. https://doi.org/10.1063/1.1412875

(11) (a) Dapprich, S.; Komáromi, I.; Byun, K. S.; Morokuma, K.; Frisch, M. J. A New ONIOM Implementation in Gaussian98. Part I. The Calculation of Energies, Gradients, Vibrational Frequencies and Electric Field Derivatives. *J. Mol. Struct. (Theochem)* **1999**, *461-462*, 1–21. https://doi.org/10.1016/S0166-1280(98)00475-8 (b) Vreven, T.; Morokuma, K.; Farkas, O.; Schlegel, H. B.; Frisch, M. J. Geometry Optimization with QM/MM, ONIOM, and Other Combined Methods. I. Microiterations and Constraints. *J. Comput. Chem.* **2003**, *24*, 760–769. https://doi.org/10.1002/jcc.10156 (c) Lin, H.; Truhlar, D. G. QM/MM: What Have We Learned, Where Are We, and Where Do We Go from Here?. *Theor. Chem. Acc*. **2007**, *117*, 185–199. https://doi.org/10.1007/s00214-006-0143-z

(12) Casewit, C. J.; Colwell, K. S.; Rappe, A. K. Application of a Universal Force Field to Organic Molecules. *J. Am. Chem. Soc.* **1992**, *114*, 10035–10046. https://doi.org/10.1021/ja00051a041

(13) Vreven, T.; Byun, K. S.; Komáromi, I.; Dapprich, S.; Montgomery, Jr., J. A.; Morokuma, K.; Frisch. M. J. Combining quantum mechanics methods with molecular mechanics methods in ONIOM. *J. Chem. Theory Comput.* **2006**, *2*, 815–826. https://doi.org/10.1021/ct050289g

(14) SADABS, version 2016/2, Bruker AXS Inc., Madison, WI, USA, 2016.

(15) Sheldrick, G. M. SHELXL-2016/4. University of Göttingen, Göttingen, Germany, 2016.

(16) Farrugia, L. J. WinGX and ORTEP for Windows: An Update. *J. Appl. Cryst.* **2012**, *45*, 849−854. http://dx.doi.org/10.1107/S0021889812029111

located and refined isotropically, except in compound **1**, where specific hydrogen atoms were placed in idealized positions and treated using riding models. The structural models were analyzed using PLATON,[17] and graphics were generated using MERCURY software.[18] CIF files have been deposited at the Cambridge Crystallographic Data Center with CCDC numbers: 2329831 (**1**), 2329821 (**2a**), and 2329830 (**3**).

Table S1. Crystallographic and refinement data for **1**, **2a**, and **3**.

**Compound**	**1**	**2a**	**3**
CCDC number	2329831	2329821	2329830
Formula	$C_{17}H_{14}N_2O_2$	$C_{25}H_{20}N_2O_3$	$C_{17}H_{12}BFN_2O_2$
FW (g·mol^{-1})	278.30	396.43	306.10
Color, habit	yellow, block	yellow, block	Colorless, block
Crystal size (mm^3)	0.100x0.090x0.090	0.130x0.095x0.040	0.140x0.060x0.050
Crystal system	Monoclinic	Orthorhombic	Triclinic
Space group	*P 21/c*	*P 21 21 21*	*P -1*
Unit cell dimens. *a* (Å)	10.329(1)	6.432(1)	7.424(1)
b (Å)	8.907(1)	12.003(1)	8.692(1)
c (Å)	14.161(1)	25.397(1)	10.648(1)
α (°)	90	90	100.75(1)
β (°)	96.93(1)	90	91.394(1)
γ (°)	90	90	98.28(1)
Volume (Å^3)	96.93(1)	1960.6(2)	667.1(1)
Z	4	4	2
Density (calc. Mg·m^{-3})	1.429	1.343	1.524
μ (mm^{-1})	0.095	0.089	0.110
F(000)	584	832	316
θ range (°)	2.71- 27.11	2.33-27.11	1.95-27.15
Index ranges	$-13 \le h \le 13$	$-8 \le h \le 8$	$-9 \le h \le 9$
	$-11 \le k \le 11$	$-15 \le k \le 15$	$-11 \le k \le 11$
	$-18 \le l \le 18$	$-30 \le l \le 32$	$-13 \le l \le 13$
Reflecs. collected	2856	4331	2958
Indep./ I>2σ(I)	2318	4014	2502
R_{int}	0.058	0.056	0.059
Weighting scheme $w^{-1} = \sigma^2(Fo^2) + (xP)^2 + yP$ $(P=(Fo^2+2Fc^2)/3)$			
x/y	0.0523/0.5869	0.0374/0.5491	0.0701/0.0508
Data /restraints /parameters	2856 / 0 / 235	4331 / 0 / 288	2958 / 0 / 256
Goodness-of-fit on F^2	1.049	1.045	1.096
R1/*w*R2 [I >2σ(I)]	0.0412/0.1127	0.0338/0.0798	0.0378/ 0.1105
R1/*w*R2 (all data)	0.0541/ 0.1035	0.0381/0.0823	0.0488/ 0.1241
Largest Δρ (e·Å^{-3})	0.047/-0.216	0.183/-0.187	0.383/-0.353

(17) Spek, A. L. PLATON. A Multipurpose Crystallographic Tool. Utrecht University, Utrecht, The Netherlands, 2002.
(18) Macrae, C. F.; Sovago, I.; Cottrell, S. J.; Galek, P. T. A.; McCabe, P.; Pidcock, E.; Platings, M.; Shields, G. P.; Stevens, J. S.; Towler, M.; Wood, P. A. Mercury 4.0: From Visualization to Analysis, Design and Prediction. *J. Appl. Cryst.* **2020**, *53*, 226−235. https://doi.org/10.1107/S1600576719014092

Table S2. Geometrical features of the intermolecular interactions.

**Compound 1**	**d(Cg-Cg) (Å)**[a]	**α (º)**[b]	**β (º)**[c]	**γ (º)**[c]	**Slippage (Å)**
Cg1 – CgA (-x, 1-y, 1-z)	3.736 (8)	8.44 (7)	27.7	24.0	1.739
Cg1 – CgB (1-x, 1-y, 1-z)	3.6215 (8)	1.19 (6)	22.9	22.4	1.408
CgA – Cg1 (-x, 1-y, 1-z)	3.736 (8)	8.44 (7)	24.0	27.7	1.520
CgB – Cg1 (1-x, 1-y, 1-z)	3.6215 (8)	1.19 (6)	22.4	22.9	1.379
CgA – Cg1 (1-x, 2-y, 1-z)	3.7085 (8)	0.00 (7)	26.2	26.2	1.639
**Compound 2a**					
Cg1 – CgB (1+x, y, z)	3.5578 (11)	0.11 (9)	21.1	21.2	1.283
CgB – Cg1 (-1+x, y, z)	3.5578 (11)	0.11 (9)	21.2	21.1	1.287
**Compound 3**					
CgB – CgA (1-x, 2-y, 1-z)	3.5876 (9)	9.27 (7)	18.5	15.8	1.139
CgA – CgB (1-x, 2-y, 1-z)	3.5877 (9)	9.27 (7)	15.8	18.5	0.977
Cg1 – CgB (1-x, 1-y, 1-z)	3.8312 (9)	12.72 (6)	20.3	28.5	1.329
CgB – Cg1 (1-x, 1-y, 1-z)	3.8313 (9)	12.72 (6)	28.5	20.3	1.829

Cg1 is the centroid of the pyrimidine ring.

CgA and CgB are the centroids of the benzene rings, designated as A and B in Figure 1.

[a] Distance between centroids of rings involved in π-interactions (only those shorter than 4 Å are given).

[b] Angle between planes of both centroids.

[c] Slipping angles between the centroid-centroid vector and the normal to each stacked ring plane.

Table S3. Dipole moments (in Debye) for the ground state (S_0) and the first excited state (S_1) calculated at the M06-2X/6-31+G** and PBE0/6-31+G** levels of theory in CH_2Cl_2 solution.

Compound		M06-2X	PBE0
**1**	S_0	5.06	5.00
	S_1 (EK)	7.39	11.62
	S_1 (KE)	12.67	13.25
**2a**	S_0	6.04	6.78
	S_1 (EE)	8.98	8.82
	S_1 (EK)	12.21	17.78
	S_1 (KE)	12.86	13.66
**3**	S_0	7.68	7.49
	S_1	7.66	9.05
**4a**	S_0	10.50	11.31
	S_1	13.90	13.21
**4b**	S_0	10.52	11.43
	S_1	14.36	13.81
**4e**	S_0	11.63	12.96
	S_1	15.58	12.68

Table S4. Values of energy (in Hartree) and relative energies (ΔE in kcal/mol) for the tautomers of compounds in CH_2Cl_2, calculated at the PBE0/6-31+G** and M06-2X/6-31+G** levels of theory.

Compd	State	Energy	ΔE	Energy	ΔE
		PBE0		M062X	
**1**	S_0 (*EE*)	-915.252592		-915.914522	
	S_1 (*EK*)	-915.151927	3.13	-915.791004	1.88
	S_1 (*KE*)	-915.156915	**0.00**	-915.793994	**0.00**
**2a**	S_0 (*EE*)	-1298.500974		-1299.445308	
	S_1 (*EE*)	-1298.394865	13.90	-1299.329697	2.20
	S_1 (*EK*)	-1298.412361	2.93	-1299.332909	0.18
	S_1 (*KE*)	-1298.417026	**0.00**	-1299.333197	**0.00**
**3**	S_0	-1038.795834		-1039.557836	
	S_1	-1038.681779		-1039.427124	
**4a**	S_0	-1422.046347		-1423.090167	
	S_1	-1421.944541		-1422.979717	
**4b**	S_0	-1575.518950		-1576.678018	
	S_1	-1575.424198		-1576.571990	
**4e**	S_0	-1824.518189		-1825.874216	
	S_1	-1824.436440		-1825.778778	

KE: proton transferred to N1; *EK*: proton transferred to N3.

Table S5. Values of energy (in Hartree) and relative energies (ΔE in kcal/mol) for the tautomers of compounds in CH_2Cl_2, calculated at the CAM-B3LYP/6-31+G** and ωB97x-D/6-31+G** levels of theory.

Compound	State	Energy	ΔE	Energy	ΔE
		CAM-B3LYP		ωB97X-D	
**1**	S_0 (*EE*)	-915.826881			
	S_1 (*EK*)	-915.704576	0.77	-915.866379	**0.00**
	S_1 (*KE*)	-915.705799	**0.00**	-915.865786	0.37
	S_1 (*KK*)	-915.696337	5.94	-915.858228	5.11
**2a**	S_0 (*EE*)	-1299.314834		-1299.549201	
	S_1 (*EE*)	-1299.198880	2.70	-1299.431574	2.20
	S_1 (*EK*)	-1299.203176	**0.00**	-1299.435083	**0.00**
	S_1 (*KE*)	-1299.201792	0.87	-1299.431557	2.21
	S_1 (*KK*)	-1299.196290	4.32	-1299.428022	4.43

KE: proton transferred to N1; *EK*: proton transferred to N3.

Table S6. Theoretical emission wavelengths ($\lambda_{vert\text{-}em}^{calc}$) for the $S_1 \rightarrow S_0$ transition in CH_2Cl_2 solution calculated at the TD-DFT/6-31+G** levels of theory.

**Compd**		**λ_{em}^{calc} nm (eV)**	***f***	**% Contr.**	**λ_{em}^{calc} nm (eV)**	***f***	**% Contr.**
		**PBE0 (SS)**			**PBE0 (cLR)**		
**1**	*KE*	1038 (1.20)	0.05	H → L (99)	1415 (0.88)	0.05	H → L (99)
	EK		0.00	H → L (97)	52760 (0.02)	0.00	H → L (97)
**2a**	*KE*	1479 (0.84)	0.02	H →L (100)	1174 (0.70)	0.02	H → L (100)
	EK		0.00	H → L (99)	-3709 (-0.33)	0.00	H → L (99)
	EE	484 (2.56)	1.54	H → L (97)	428 (2.20)	1.54	H → L (97)
**3**		901 (1.38)	0.04	H → L (98)	641 (1.94)	0.04	H → L (98)
**4a**		502 (2.47)	1.60	H → L (97)	443 (2.80)	1.60	H → L (97)
**4b**		619 (2.00)	1.76	H → L (97)	506 (2.45)	1.76	H → L (97)
**4e**		829 (1.50)	1.19	H → L (97)	707 (1.75)	1.19	H → L (97)
		**M062X (LR)**			**M062X (cLR)**		
**1**	*KE*	539 (2.30)	0.18	H → L (94)	715 (1.74)	0.18	H → L (94)
	EK	-1229 (-1.01)	0.27	H → L (90)	581 (2.14)	0.27	H → L (90)
**2a**	*KE*	565 (2.20)	0.10	H → L (96)	846 (1.47)	0.10	H → L (96)
	EK	546 (2.37)	0.70	H → L (92)	609 (2.04)	0.70	H → L (92)
	EE	415 (2.99)	1.64	H → L (96)	408 (3.04)	1.64	H → L (96)
**3**		367 (3.38)	0.44	H → L (87)	369 (3.36)	0.44	H → L (87)
**4a**		428 (2.90)	1.75	H → L (95)	421 (2.94)	1.75	H → L (95)
**4b**		450 (2.76)	2.07	H → L (93)	453 (2.74)	2.07	H → L (93)
**4e**		496 (2.50)	1.90	H → L (90)	511 (2.43)	1.90	H → L (90)

Table S7. Wavenumber (ν_i in cm^{-1}), reorganization energy (λ_i in meV), and Huang-Rhys factor (dimensionless) calculated for the studied compounds in CH_2Cl_2 solution at the M062X/6-31+G** level of the theory.

**1-*EK***			**2a-*KE***			**3**			**4a**			**4b**			**4e**		
ν_i	λ_i	HR	ν_i	λ_i	HR	ν_i	λ_i	HR	ν_i	λ_i	HR	ν_i	λ_i	HR	ν_i	λ_i	HR
23	68	23.83	22	1	0.37	40	35	7.05	34	1	0.24	24	1	0.34	19	2	0.85
32	23	5.79	27	1	0.30	95	2	0.17	39	1	0.21	108	2	0.15	38	1	0.21
70	3	0.35	30	1	0.27	154	1	0.05	126	5	0.32	188	1	0.04	65	1	0.12
81	4	0.40	98	1	0.08	164	3	0.15	190	2	0.08	307	2	0.05	89	2	0.18
83	33	3.20	109	2	0.15	207	2	0.08	306	1	0.03	347	1	0.02	115	4	0.28
101	4	0.32	129	1	0.06	259	1	0.03	376	2	0.04	375	3	0.06	185	2	0.09
169	2	0.10	163	3	0.15	276	5	0.15	387	1	0.02	528	3	0.05	228	1	0.04
184	41	1.80	248	5	0.16	307	3	0.08	456	1	0.02	542	3	0.04	288	2	0.06
195	3	0.12	271	1	0.03	314	2	0.05	530	1	0.02	556	1	0.01	301	1	0.03
219	1	0.04	273	2	0.06	363	9	0.20	542	2	0.03	577	2	0.03	308	4	0.10
261	15	0.46	358	1	0.02	375	3	0.06	582	2	0.03	610	1	0.01	358	1	0.02
271	4	0.12	385	2	0.04	431	2	0.04	625	2	0.03	642	1	0.01	372	1	0.02
292	5	0.14	444	1	0.02	518	1	0.02	638	1	0.01	705	1	0.01	425	1	0.02
350	6	0.14	467	3	0.05	541	1	0.01	647	1	0.01	753	1	0.01	576	1	0.01
394	3	0.06	491	5	0.08	544	2	0.03	727	1	0.01	945	3	0.03	642	1	0.01
443	5	0.09	507	30	0.48	563	7	0.10	729	1	0.01	983	1	0.01	751	4	0.04
459	11	0.19	566	6	0.09	609	4	0.05	802	1	0.01	1084	6	0.04	770	1	0.01
468	2	0.03	591	5	0.07	611	1	0.01	875	1	0.01	1126	2	0.01	846	1	0.01
502	4	0.06	644	3	0.04	630	1	0.01	885	1	0.01	1138	3	0.02	872	1	0.01
538	4	0.06	648	3	0.04	667	1	0.01	886	1	0.01	1146	2	0.01	927	1	0.01
543	1	0.01	664	1	0.01	698	2	0.02	945	3	0.03	1158	4	0.03	945	3	0.03
548	5	0.07	667	1	0.01	744	1	0.01	1084	7	0.05	1167	2	0.01	959	1	0.01
580	1	0.01	691	8	0.09	780	1	0.01	1108	1	0.01	1187	3	0.02	1014	1	0.01
603	2	0.03	798	3	0.03	823	6	0.06	1126	2	0.01	1202	16	0.11	1084	6	0.04
646	2	0.02	827	4	0.04	834	1	0.01	1138	4	0.03	1210	4	0.03	1126	1	0.01
668	23	0.28	843	1	0.01	886	5	0.05	1145	1	0.01	1232	1	0.01	1137	2	0.01
681	9	0.11	850	5	0.05	893	2	0.02	1146	1	0.01	1277	1	0.01	1145	2	0.01
761	8	0.08	879	3	0.03	897	3	0.03	1159	4	0.03	1284	3	0.02	1157	5	0.03
773	2	0.02	1042	1	0.01	934	2	0.02	1167	1	0.01	1324	2	0.01	1167	2	0.01
780	1	0.01	1130	1	0.01	963	1	0.01	1200	18	0.12	1325	1	0.01	1193	3	0.02
805	10	0.10	1155	2	0.01	1086	6	0.04	1211	2	0.01	1347	12	0.07	1199	15	0.10
818	31	0.31	1166	9	0.06	1112	11	0.08	1244	1	0.01	1384	2	0.01	1204	3	0.02
831	21	0.20	1201	2	0.01	1125	18	0.13	1278	3	0.02	1432	8	0.05	1214	2	0.01
848	17	0.16	1210	8	0.05	1136	12	0.09	1303	1	0.01	1455	2	0.01	1277	3	0.02
853	1	0.01	1214	1	0.01	1145	6	0.04	1317	3	0.02	1480	10	0.05	1326	2	0.01
878	1	0.01	1245	1	0.01	1238	4	0.03	1323	1	0.01	1487	2	0.01	1348	7	0.04
880	5	0.05	1259	1	0.01	1283	1	0.01	1325	1	0.01	1495	4	0.02	1350	3	0.02
884	1	0.01	1266	10	0.06	1308	1	0.01	1328	1	0.01	1507	1	0.01	1353	1	0.01
886	2	0.02	1284	12	0.08	1321	1	0.01	1352	16	0.10	1531	2	0.01	1356	1	0.01
942	12	0.10	1295	9	0.06	1363	12	0.07	1370	2	0.01	1533	3	0.02	1367	2	0.01
1023	1	0.01	1314	2	0.01	1374	8	0.05	1376	2	0.01	1538	2	0.01	1370	2	0.01
1073	1	0.01	1324	5	0.03	1400	2	0.01	1449	1	0.01	1545	1	0.01	1374	1	0.01
1155	1	0.01	1350	27	0.16	1420	10	0.06	1475	7	0.04	1567	1	0.01	1448	1	0.01
1167	2	0.01	1352	1	0.01	1440	3	0.02	1496	5	0.03	1593	24	0.12	1476	3	0.02
1180	1	0.01	1357	20	0.12	1478	2	0.01	1507	1	0.01	1640	25	0.12	1495	5	0.03
1198	6	0.04	1416	7	0.04	1497	1	0.01	1531	3	0.02	1650	6	0.03	1530	2	0.01
1224	1	0.01	1429	41	0.23	1509	4	0.02	1535	1	0.01	1661	25	0.12	1538	1	0.01
1257	1	0.01	1443	125	0.70	1532	10	0.05	1545	2	0.01	1694	11	0.05	1544	2	0.01
1278	1	0.01	1461	10	0.06	1535	19	0.10	1567	2	0.01	1708	42	0.20	1558	2	0.01
1289	5	0.03	1471	23	0.13	1608	22	0.11	1593	28	0.14	1724	18	0.08	1591	21	0.11
1296	4	0.02	1517	24	0.13	1647	4	0.02	1637	35	0.17				1628	21	0.10
1351	47	0.28	1526	23	0.12				1651	1	0.00				1636	19	0.09
1358	7	0.04	1553	1	0.01				1661	18	0.09				1648	4	0.02
1398	8	0.05	1555	6	0.03				1687	17	0.08				1655	12	0.06
1415	5	0.03	1603	86	0.43				1689	1	0.00				1688	5	0.02
1429	59	0.33	1639	7	0.03				1723	36	0.17				1689	8	0.04
1440	3	0.02	1662	1	0.00										1720	13	0.06
1455	66	0.37	1665	54	0.26												
1465	27	0.15	1686	2	0.01												
1493	6	0.03	1698	17	0.08												
1519	80	0.42	1698	12	0.06												
1528	4	0.02	1727	2	0.01												
1556	21	0.11	3193	10751	27.14												
1620	17	0.08	3200	61	0.15												
1648	2	0.01	3207	1013	2.55												
1666	5	0.02	3212	3	0.01												
1669	26	0.13	3216	108	0.27												
1699	10	0.05	3220	86	0.22												

Table S7 (continued).

1-*EK*			2a-*KE*			3			4a			4b			4e		
v_i	λ_i	HR	v_i	λ_i	HR	v_i	λ_i	HR	v_i	λ_i	HR	v_i	λ_i	HR	v_i	λ_i	HR
3183	281	0.71	3222	14	0.04												
3189	141	0.36	3226	10	0.02												
3213	14011	35.15	3234	1	0.00												
3220	22	0.06	3235	2	0.00												
3222	763	1.91	3238	1	0.00												
3235	9	0.02	3239	3	0.01												
3241	11	0.03	3251	3	0.01												
3249	7	0.02	3273	6	0.01												
3254	7	0.02															
3278	14	0.03															

Table S8. Values of energy (in Hartree) and relative energies (ΔE in kcal/mol) for the tautomers of protonated compound **2aH$^+$** in CH_2Cl_2, calculated at the M06-2X/6-31+G** level of theory.

Compound	State	Energy	ΔE
**2aH$^+$(N1)**	S_0 (*EE*)	-1299.867674	
	S_1 (*EE*)	-1299.768235	0.00
	S_1 (*EK*)	-1299.760184	5.05
**2aH$^+$(N3)**	S_0 (*EE*)	-1299.867760	
	S_1 (*EE*)	-1299.768162	0.00
	S_1 (*KE*)	-1299.761222	4.35

Table S9. Wavenumber (ν_i in cm^{-1}), reorganization energy (λ_i in meV), and Huang-Rhys factor (dimensionless) calculated for the protonated compound **2aH⁺** in CH_2Cl_2 solution at the M062X/6-31+G** level of the theory.

**$2aH^+$(N1)-*EE***			**$2aH^+$(N3)-*EE***		
ν_i	λ_i	HR	ν_i	λ_i	HR
18	2	0.90	17	2	0.95
33	2	0.49	26	1	0.31
36	6	1.34	28	1	0.29
40	1	0.20	31	5	1.30
87	1	0.09	35	1	0.23
135	3	0.18	119	2	0.14
163	1	0.05	127	1	0.06
256	7	0.22	159	1	0.05
270	1	0.03	226	1	0.04
485	1	0.02	228	1	0.04
507	1	0.02	248	1	0.03
547	3	0.04	370	2	0.04
556	1	0.01	490	2	0.03
590	1	0.01	503	1	0.02
654	1	0.01	546	2	0.03
879	1	0.01	554	1	0.01
891	3	0.03	562	1	0.01
1032	9	0.07	587	1	0.01
1175	1	0.01	656	2	0.02
1182	1	0.01	690	2	0.02
1193	17	0.11	742	1	0.01
1198	1	0.01	876	1	0.01
1204	2	0.01	888	2	0.02
1211	1	0.01	890	1	0.01
1259	3	0.02	974	1	0.01
1314	3	0.02	976	1	0.01
1326	1	0.01	1031	8	0.06
1331	1	0.01	1128	1	0.01
1359	8	0.05	1194	19	0.13
1362	5	0.03	1195	1	0.01
1366	4	0.02	1254	2	0.01
1376	1	0.01	1292	1	0.01
1402	1	0.01	1308	3	0.02
1411	2	0.01	1325	4	0.02
1443	7	0.04	1326	2	0.01
1456	1	0.01	1346	2	0.01
1477	10	0.05	1352	7	0.04
1493	1	0.01	1366	3	0.02
1563	5	0.03	1371	5	0.03
1571	1	0.01	1440	2	0.01
1631	17	0.08	1466	1	0.01
1636	8	0.04	1476	7	0.04
1653	13	0.06	1492	1	0.01
1662	2	0.01	1526	4	0.02
1686	17	0.08	1548	3	0.02
1692	1	0.00	1559	7	0.04
1700	1	0.00	1605	28	0.14
1713	10	0.05	1639	1	0.00
3448	1	0.00	1652	4	0.02
3509	1	0.00	1662	7	0.03
			1687	8	0.04
			1693	1	0.00
			1713	8	0.04

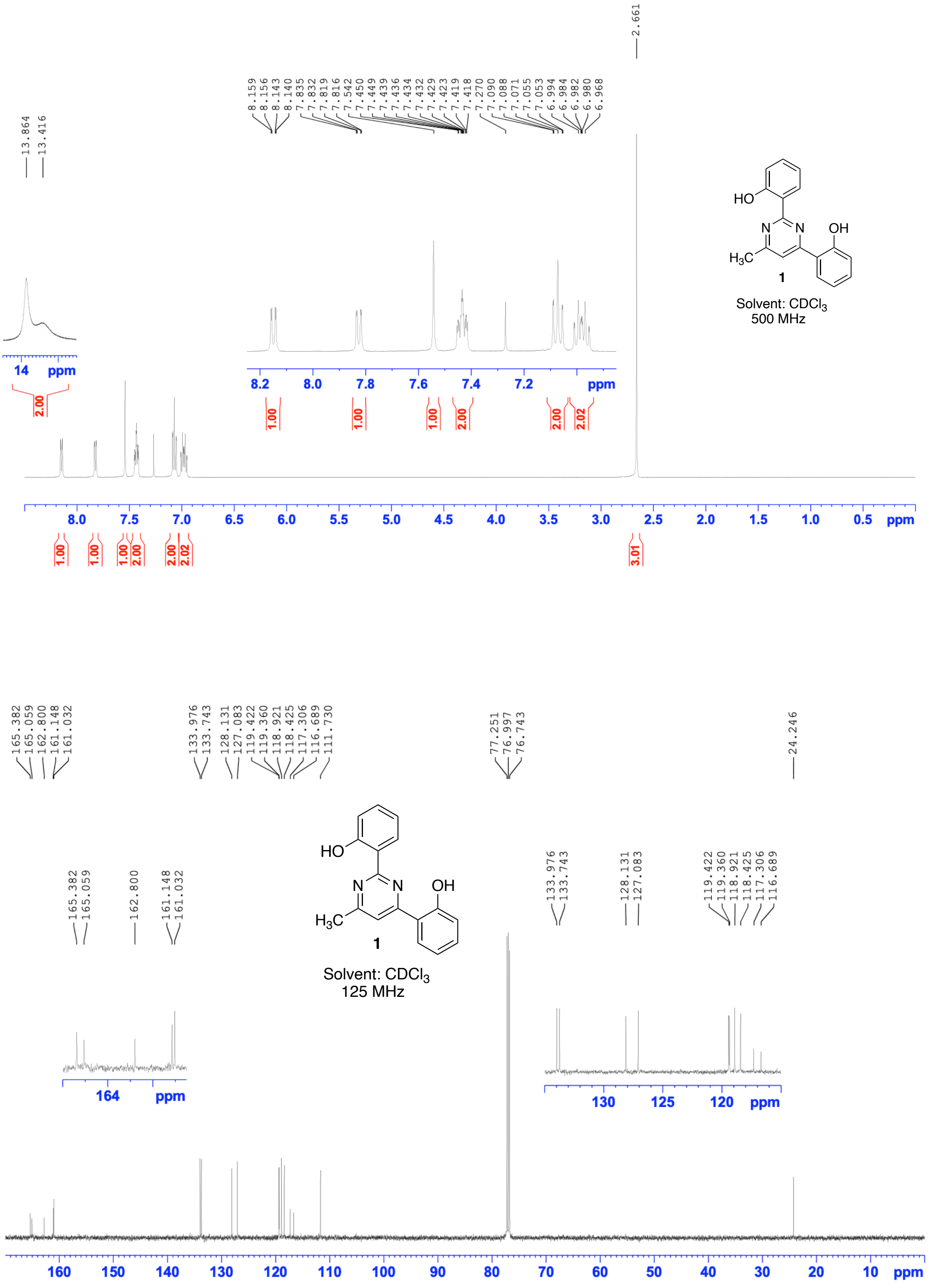

Figure S1. ^{1}H and ^{13}C NMR spectra of **1**.

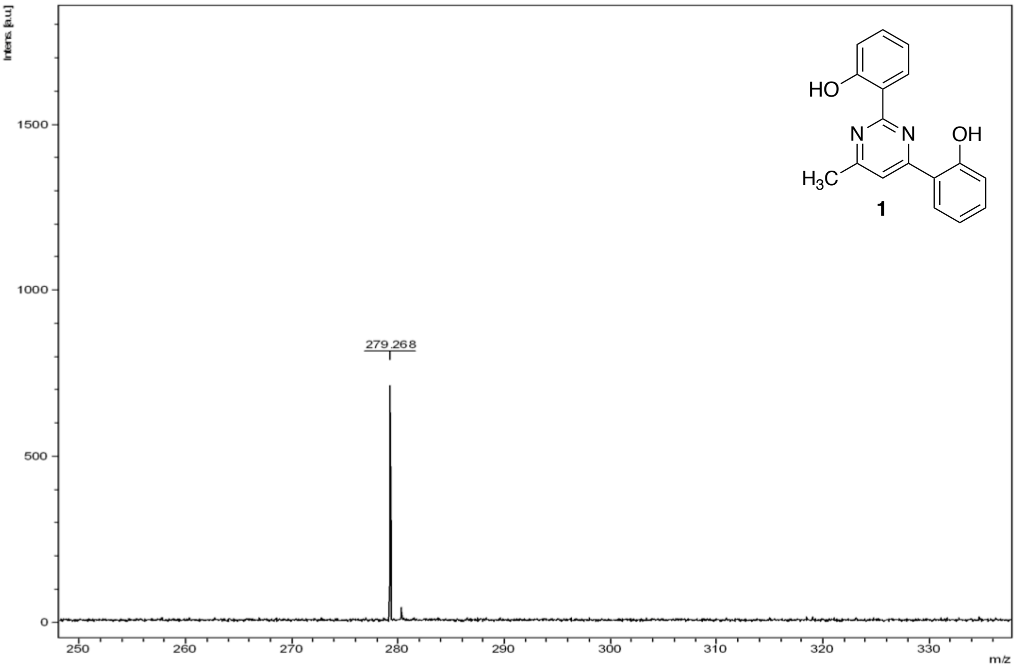

m/z	Intens.	Res.
279.268	780.24	4486

Figure S2. MALDI-TOF MS (dithranol) spectrum of **1**.

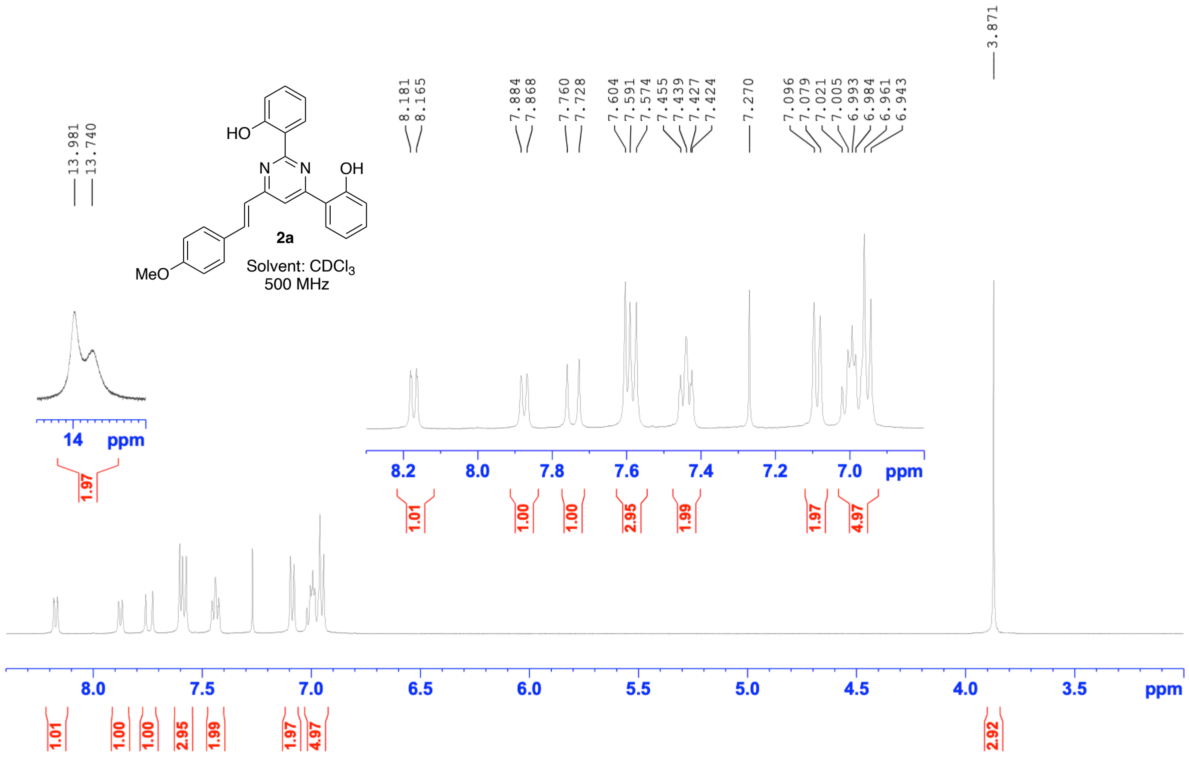

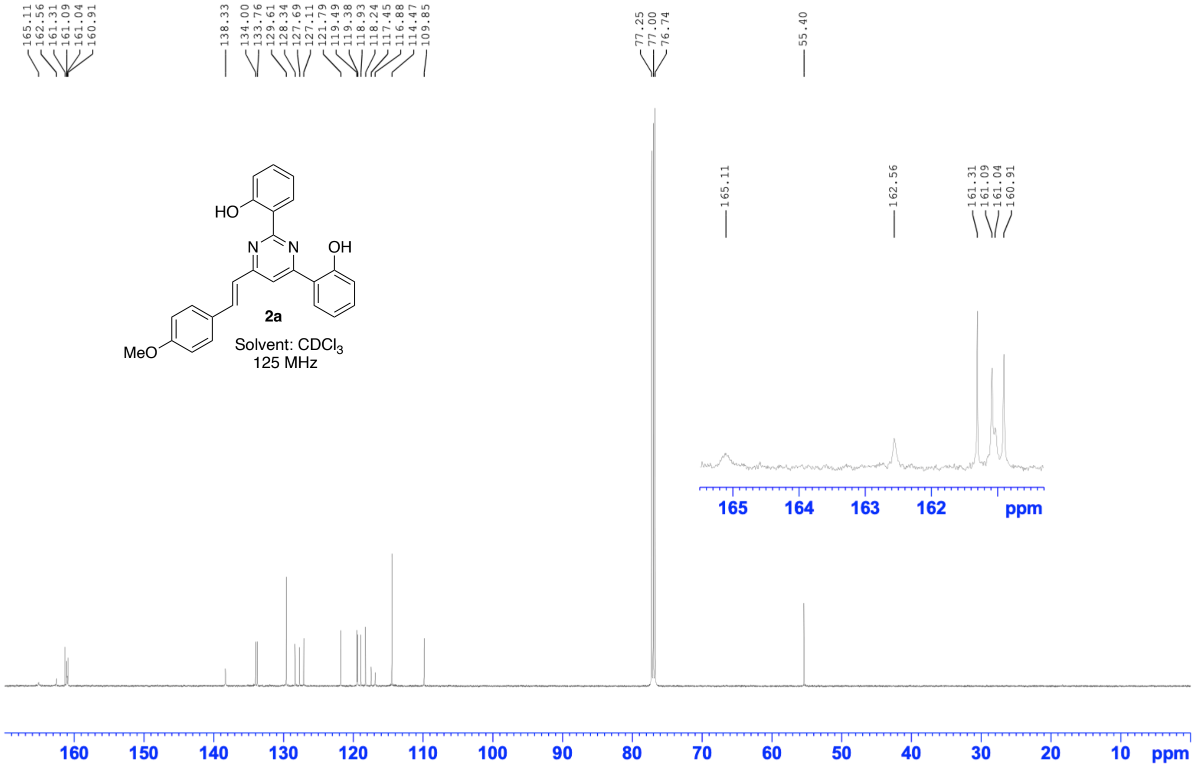

Figure S3. ^{1}H and ^{13}C NMR spectra of **2a**.

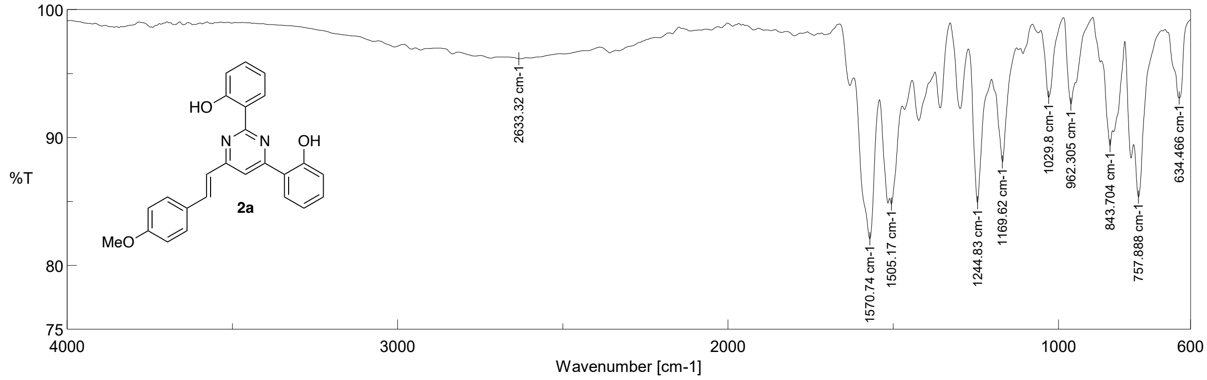

Figure S4. IR spectrum of **2a**.

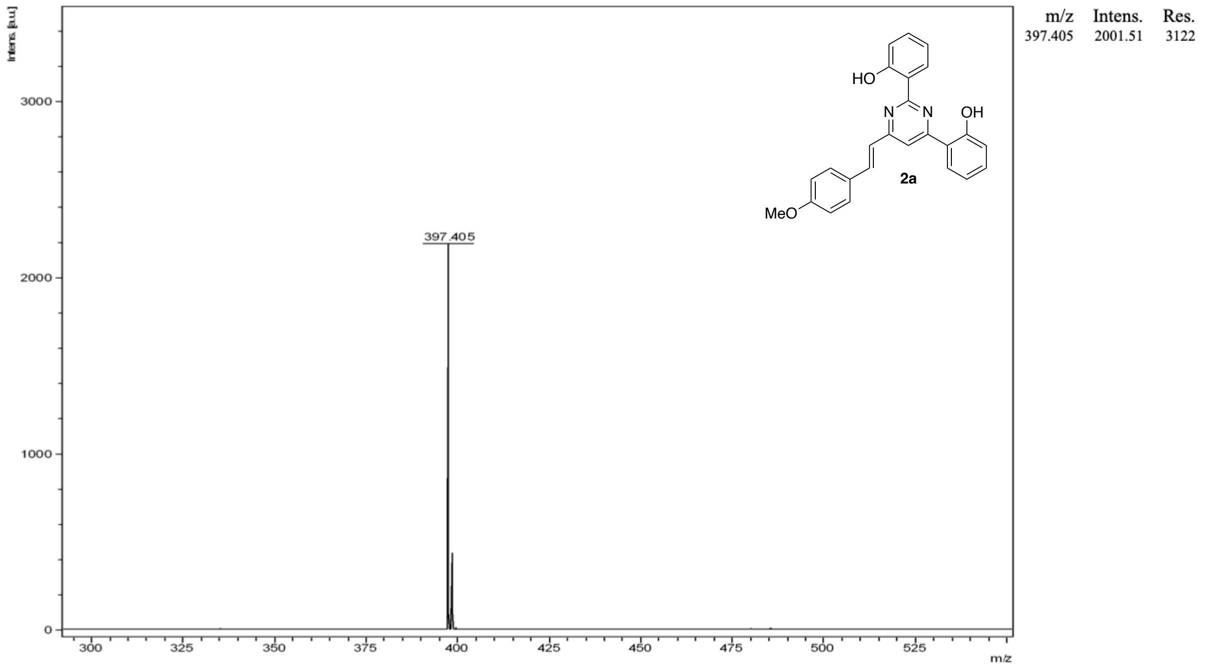

Figure S5. MALDI-TOF MS (dithranol) spectrum of **2a**.

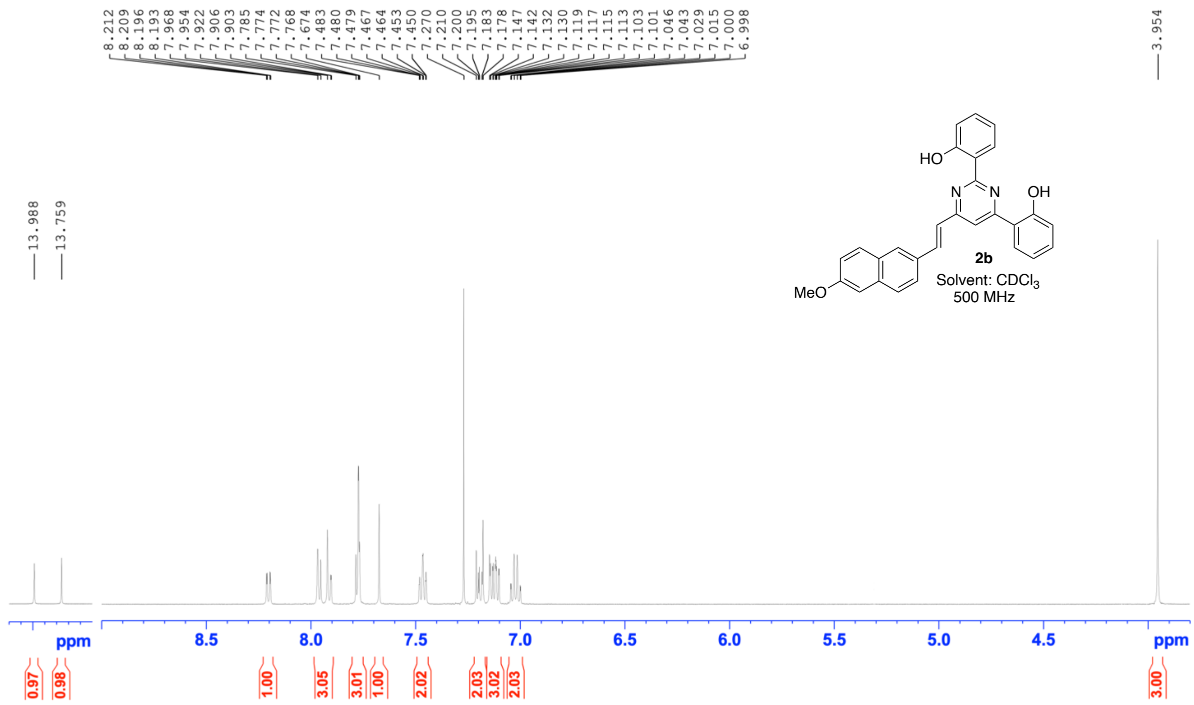

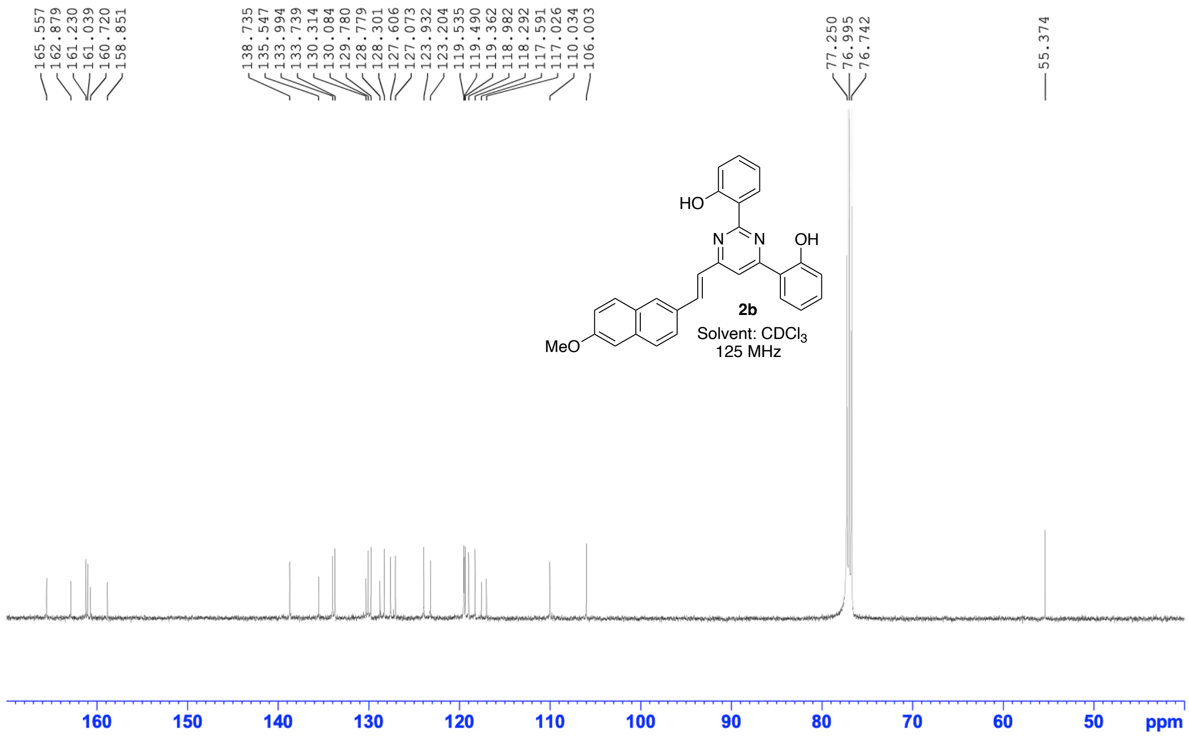

Figure S6. ^{1}H and ^{13}C NMR spectra of **2b**.

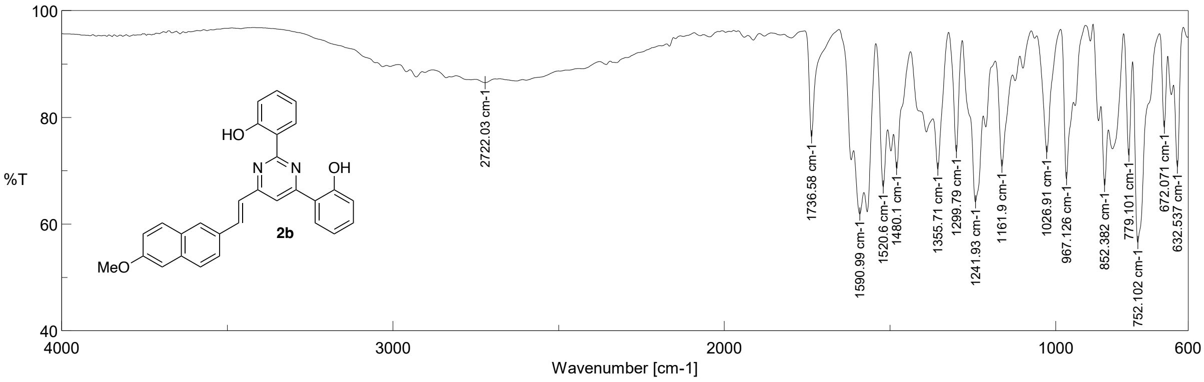

Figure S7. IR spectrum of **2b**.

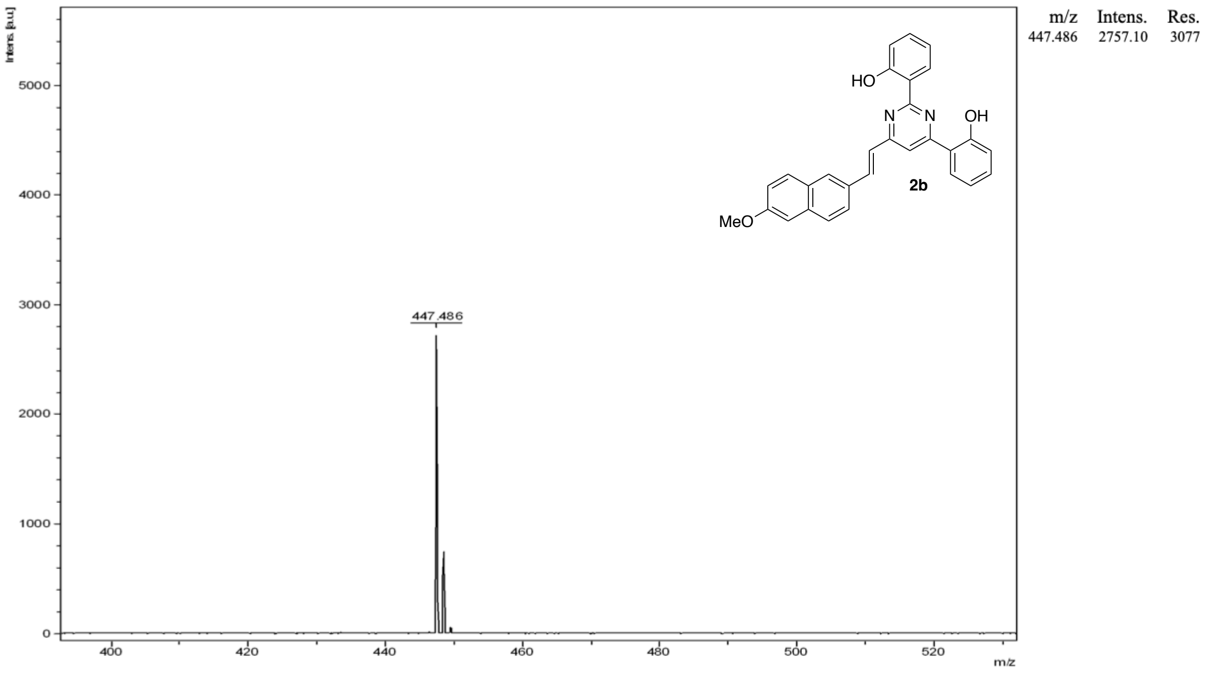

Figure S8. MALDI-TOF MS (dithranol) spectrum of **2b**.

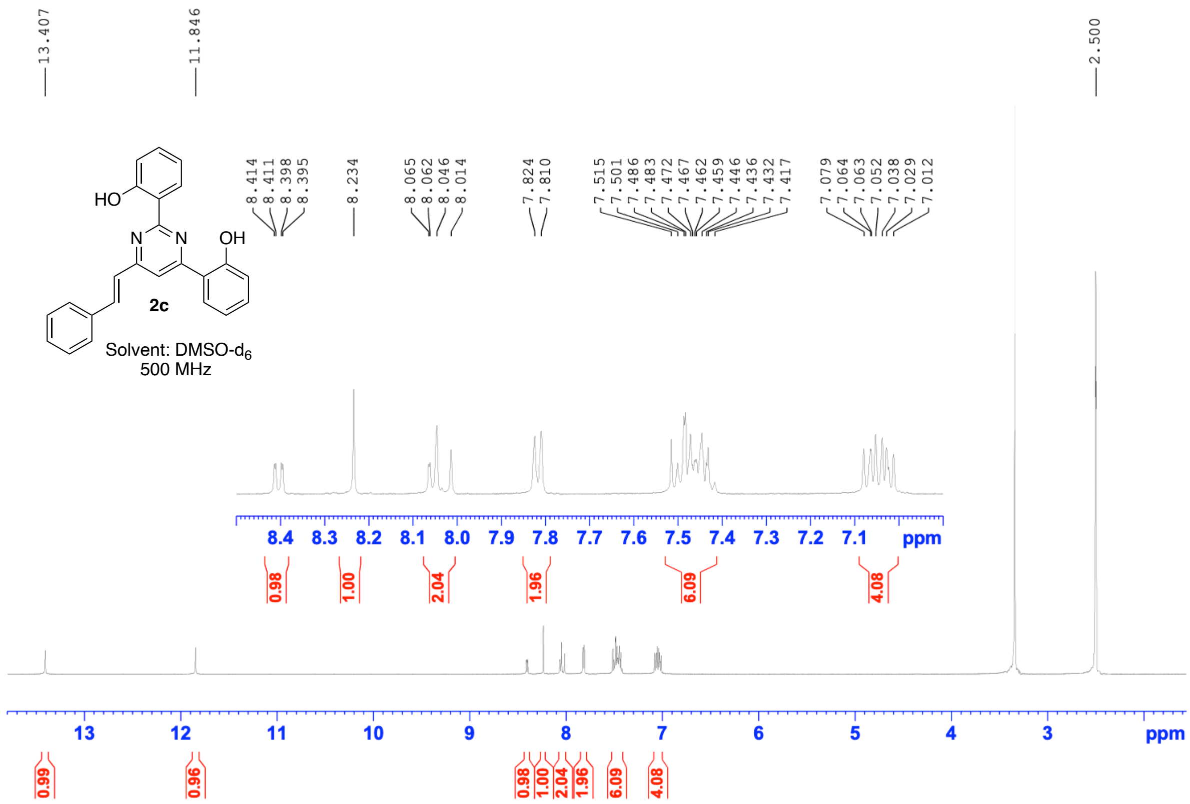

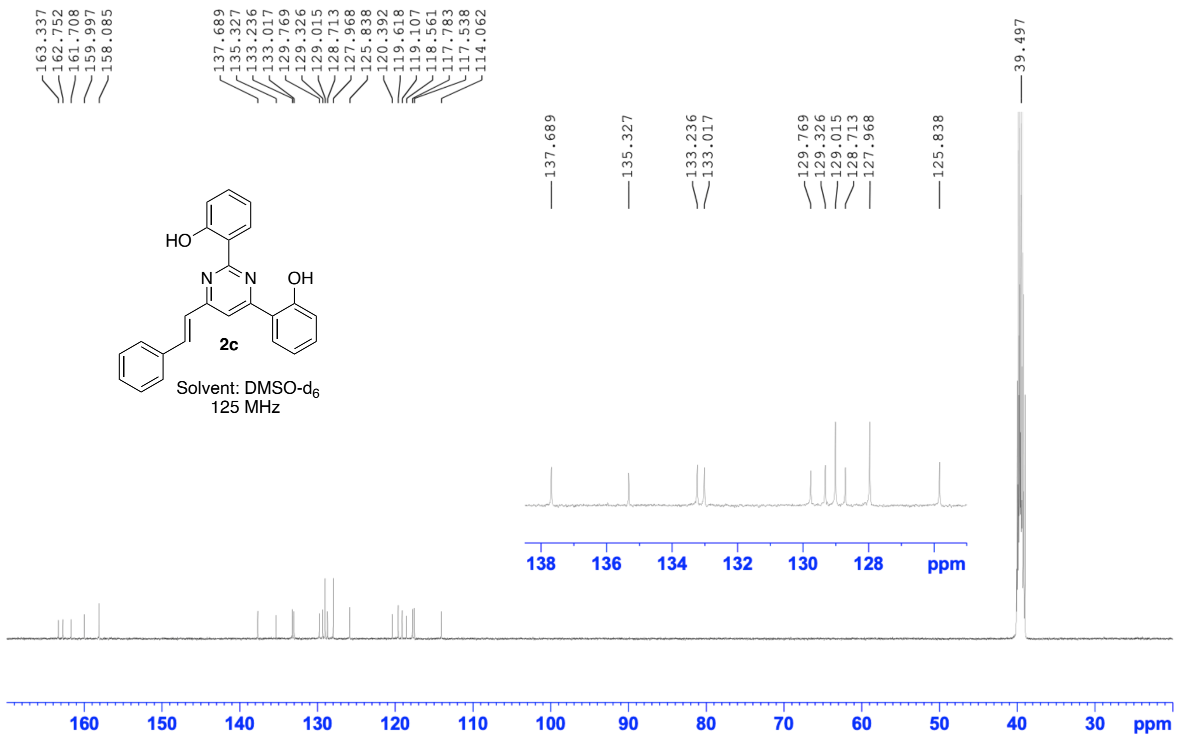

Figure S9. ^{1}H and ^{13}C NMR spectra of **2c**.

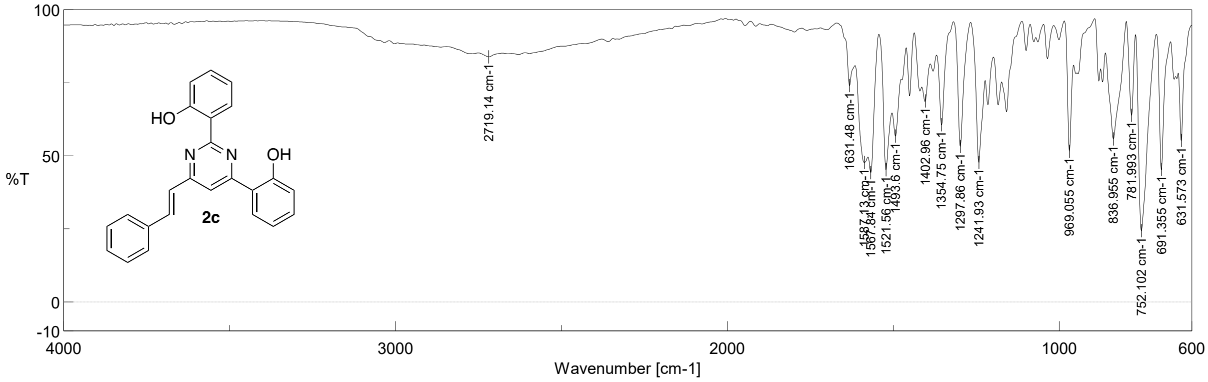

Figure S10. IR spectrum of **2c**.

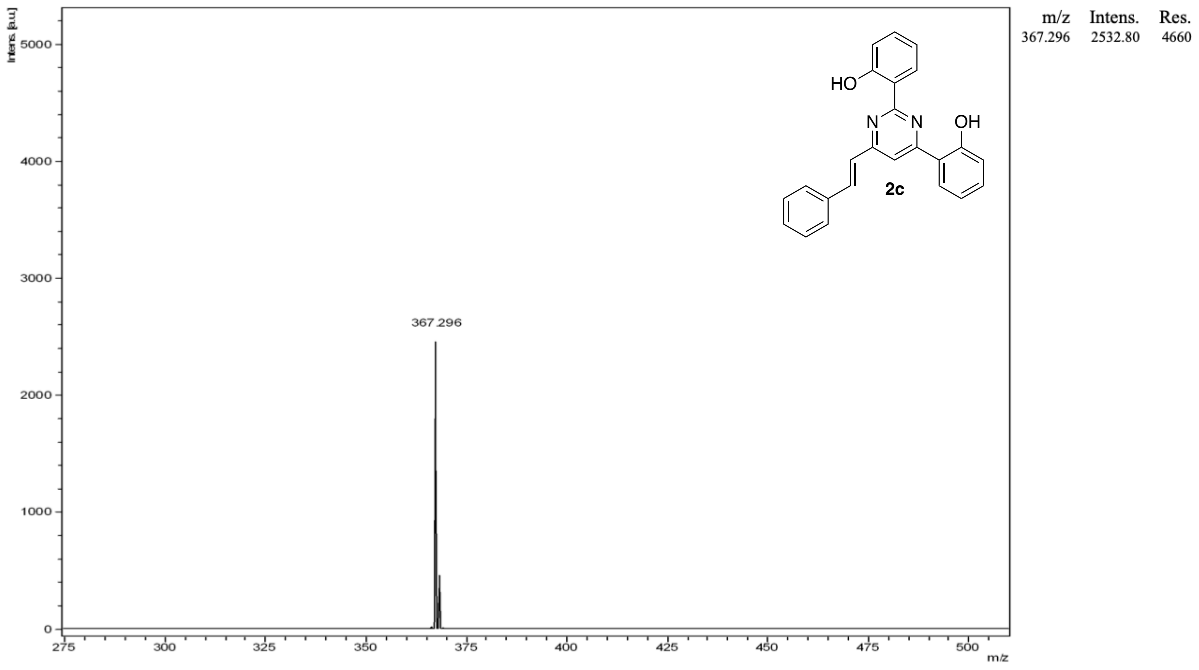

Figure S11. MALDI-TOF MS (dithranol) spectrum of **2c**.

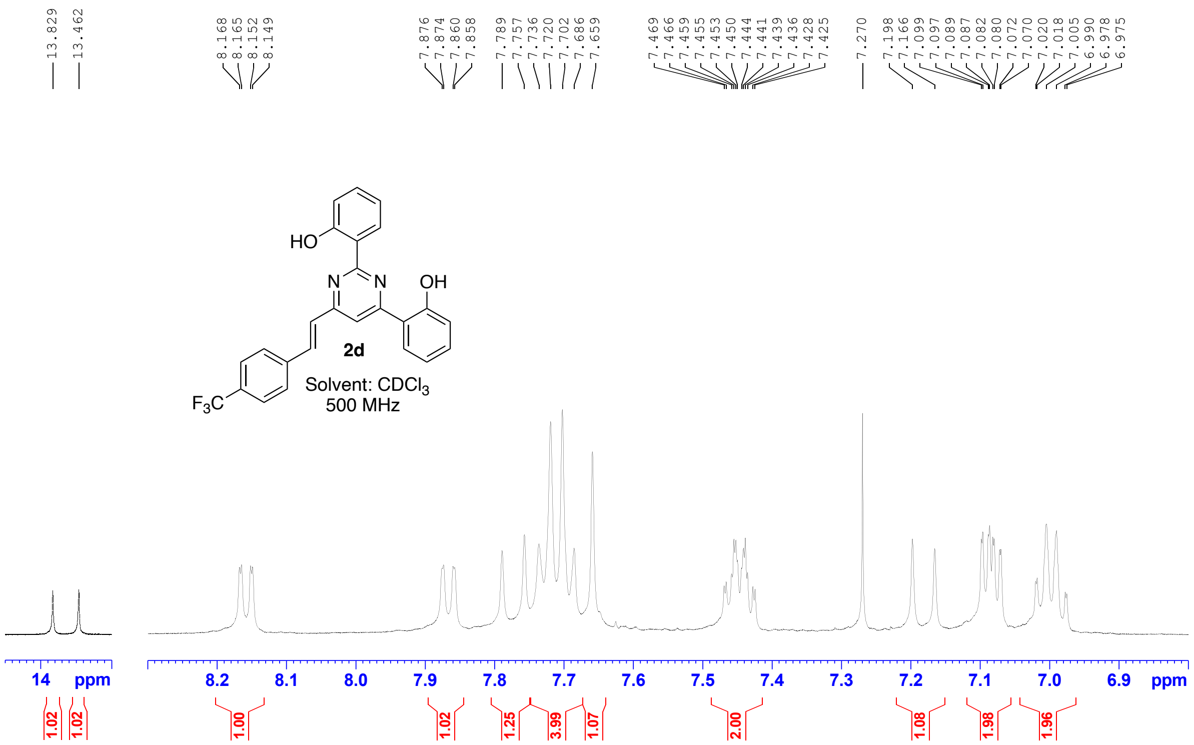

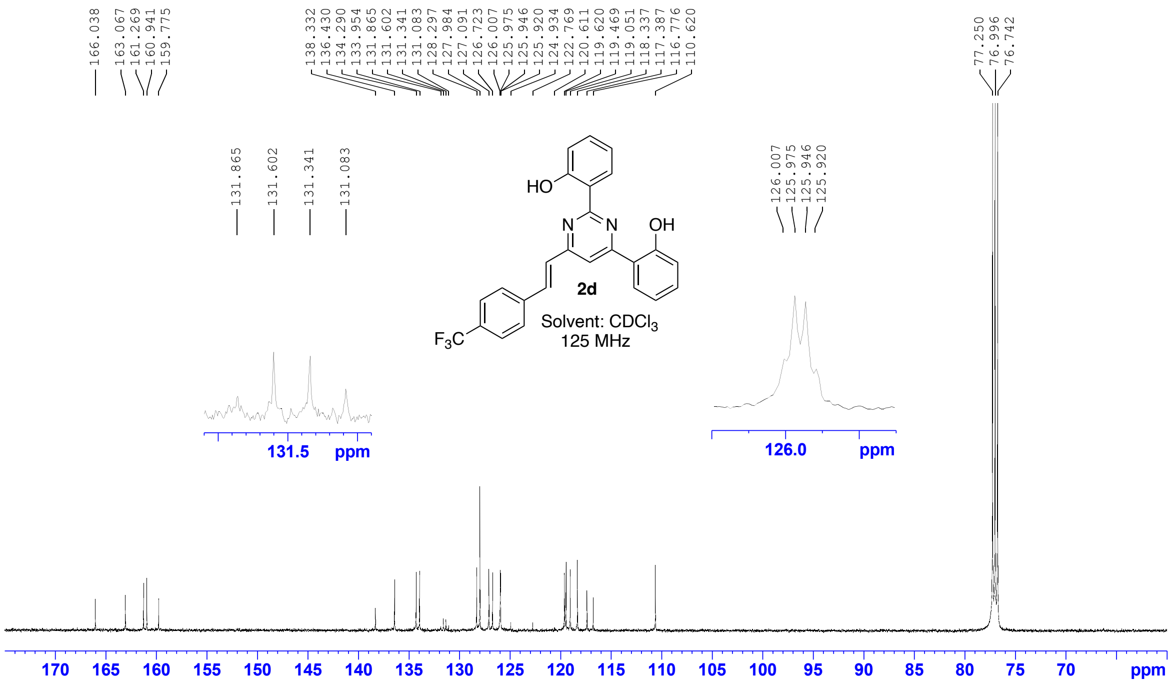

Figure S12. ^{1}H and ^{13}C NMR spectra of **2d**.

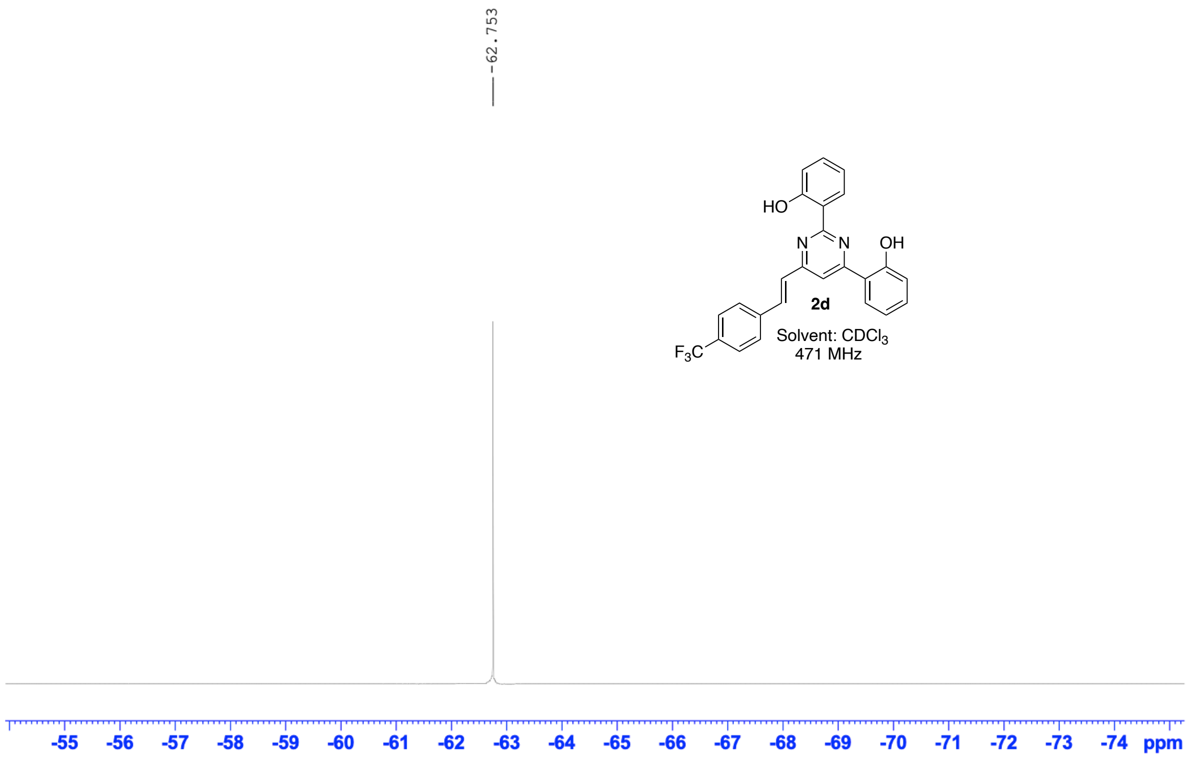

Figure S13. ^{19}F NMR spectra of **2d**.

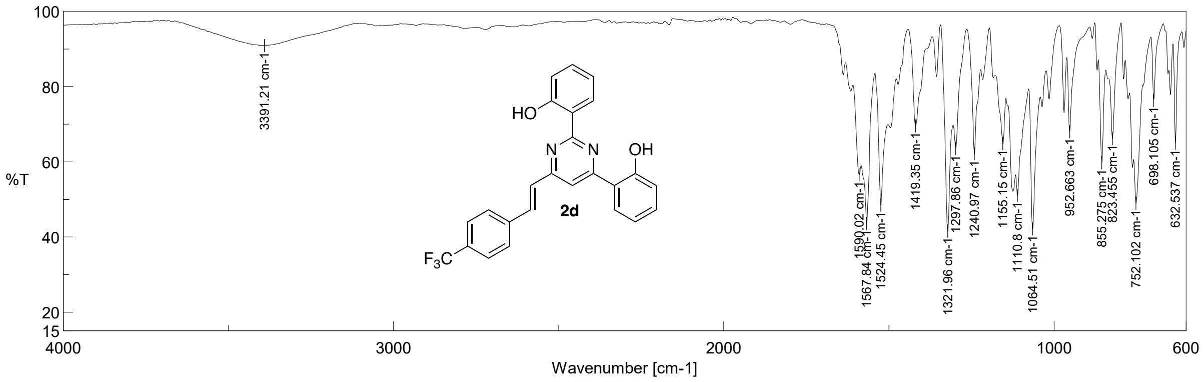

Figure S14. IR spectrum of **2d**.

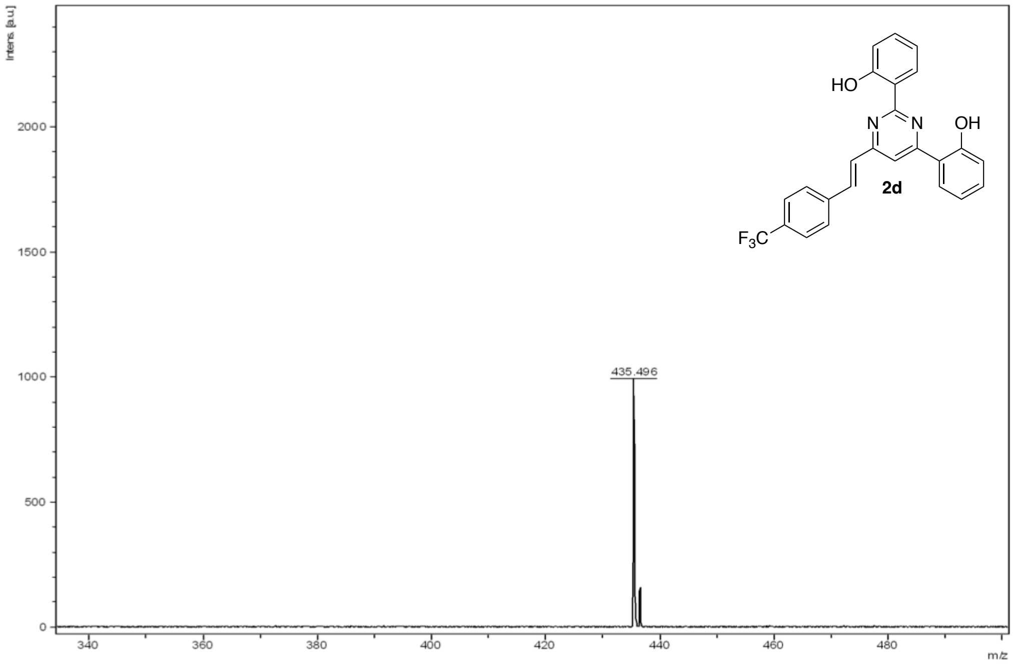

m/z	Intens.	Res.
435.496	859.30	2107
227.365	2156.40	1087

Figure S15. MALDI-TOF MS (dithranol) spectrum of **2d**.

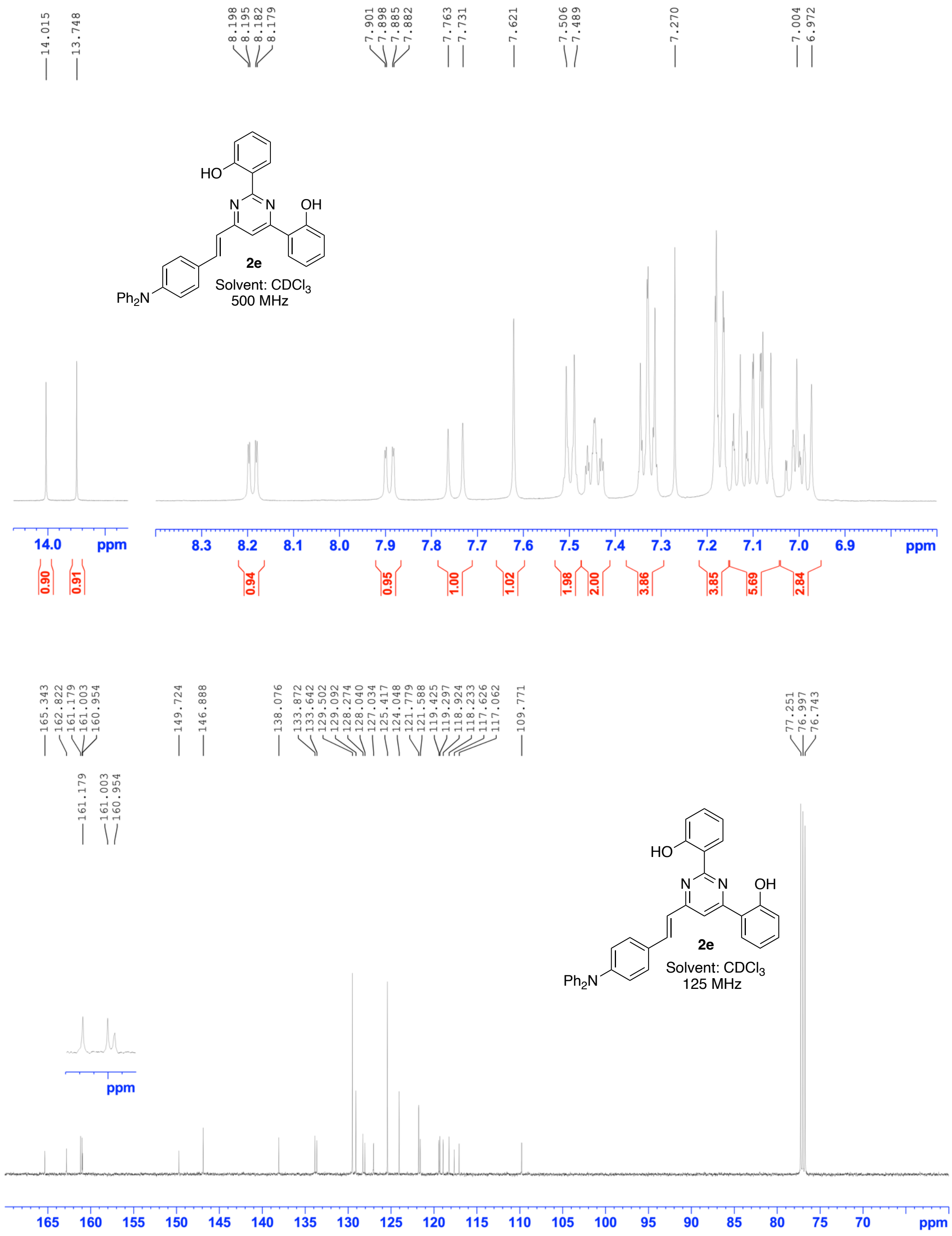

Figure S16. ^1H and ^{13}C NMR spectra of **2e**.

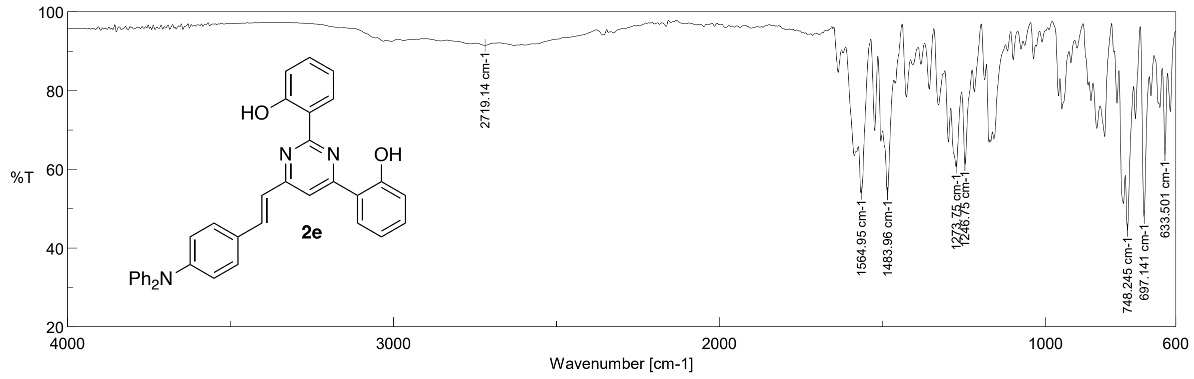

Figure S17. IR spectrum of **2e**.

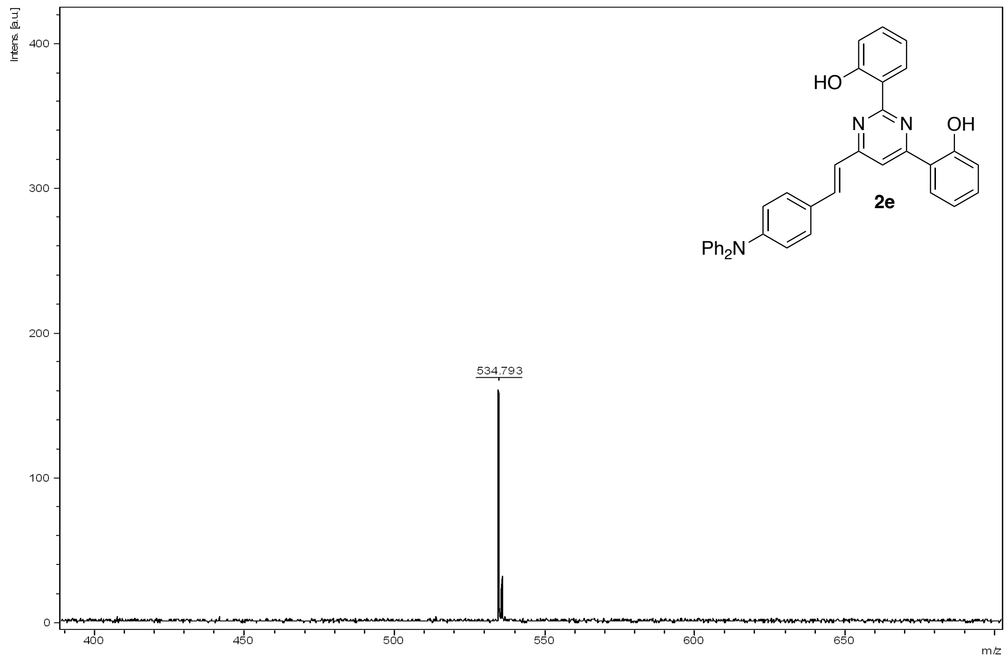

m/z	Intens.	Res.
534.793	164.93	3626

Figure S18. MALDI-TOF MS (dithranol) spectrum of **2e**.

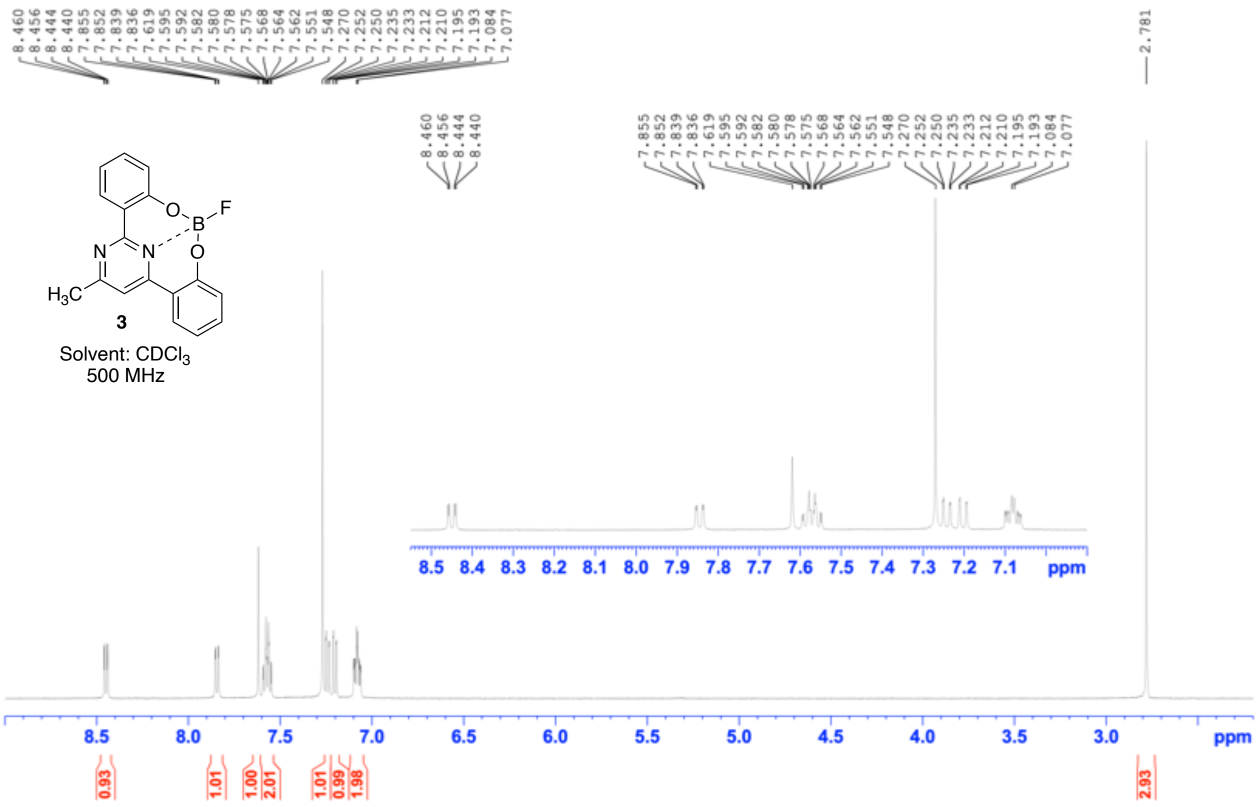

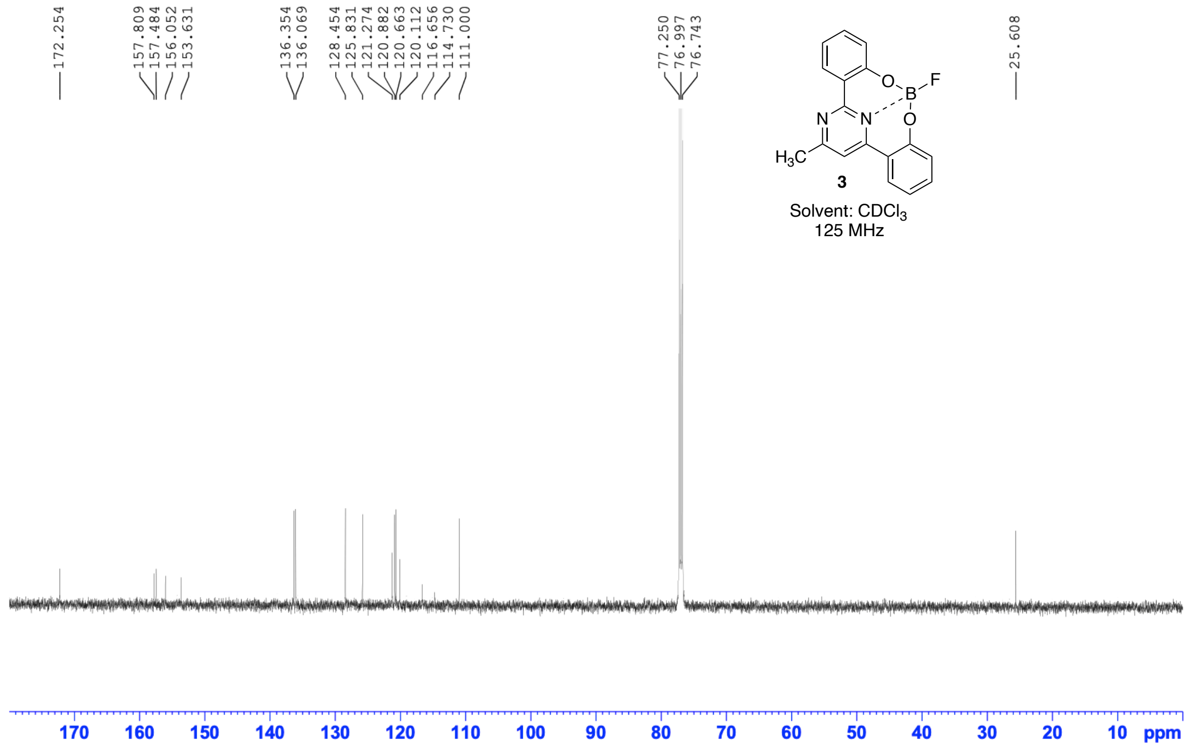

Figure S19. ^{1}H and ^{13}C NMR spectra of **3**.

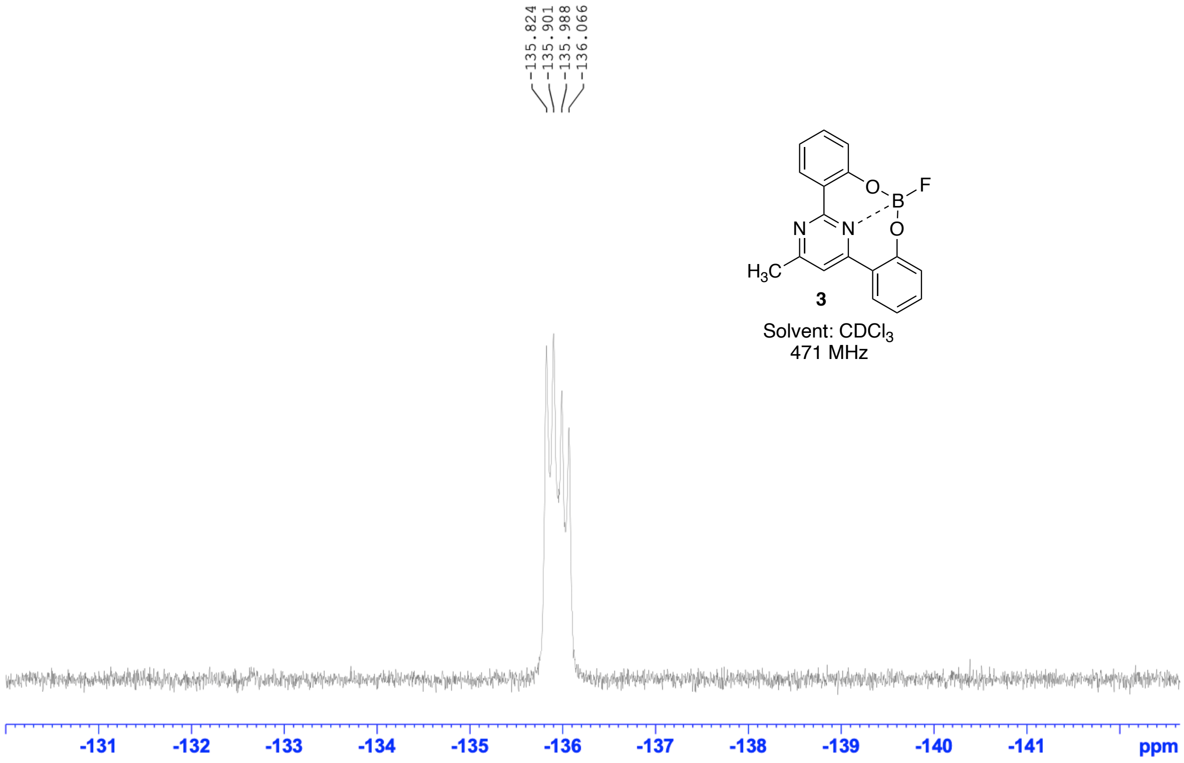

Figure S20. ^{19}F NMR spectra of **3**.

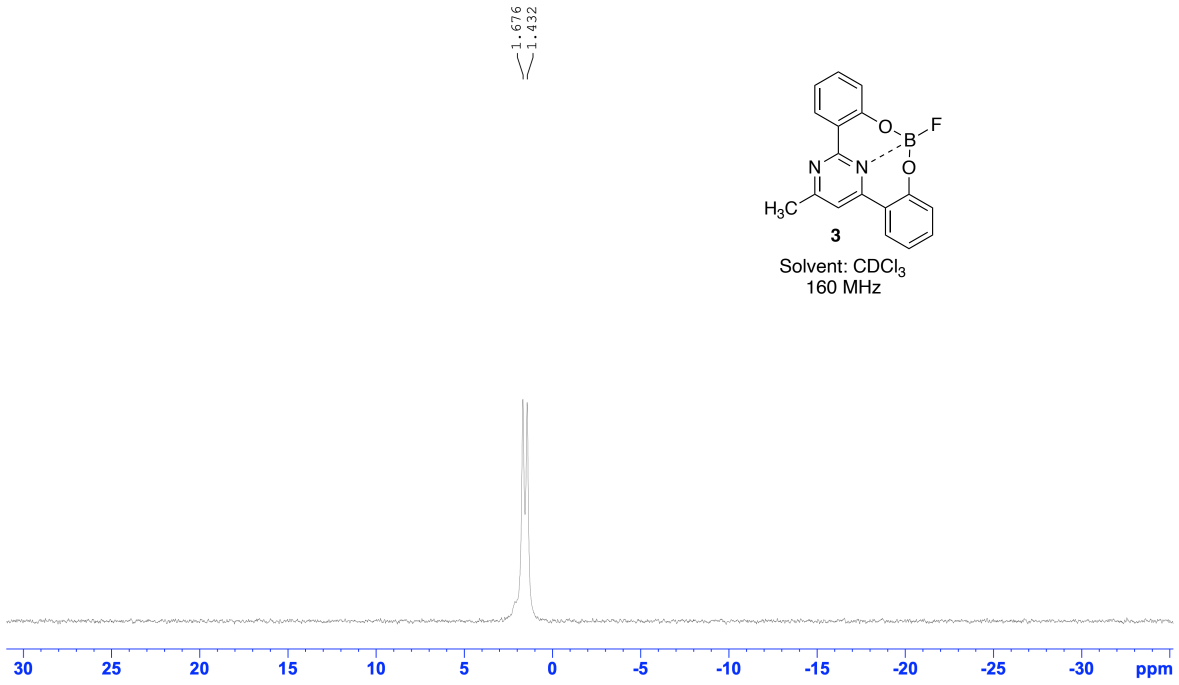

Figure S21. ^{11}B NMR spectra of **3**.

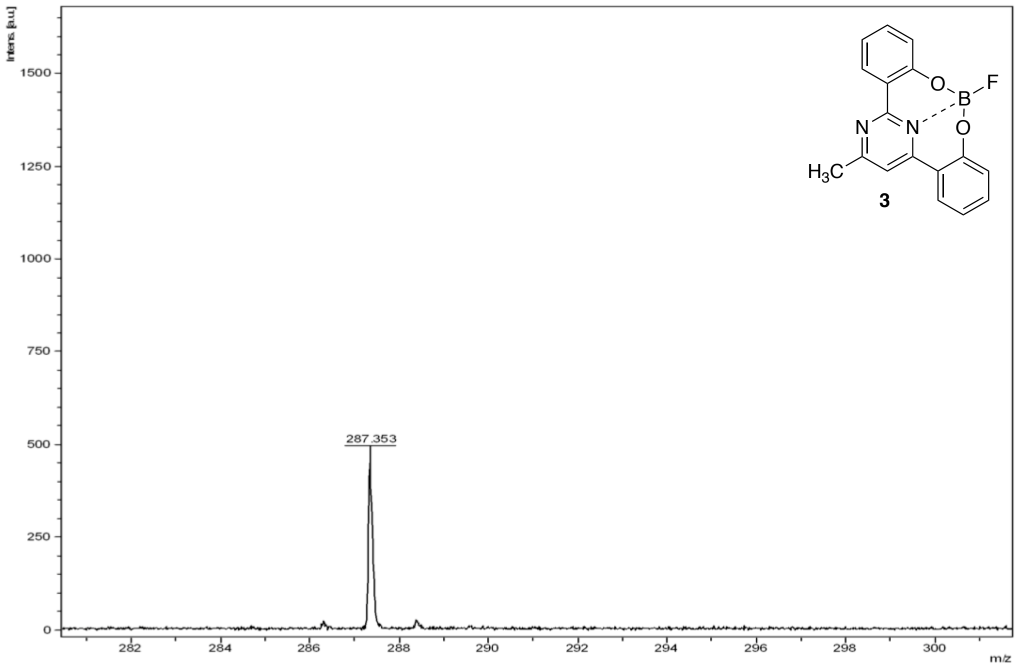

m/z	Intens.	Res.
287.353	407.46	2644

Figure S22. MALDI-TOF MS (dithranol) spectrum of **3**.

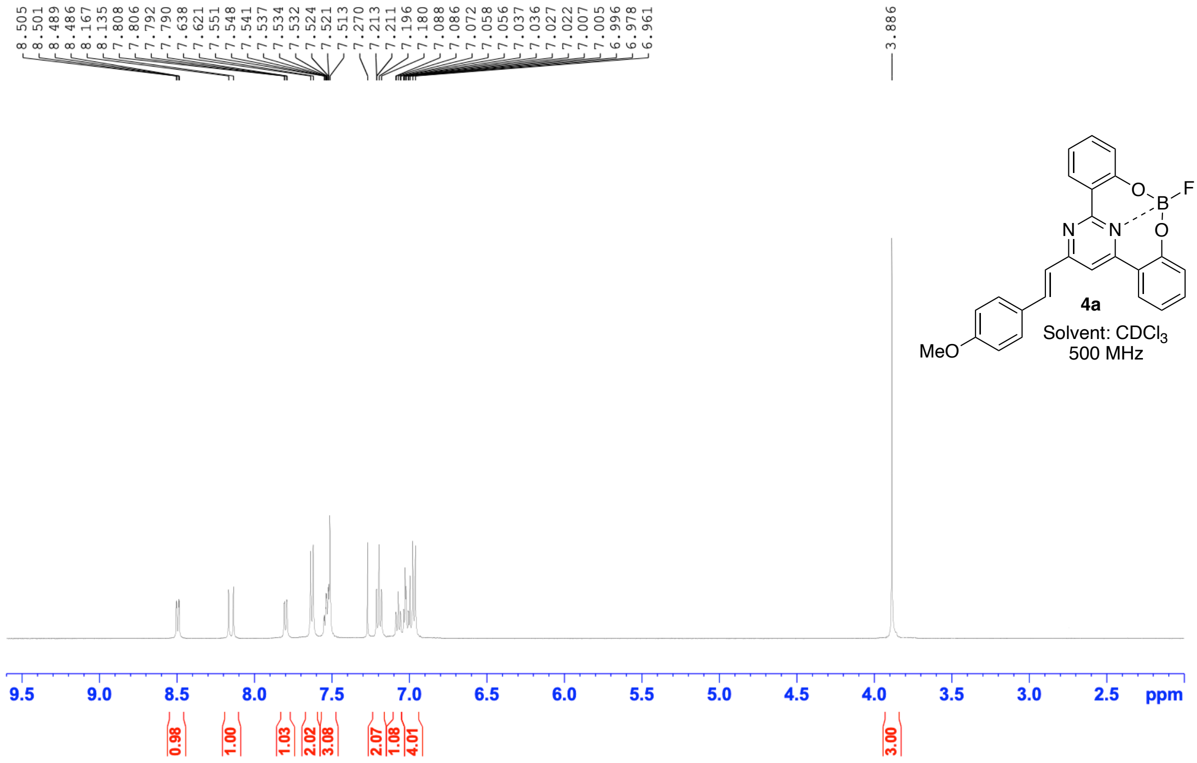

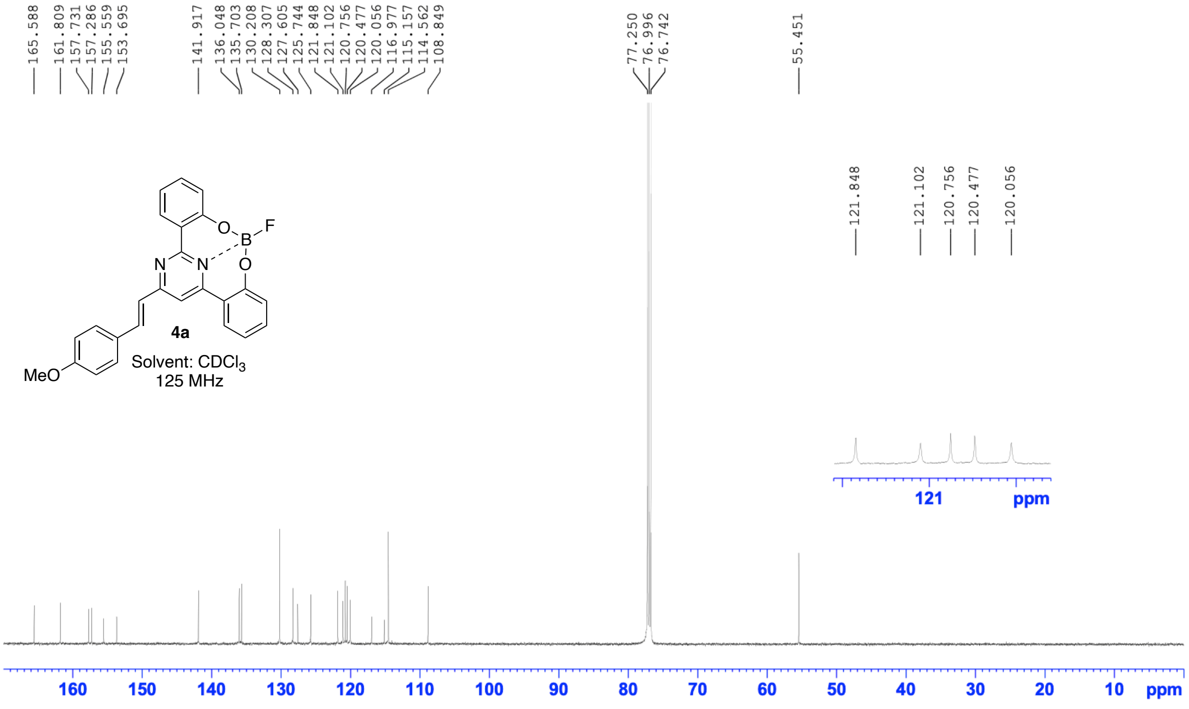

Figure S23. ^{1}H and ^{13}C NMR spectra of **4a**.

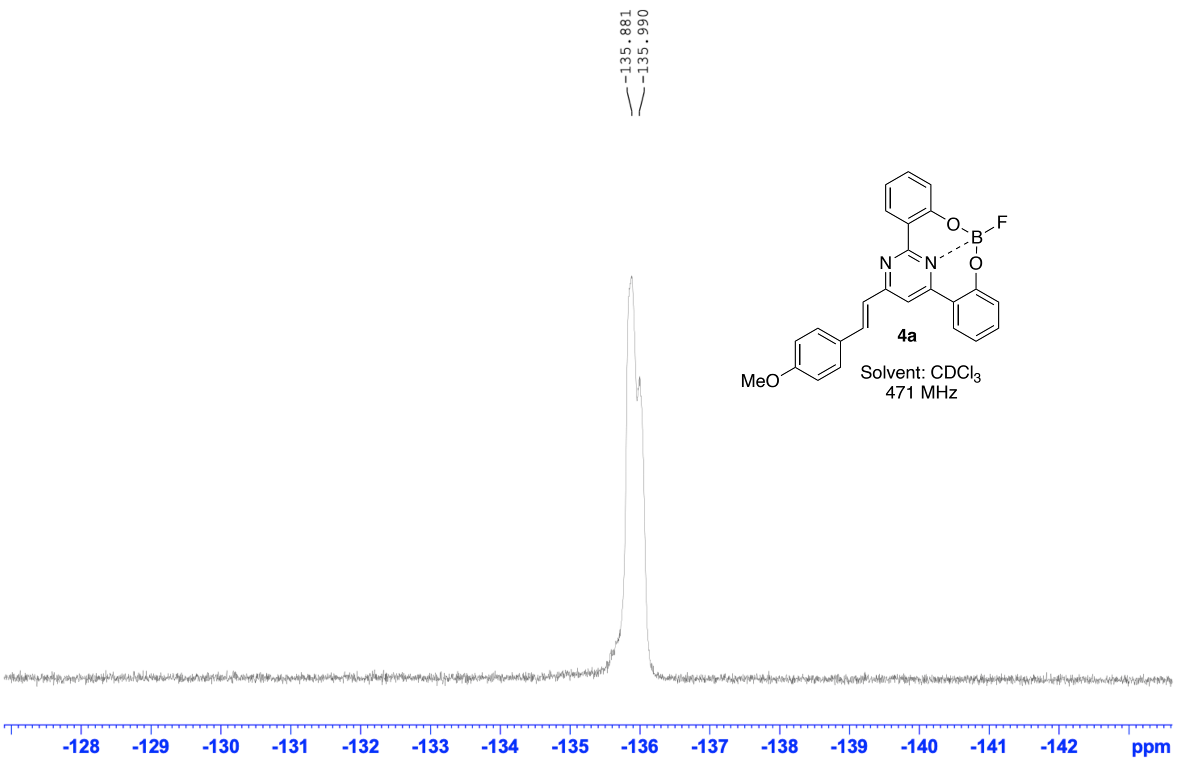

Figure S24. ^{19}F NMR spectra of **4a**.

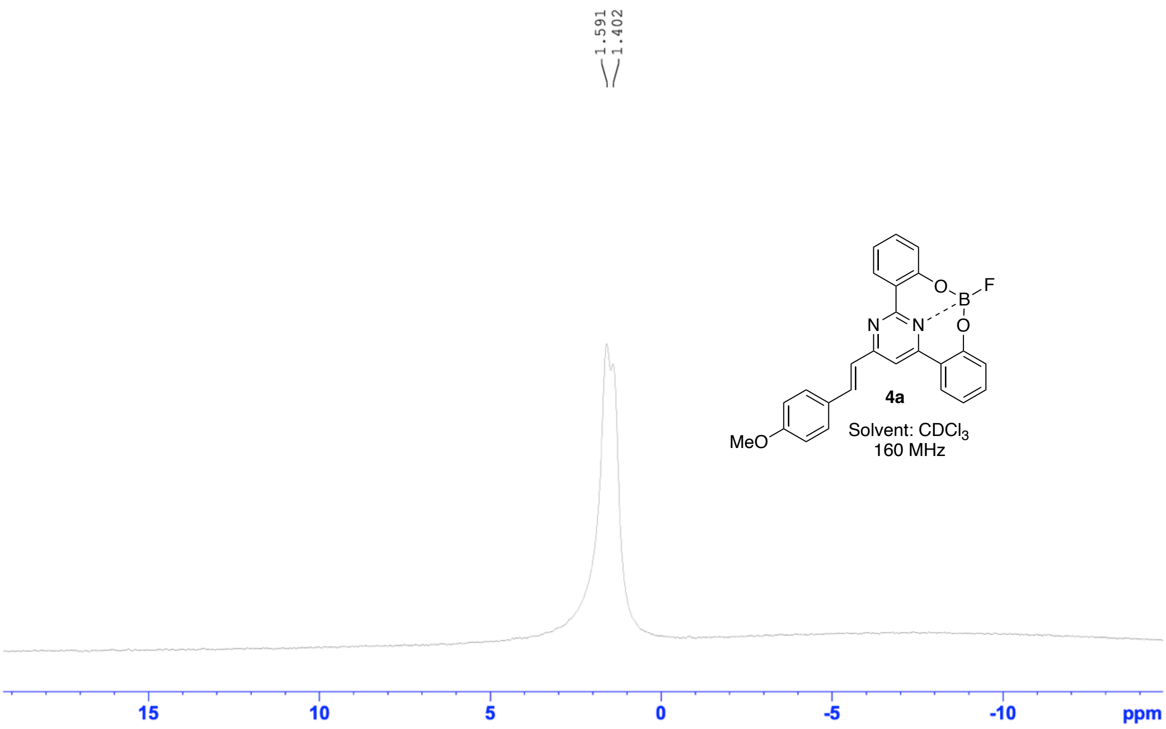

Figure S25. ^{11}B NMR spectra of **4a**.

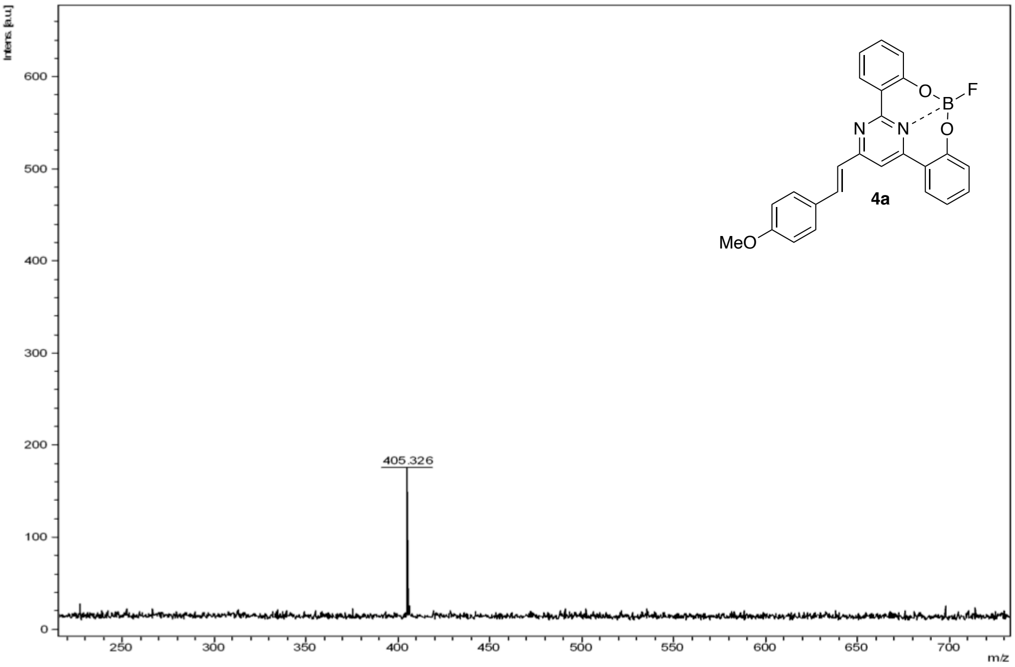

m/z	Intens.	Res.
405.326	137.51	4142

Figure S26. MALDI-TOF MS (dithranol) spectrum of **4a**.

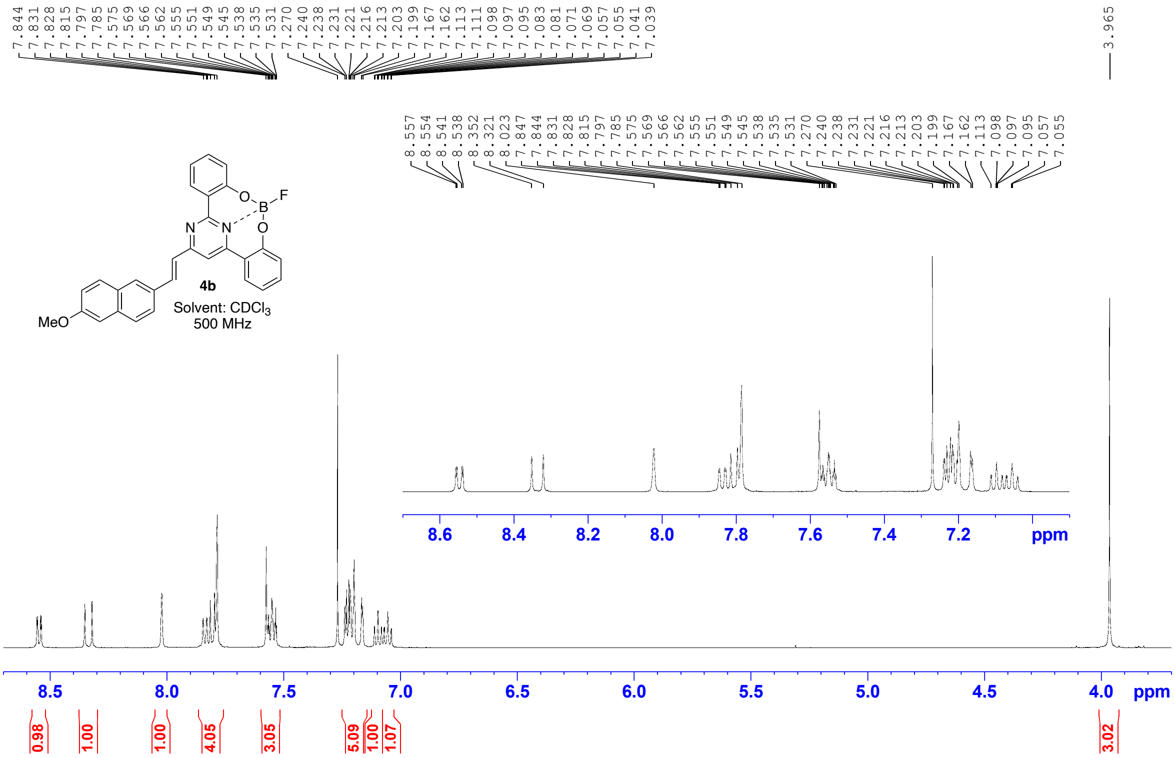

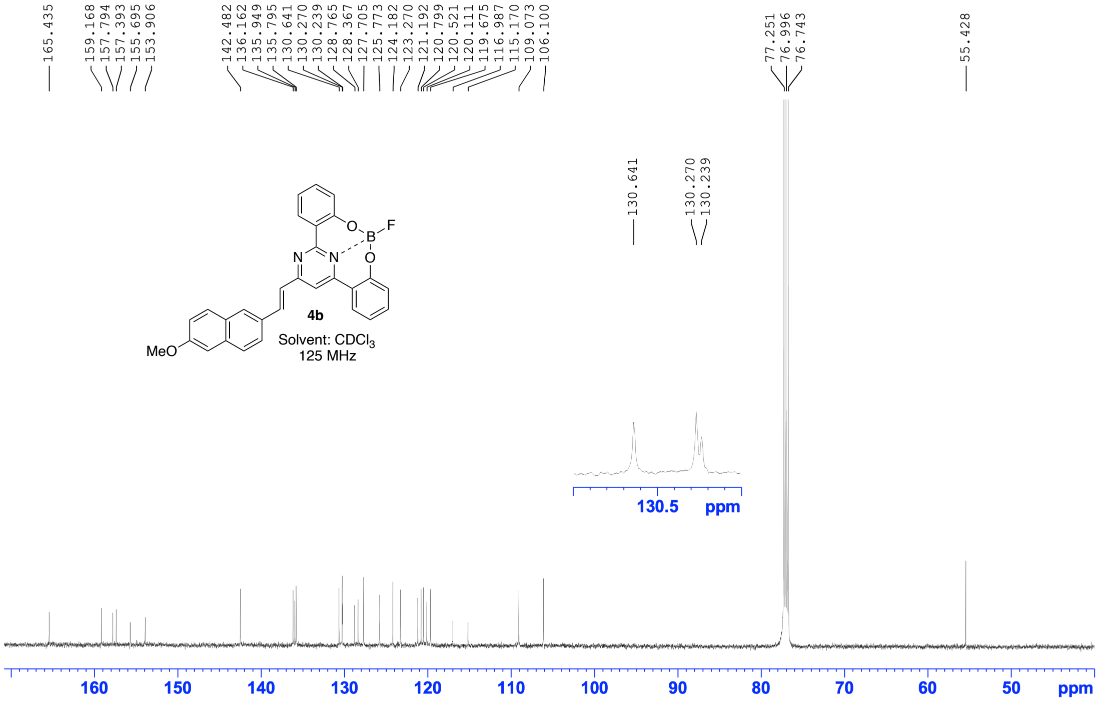

Figure S27. ^{1}H and ^{13}C NMR spectra of **4b**.

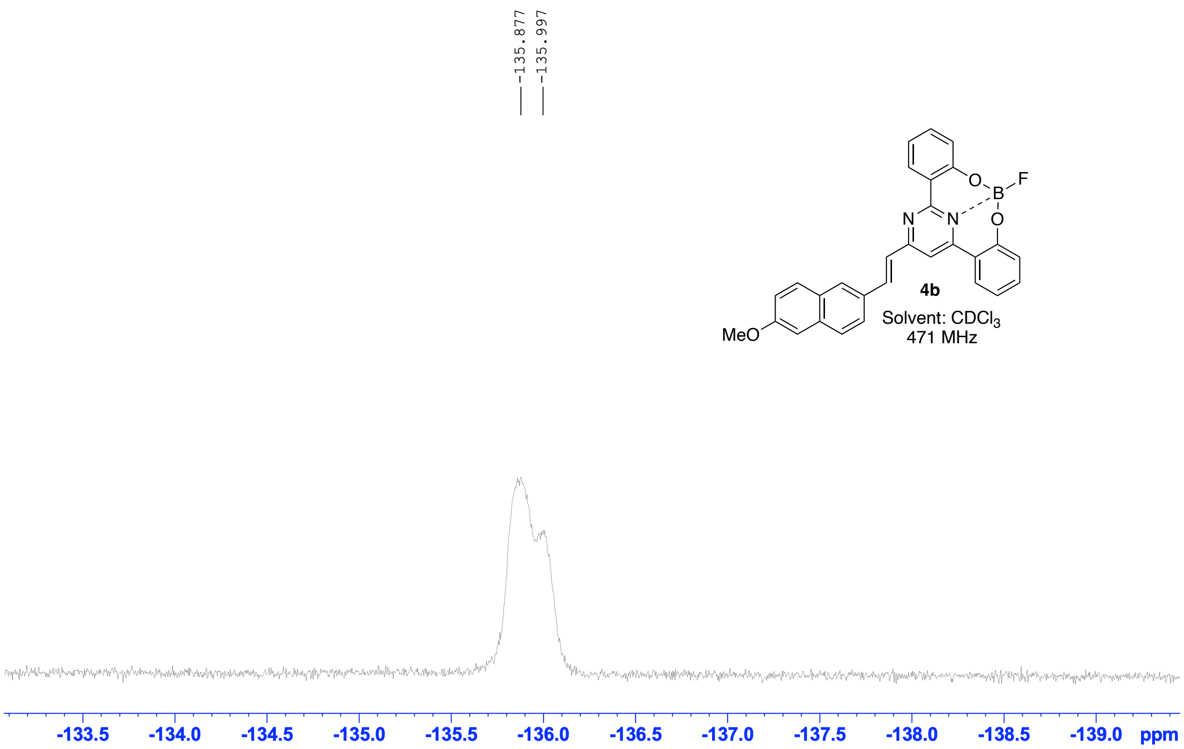

Figure S28. ^{19}F NMR spectra of **4b**.

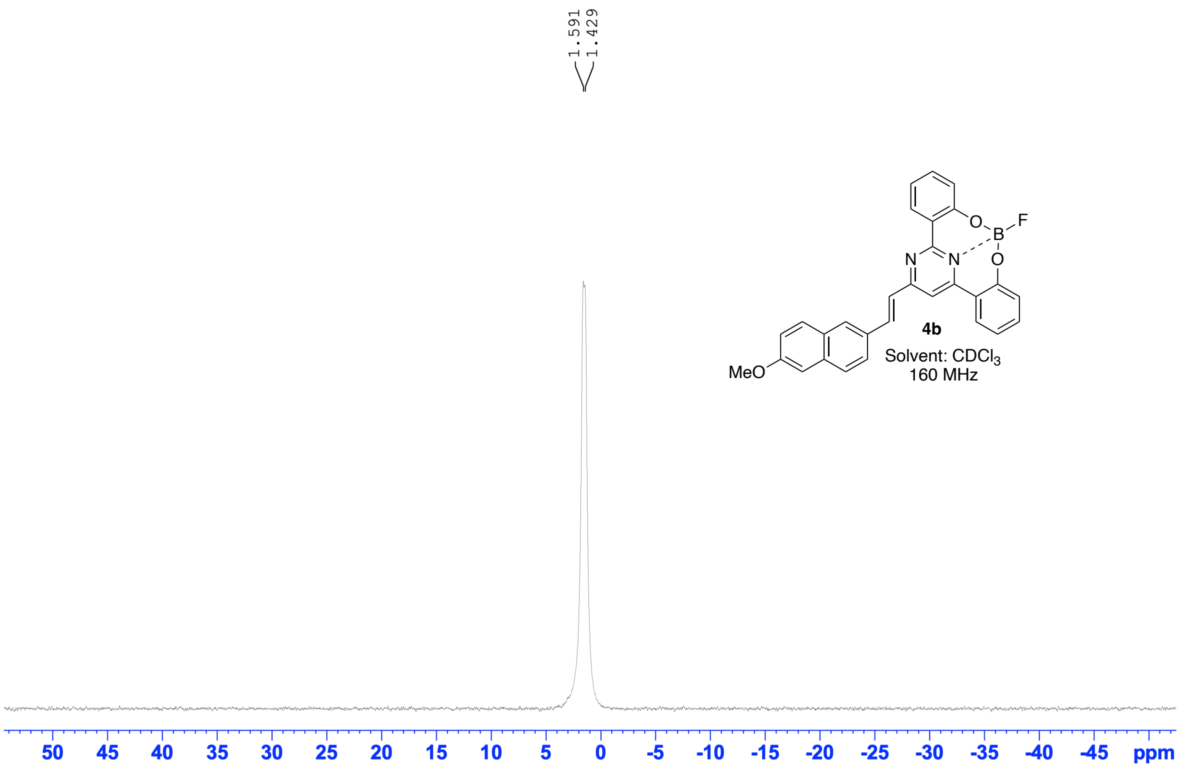

Figure S29. ^{11}B NMR spectra of **4b**.

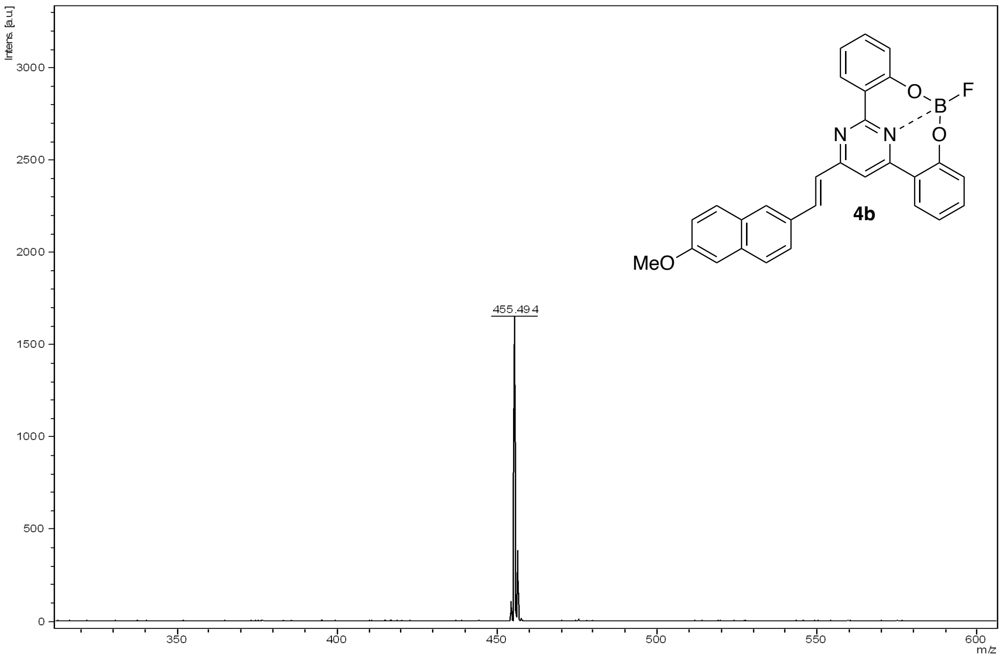

m/z	Intens.	Res.
455.494	1466.37	1561

Figure S30. MALDI-TOF MS (dithranol) spectrum of **4b**.

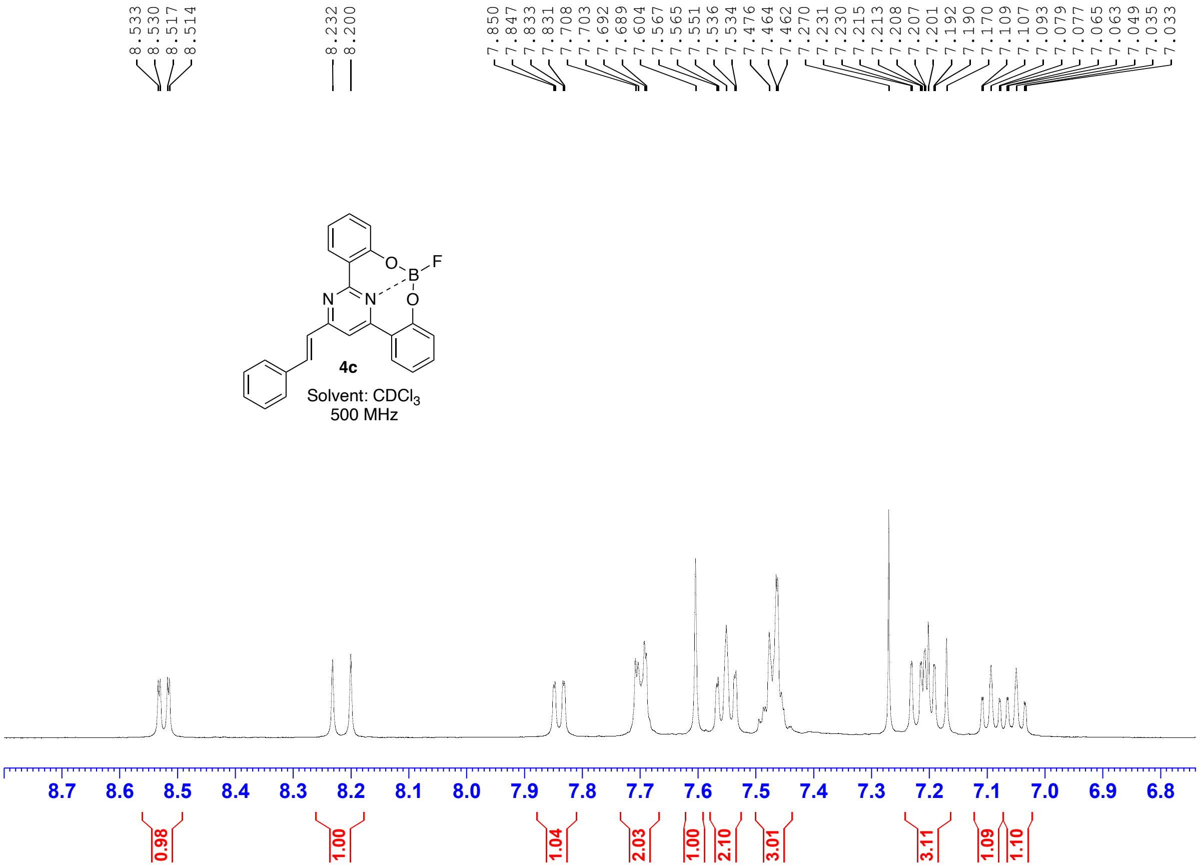

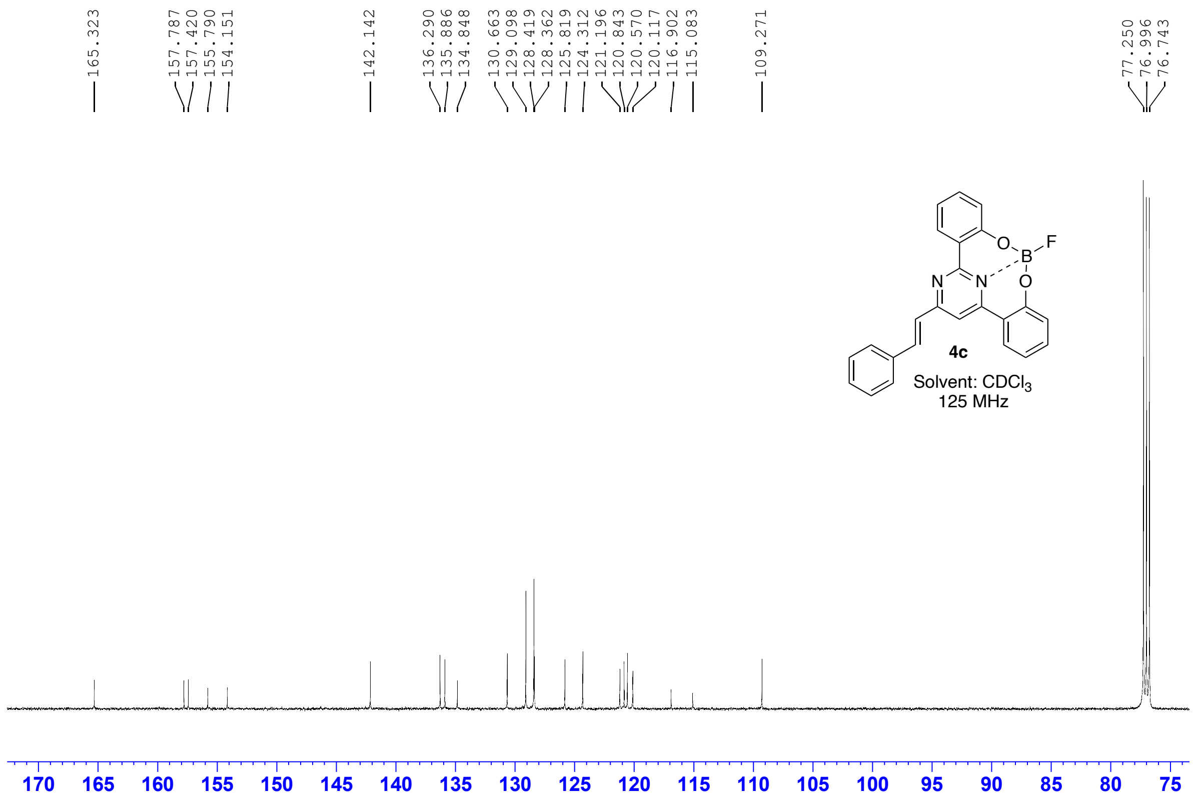

Figure S31. ^{1}H and ^{13}C NMR spectra of **4c**.

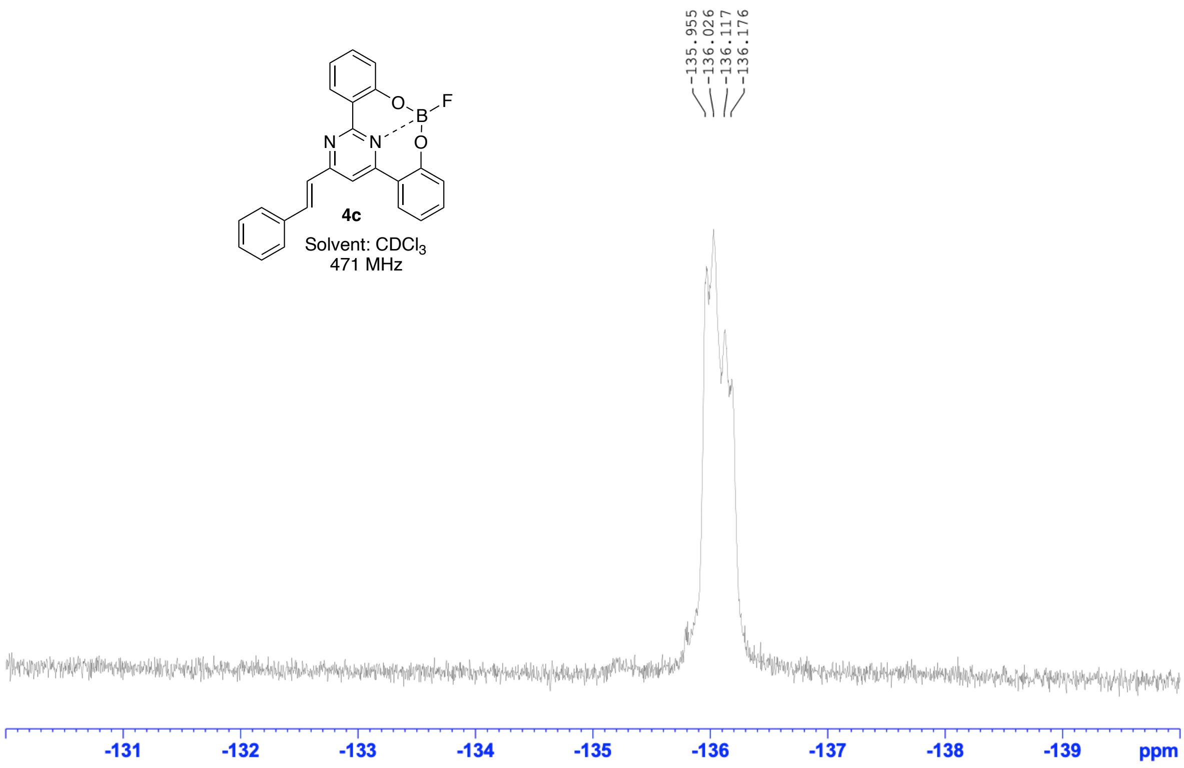

Figure S32. ^{19}F NMR spectra of **4c**.

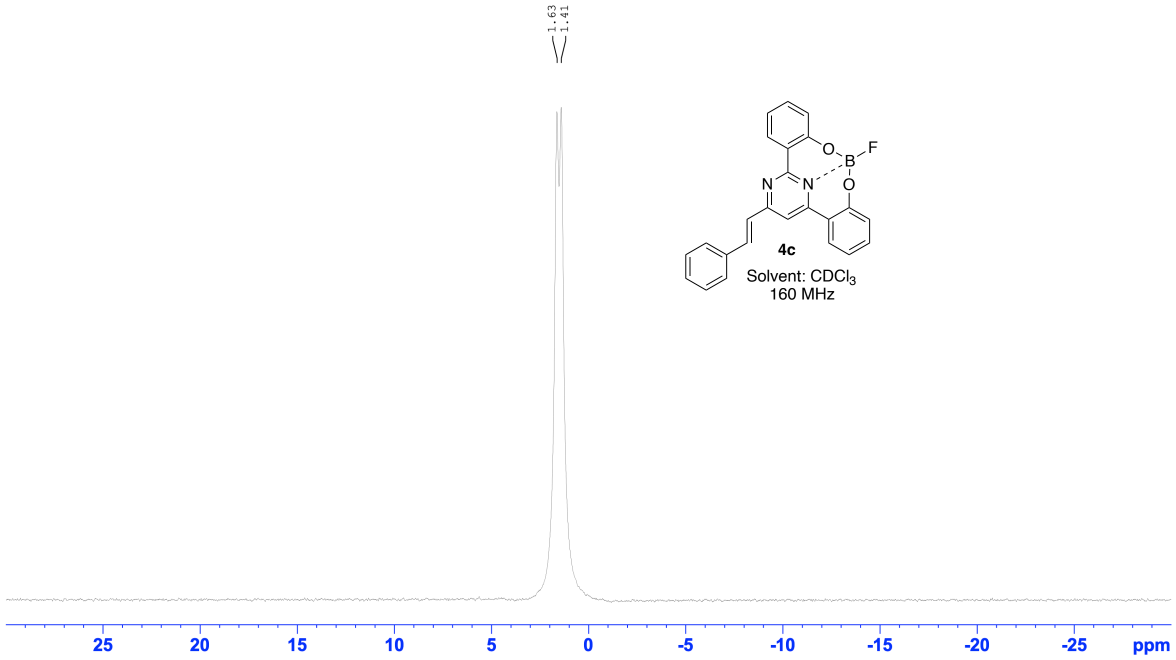

Figure S33. ^{11}B NMR spectra of **4c**.

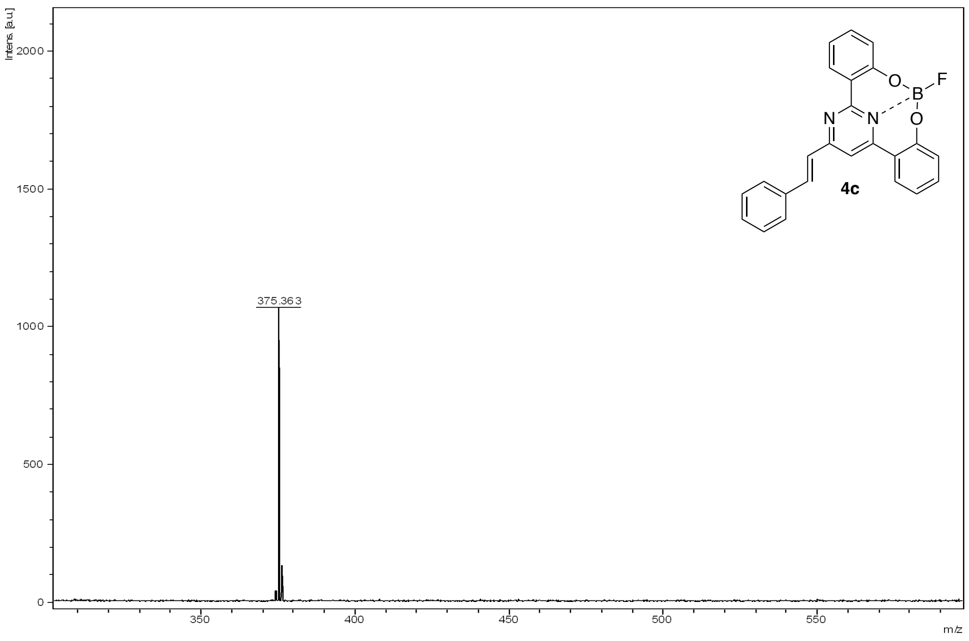

m/z	Intens.	Res.
375.363	983.70	2637

Figure S34. MALDI-TOF MS (dithranol) spectrum of **4c**.

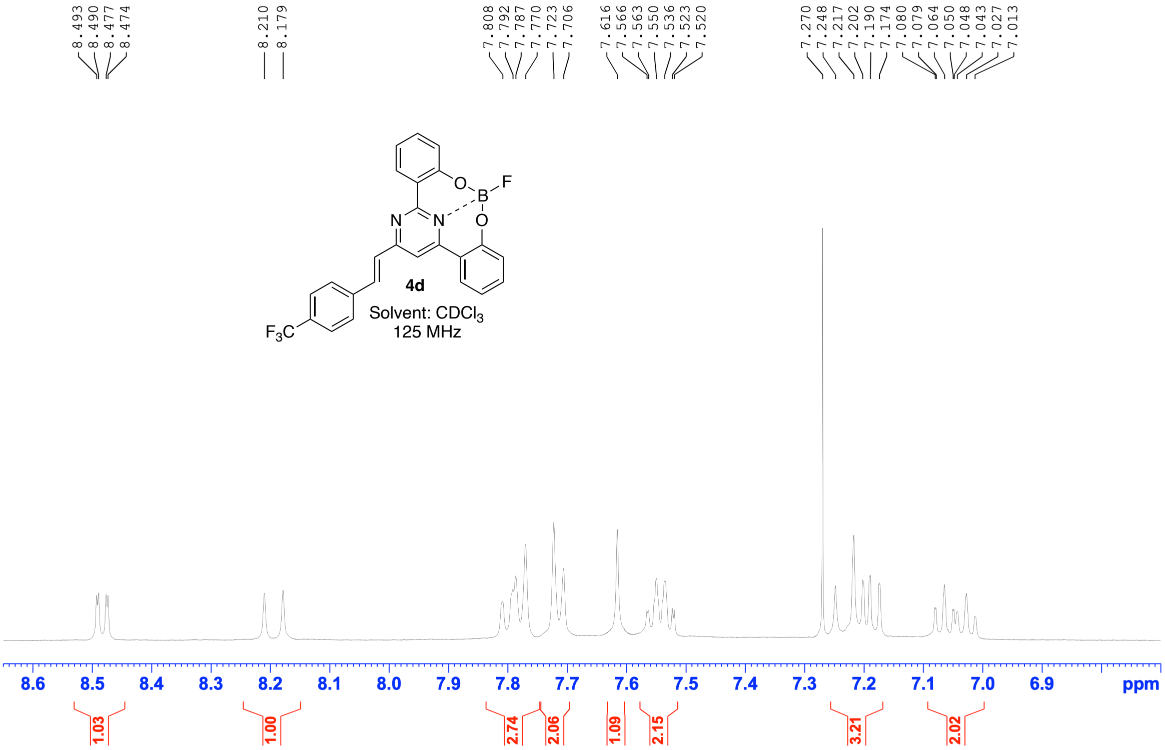

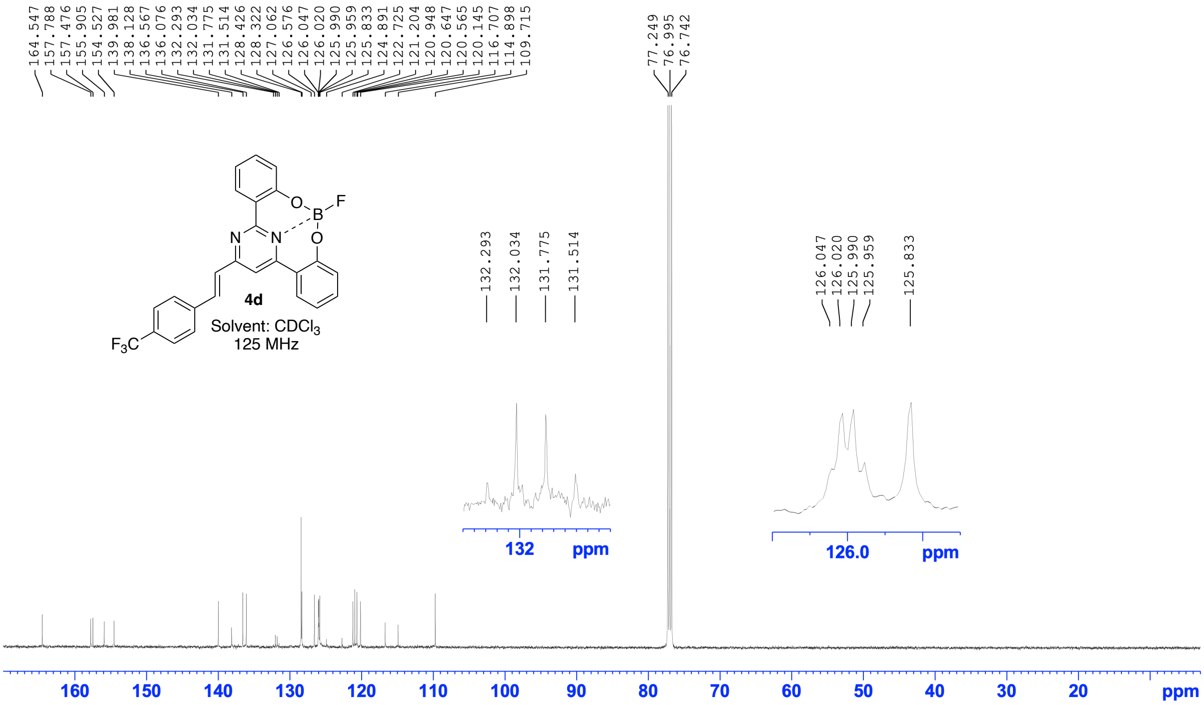

Figure S35. ^{1}H and ^{13}C NMR spectra of **4d**.

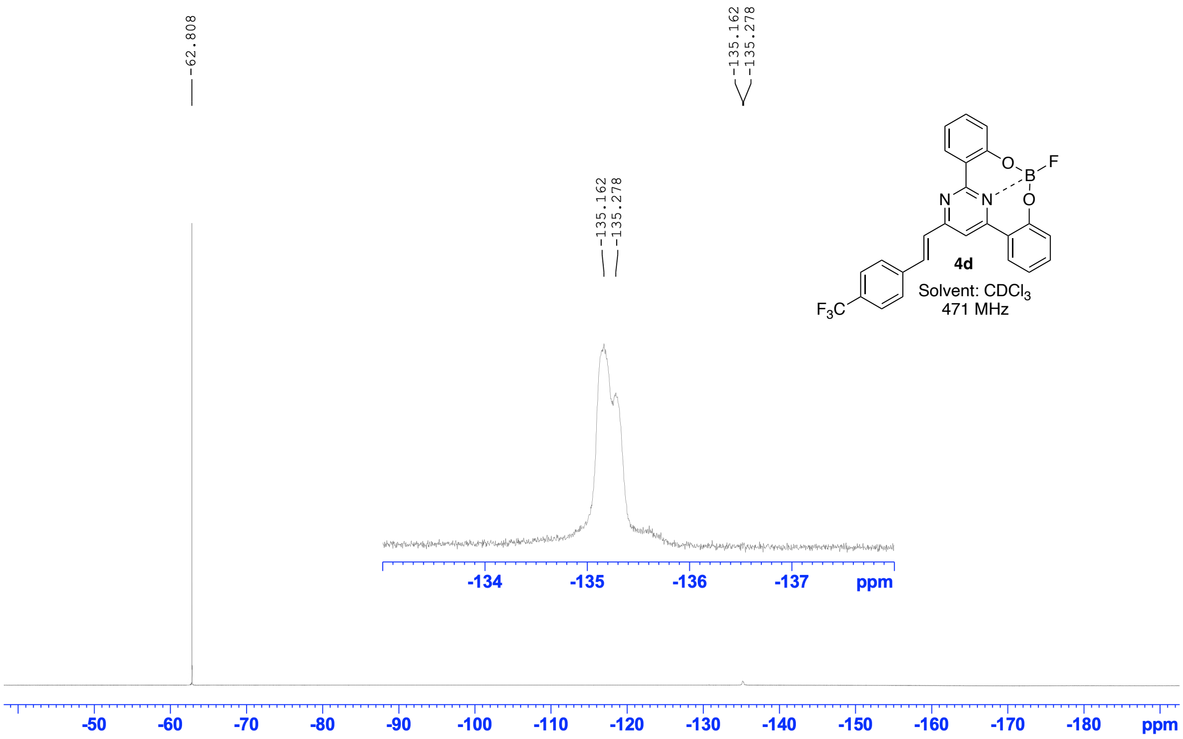

Figure S36. ^{19}F NMR spectra of **4d**.

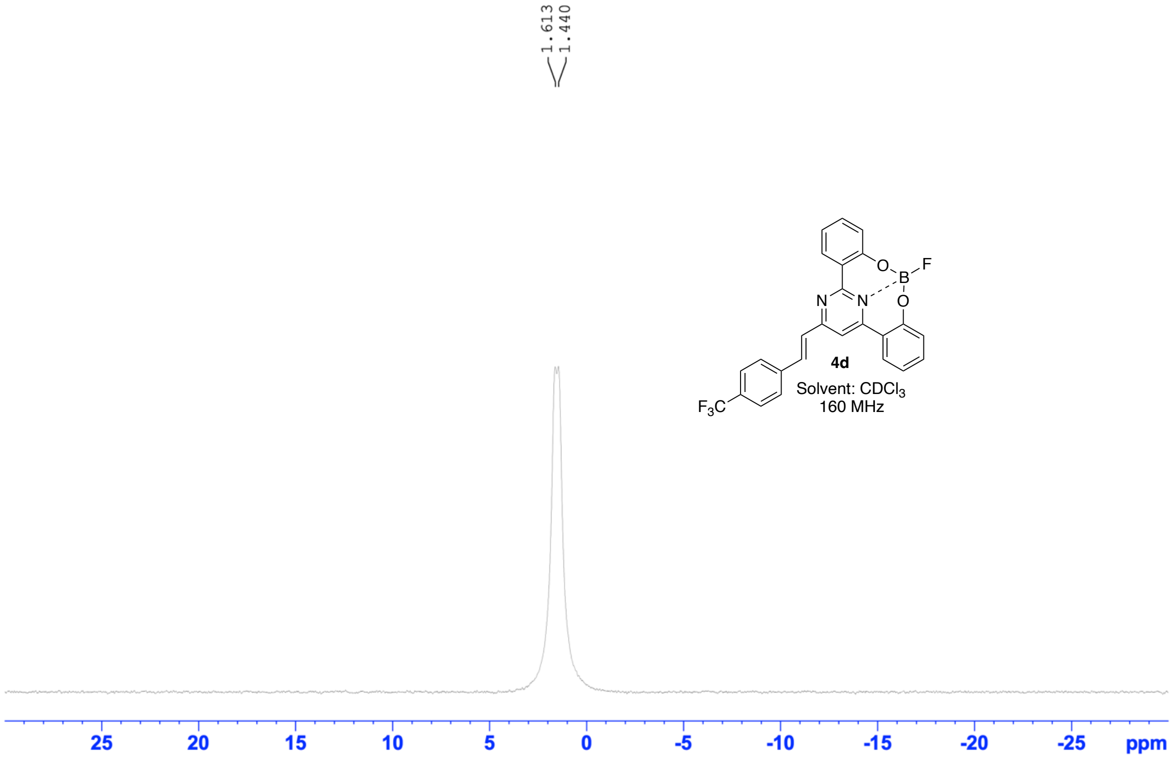

Figure S37. ^{11}B NMR spectra of **4d**.

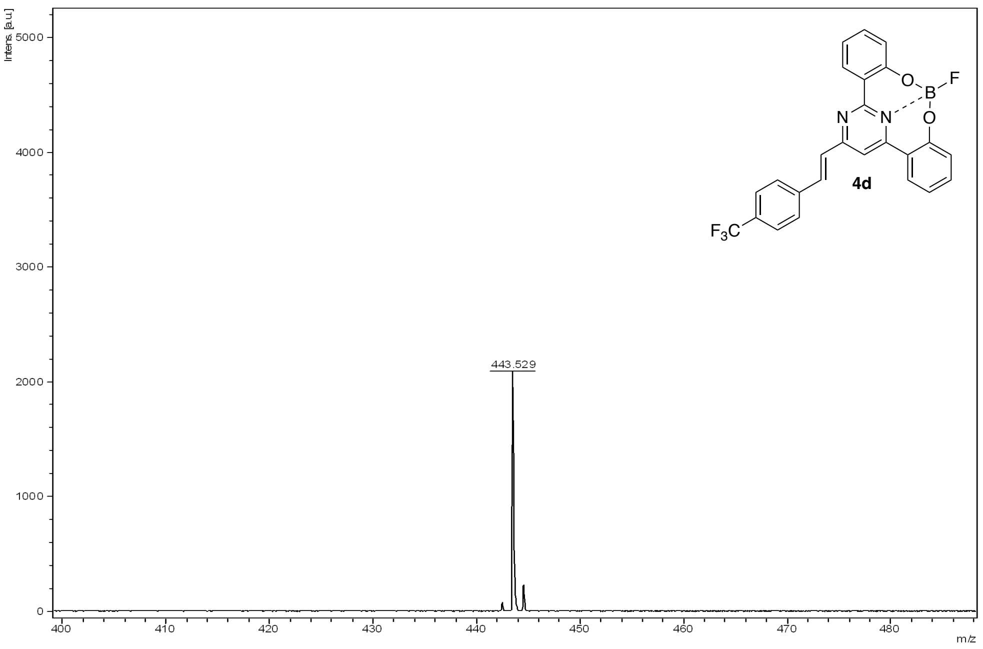

m/z	Intens.	Res.
443.529	1765.17	2933

Figure S38. MALDI-TOF MS (dithranol) spectrum of **4d**.

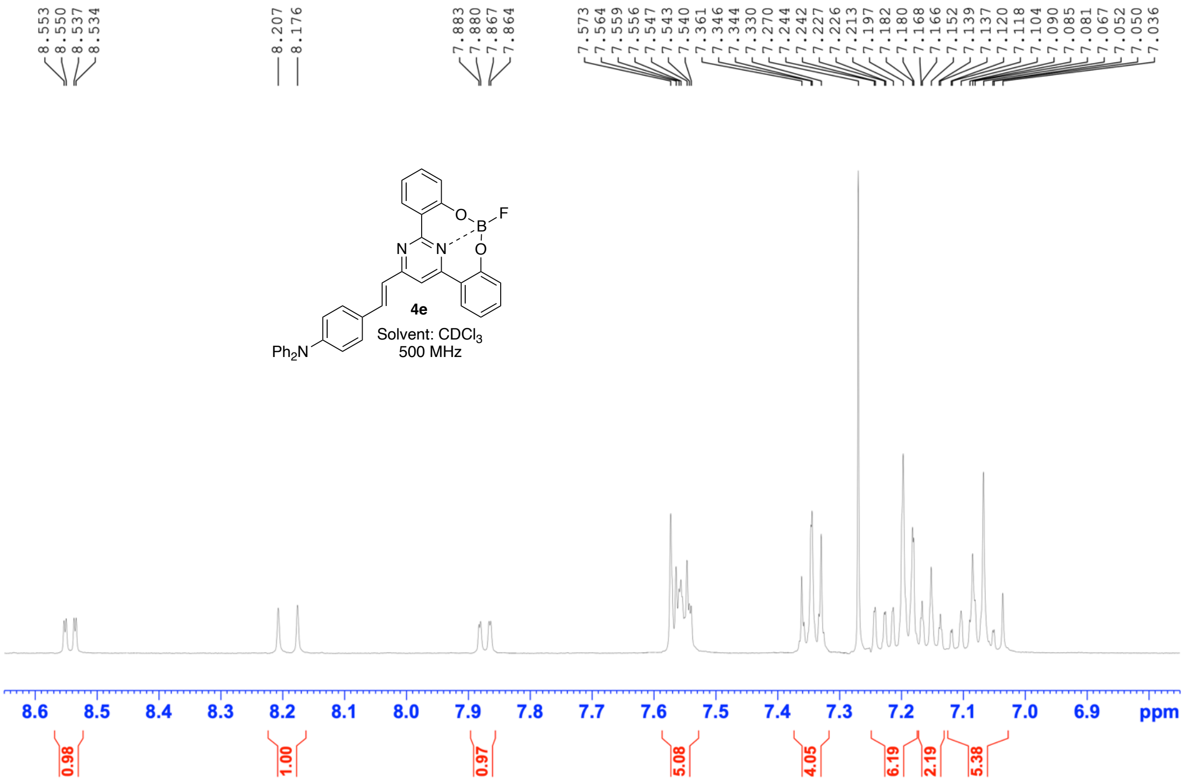

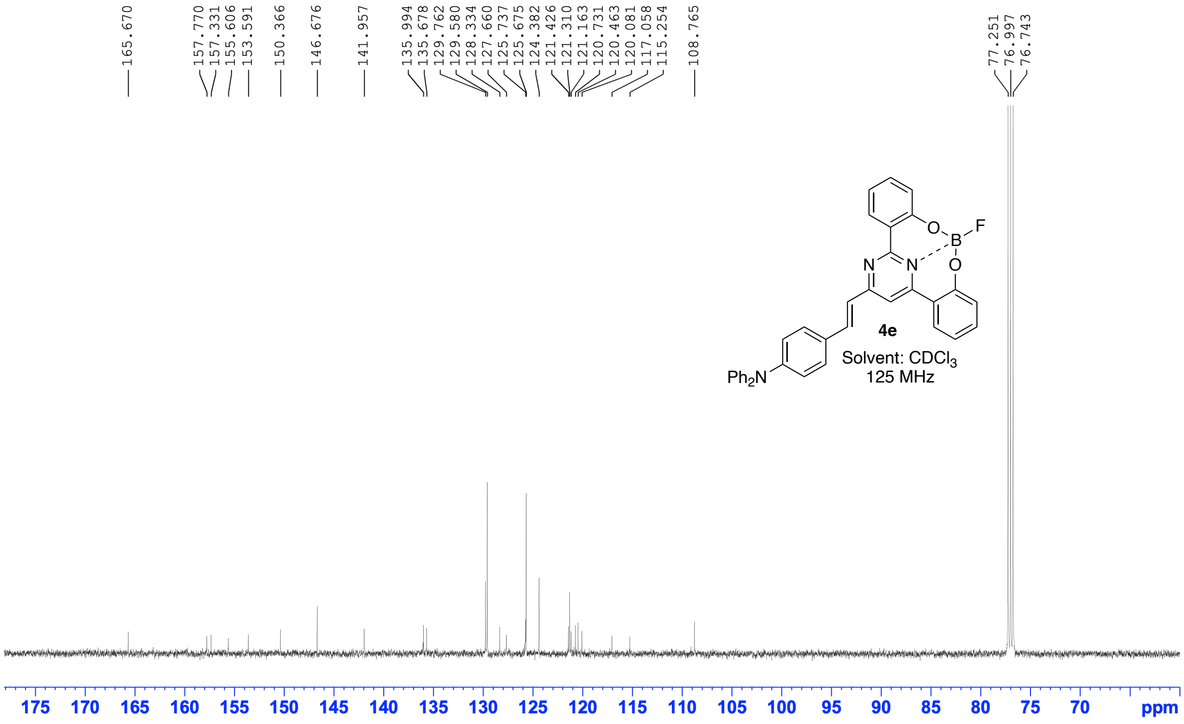

Figure S39. ^{1}H and ^{13}C NMR spectra of **4e**.

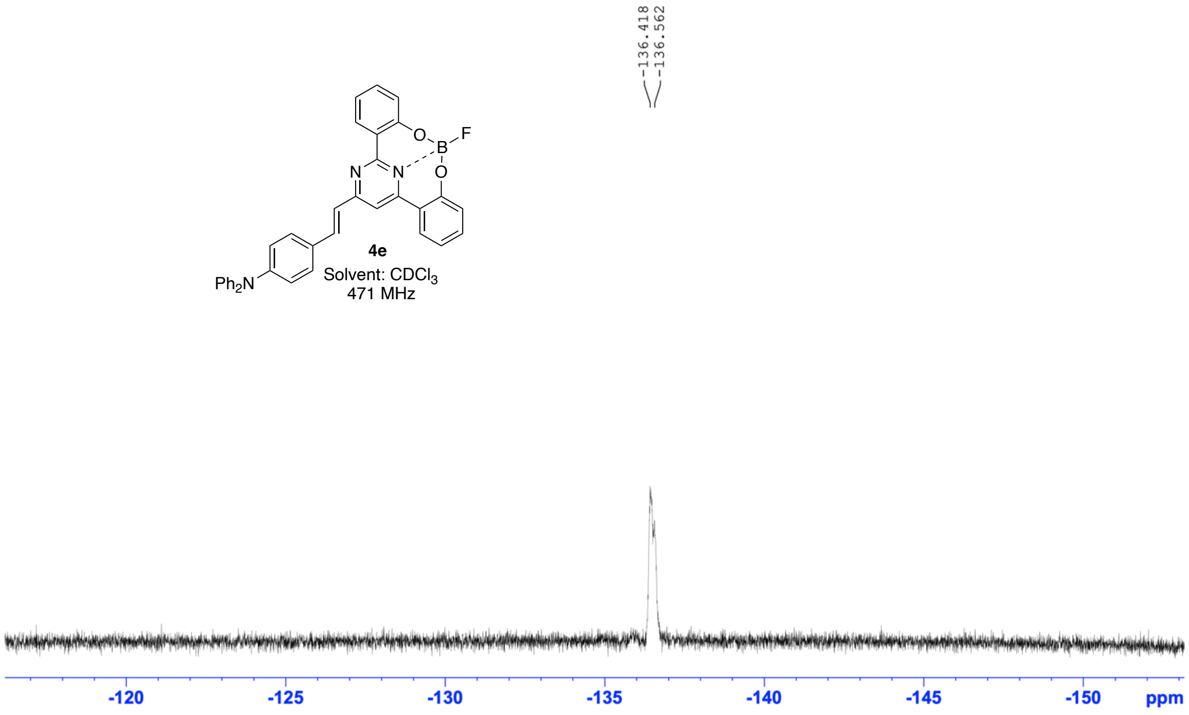

Figure S40. ^{19}F NMR spectra of **4e**.

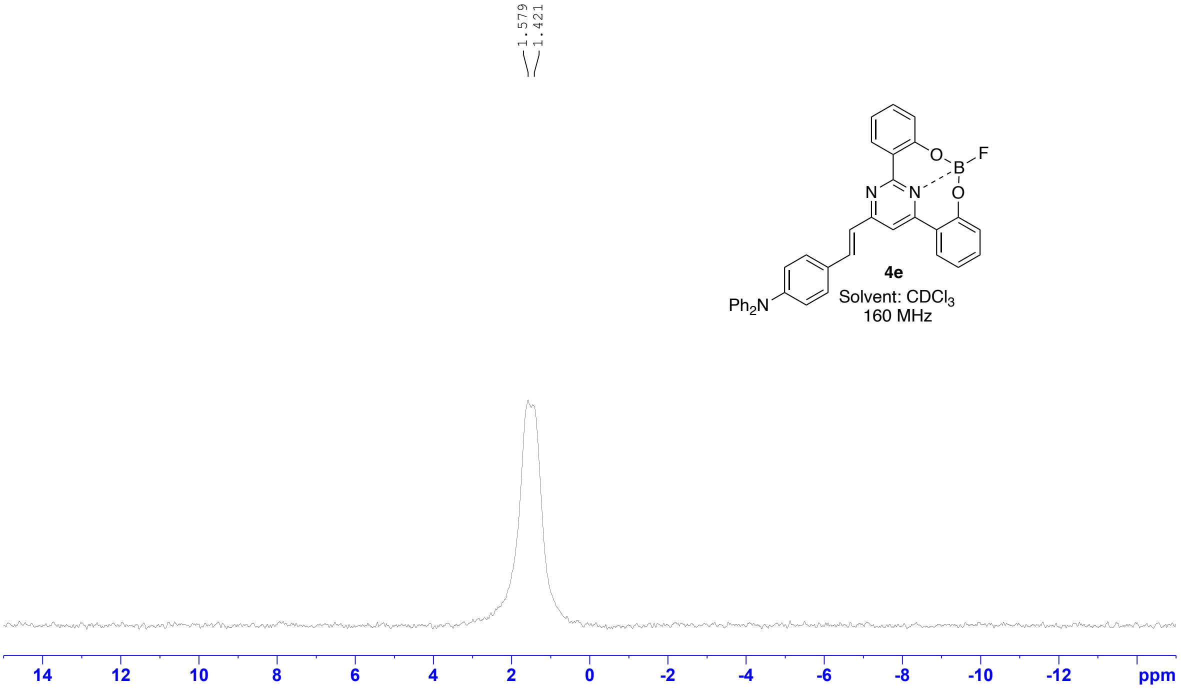

Figure S41. ^{11}B NMR spectra of **4e**.

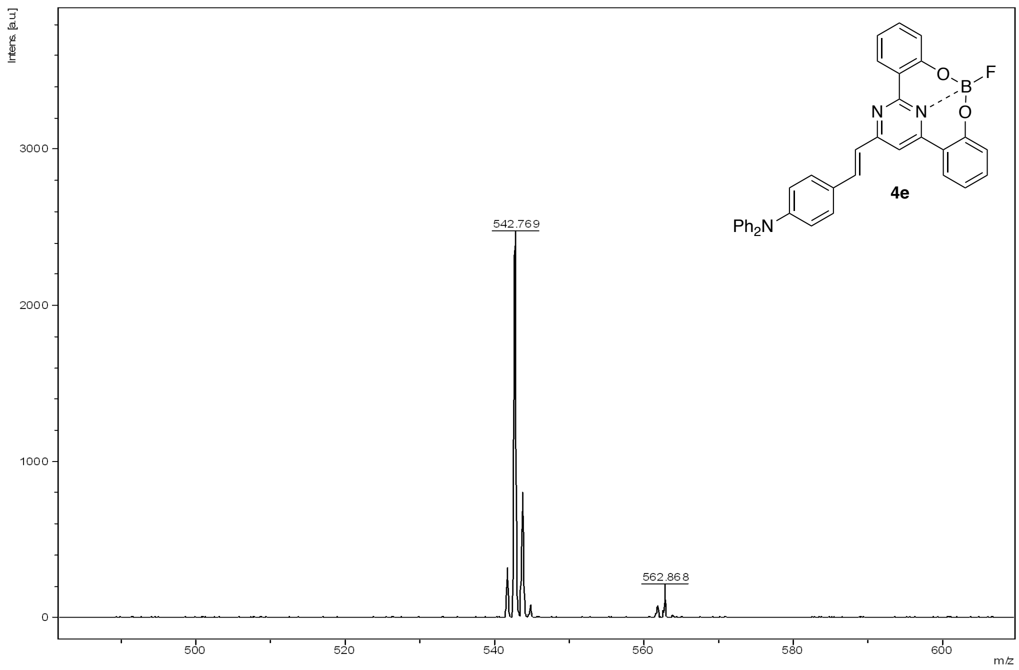

m/z	Intens.	Res.
562.868	105.07	3465
562.643	44.30	6641
561.872	62.76	2436
542.769	2266.31	2265

Figure S42. MALDI-TOF MS (dithranol) spectrum of **4e**.

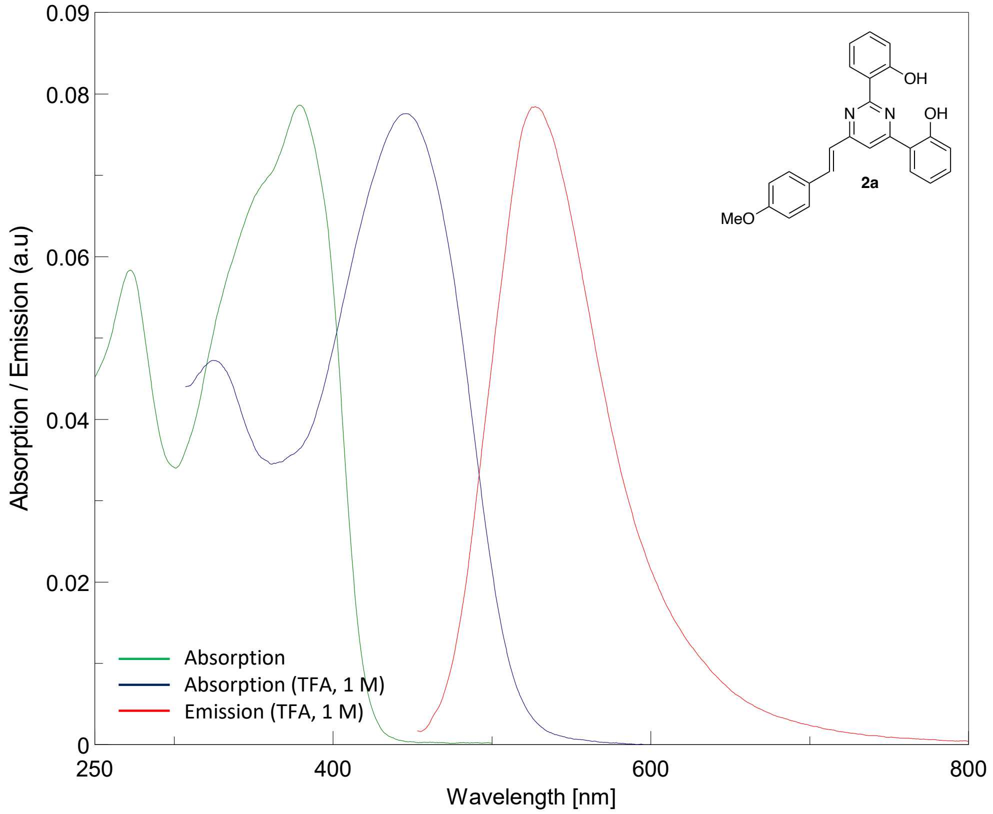

Figure S43. Absorption and emission spectra of **2a** (c = 1.74 × 10^{-6} M in CH_2Cl_2).

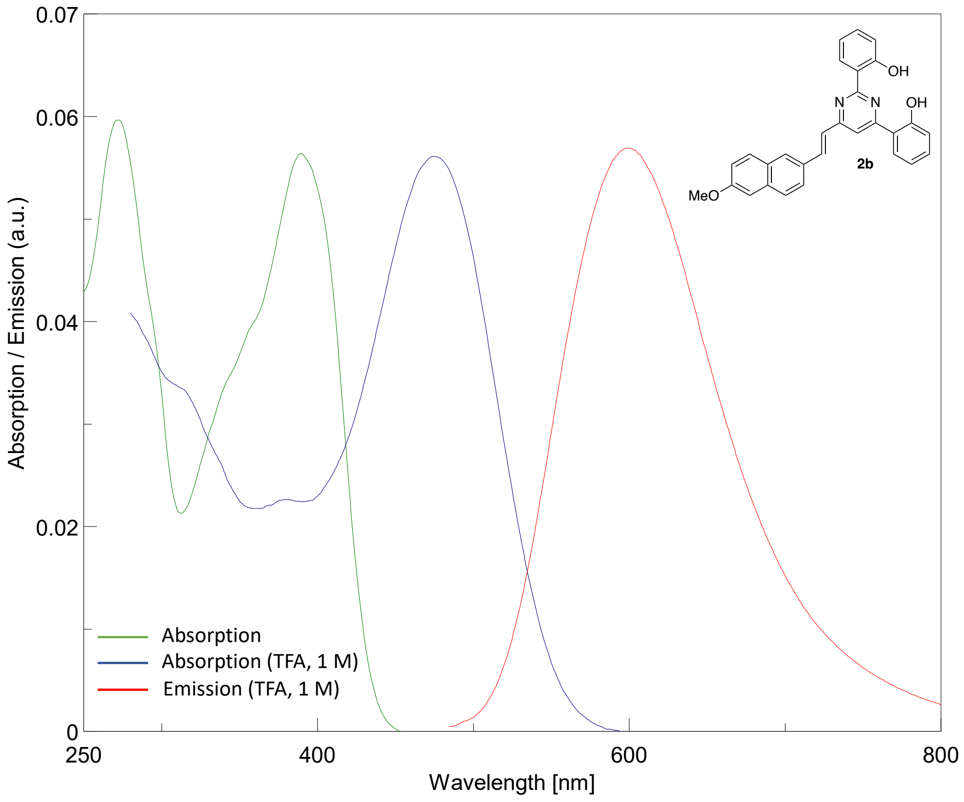

Figure S44. Absorption and emission spectra of **2b** (c = 1.23 × 10^{-6} M in CH_2Cl_2).

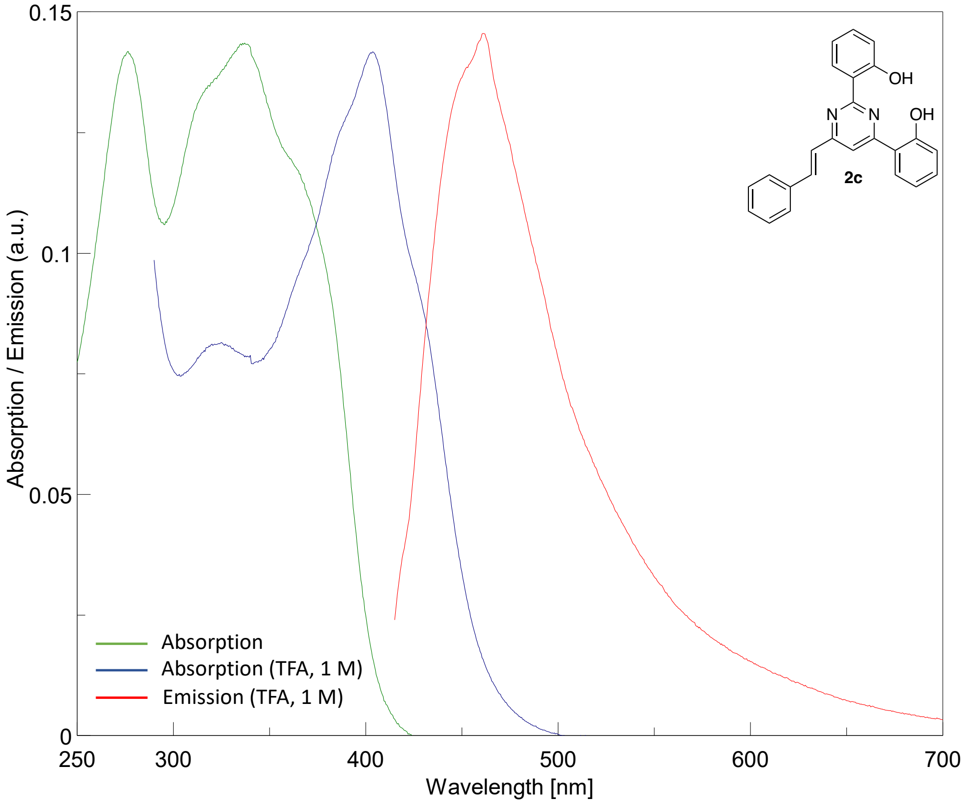

Figure S45. Absorption and emission spectra of **2c** (c = 2.70 × 10^{-6} M in CH_2Cl_2).

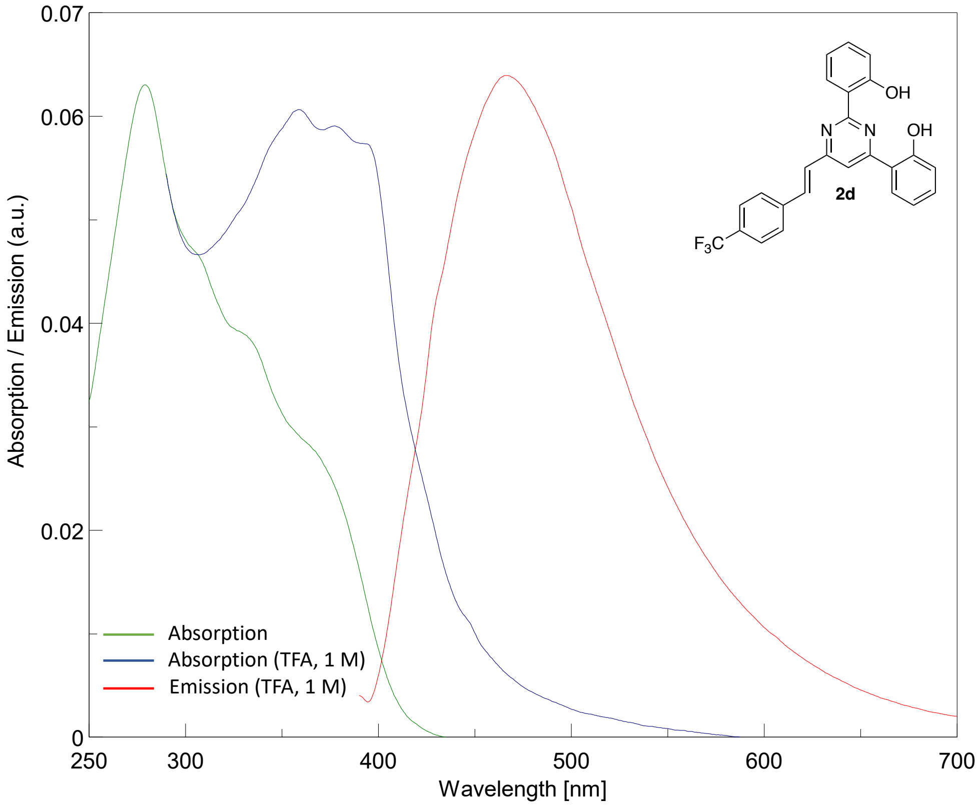

Figure S46. Absorption and emission spectra of **2d** (c = 1.45 × 10^{-6} M in CH_2Cl_2).

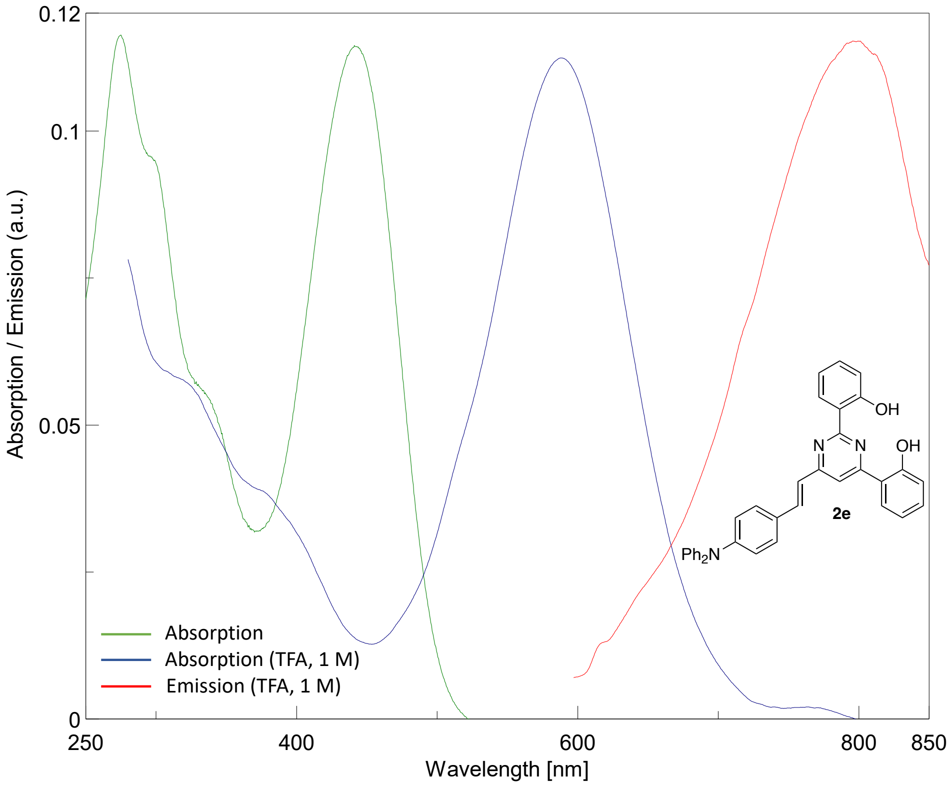

Figure S47. Absorption and emission spectra of **2e** (c = 1.46 × 10^{-6} M in CH_2Cl_2).

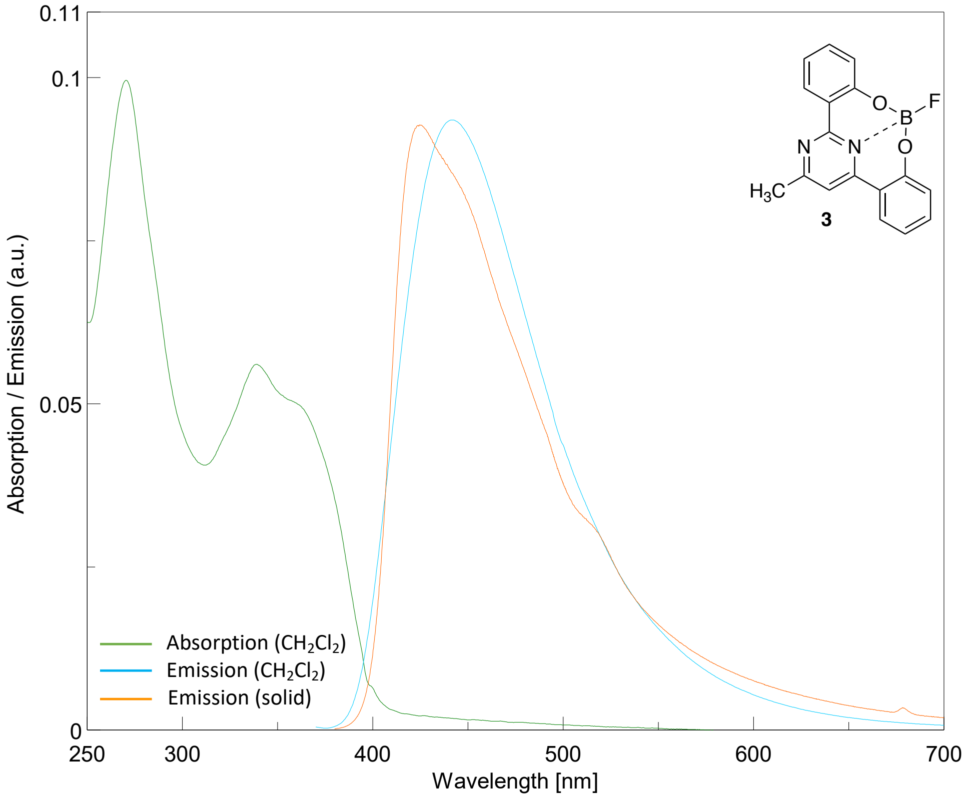

Figure S48. Absorption and emission spectra of **3** in CH_2Cl_2 solution (c = 3.53 × 10^{-6} M) and emission in the solid state.

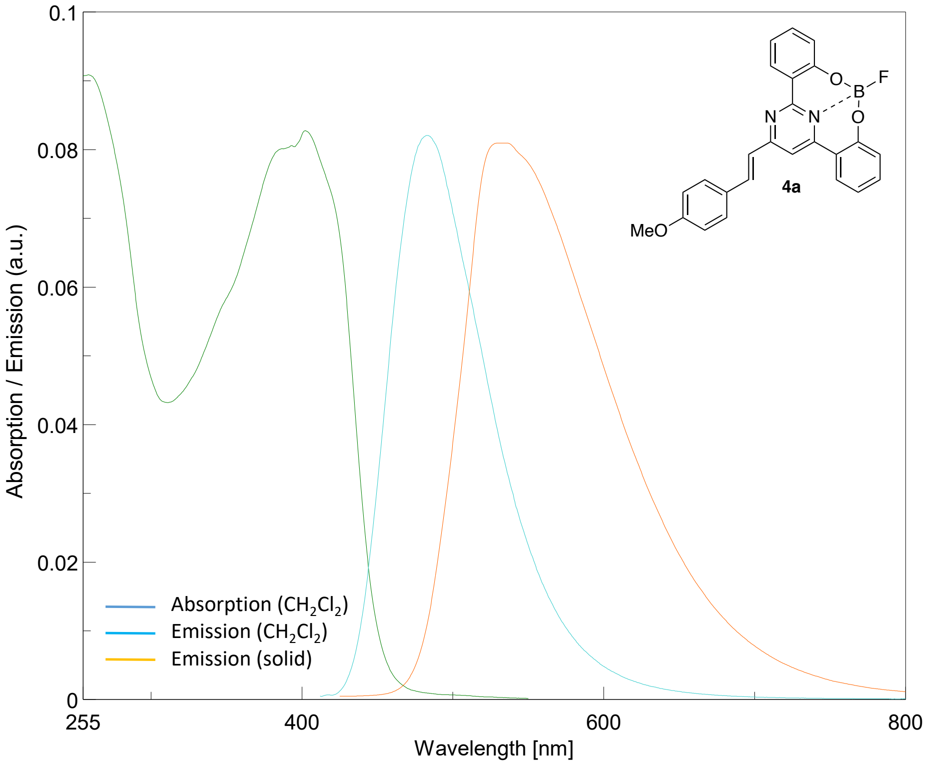

Figure S49. Absorption and emission spectra of **4a** in CH_2Cl_2 solution (c = 3.30 × 10^{-6} M) and emission in the solid state.

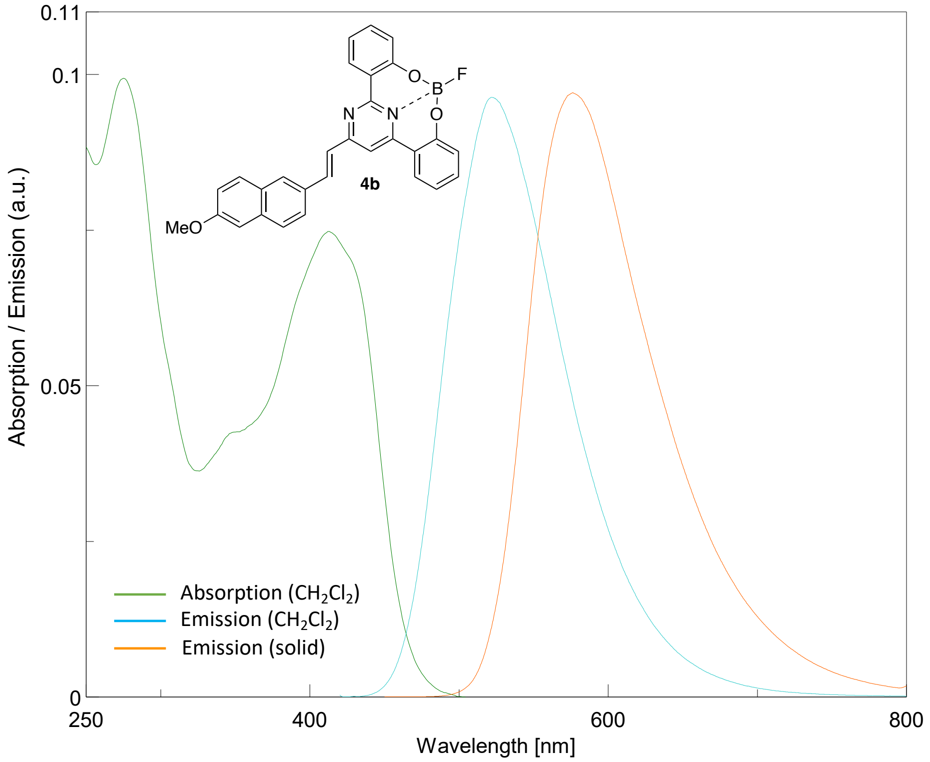

Figure S50. Absorption and emission spectra of **4b** in CH_2Cl_2 solution ($c = 2.02 \times 10^{-6}$ M) and emission in the solid state.

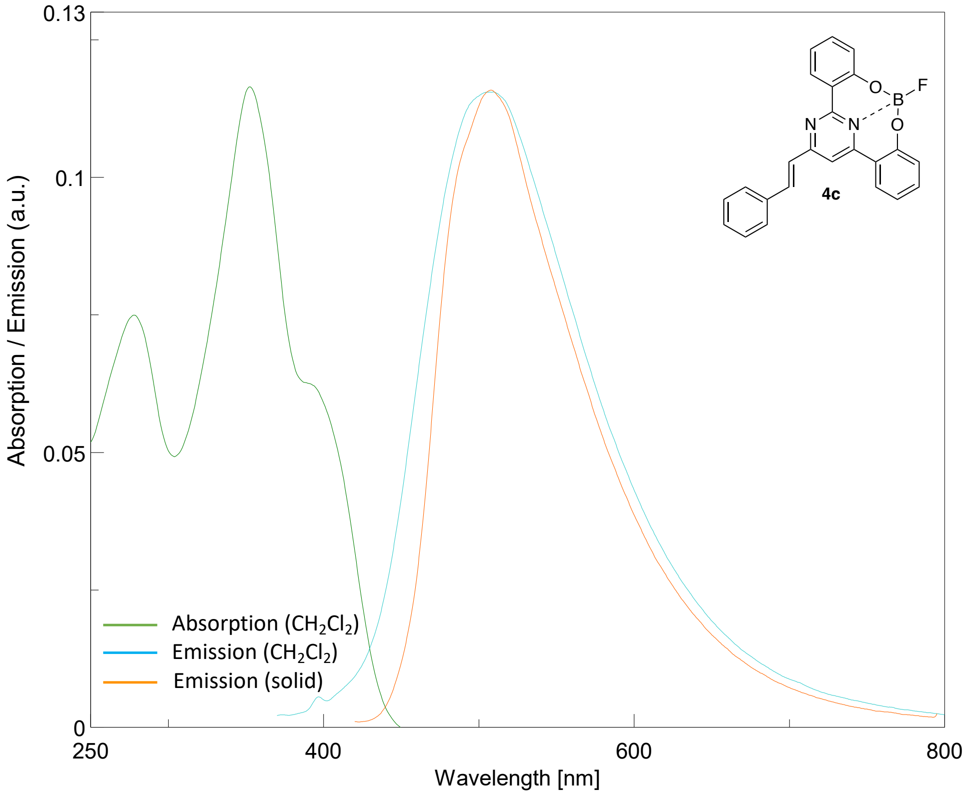

Figure S51. Absorption and emission spectra of **4c** in CH_2Cl_2 solution ($c = 2.64 \times 10^{-6}$ M) and emission in the solid state.

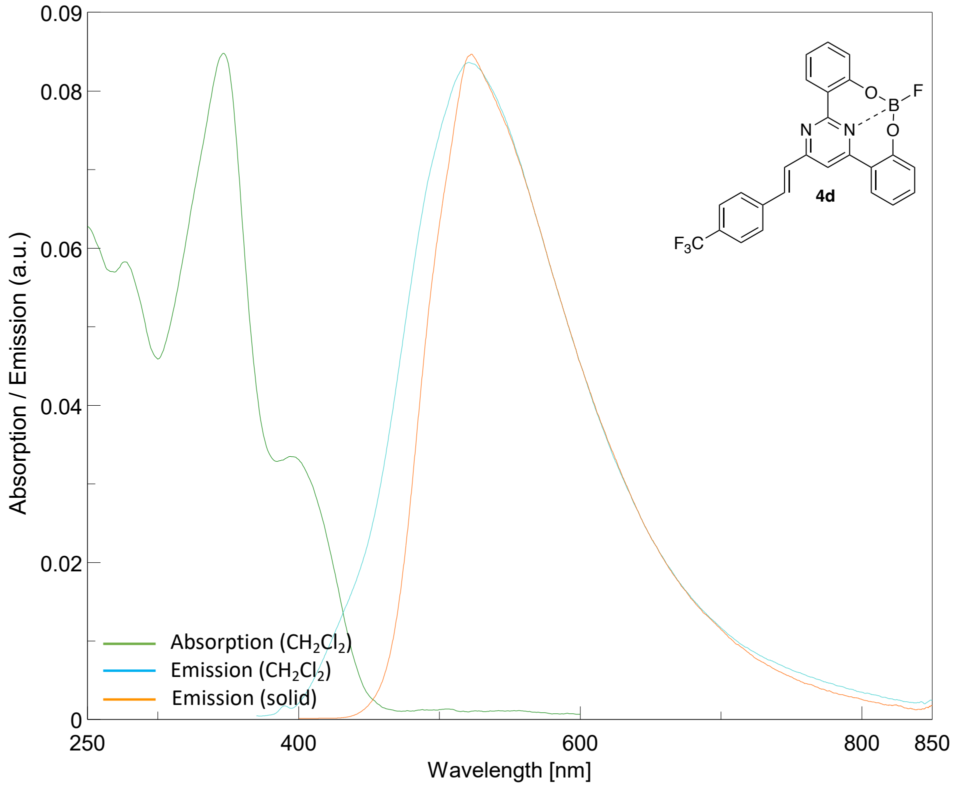

Figure S52. Absorption and emission spectra of **4d** in CH_2Cl_2 solution (c = 4.50 × 10^{-6} M) and emission in the solid state.

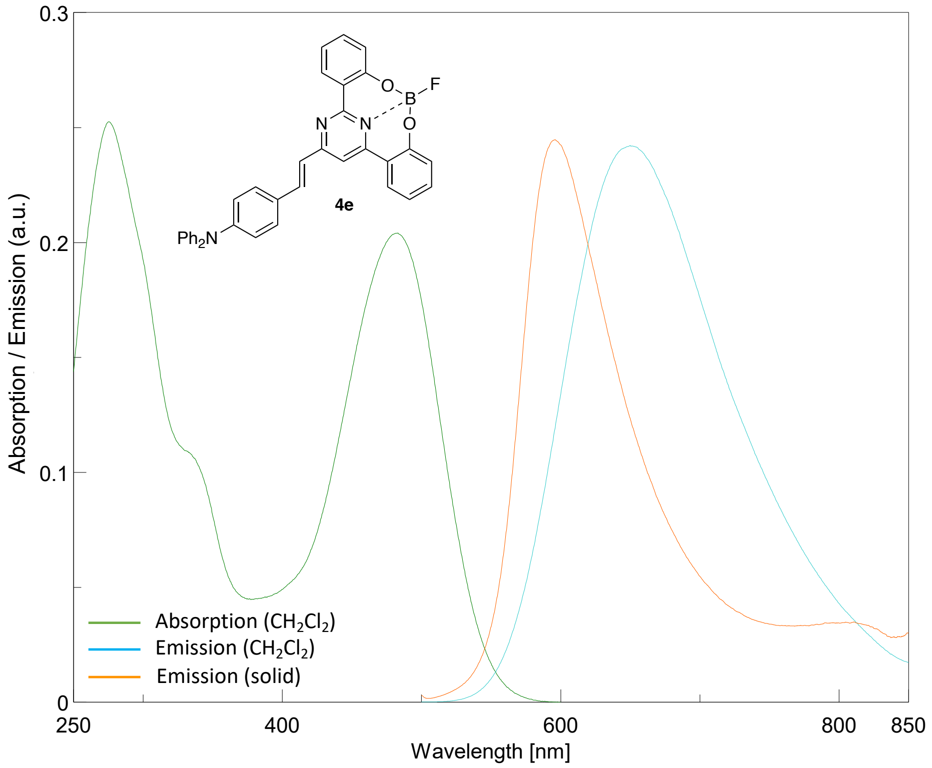

Figure S53. Absorption and emission spectra of **4e** in CH_2Cl_2 solution (c = 4.27 × 10^{-6} M) and emission in the solid state.

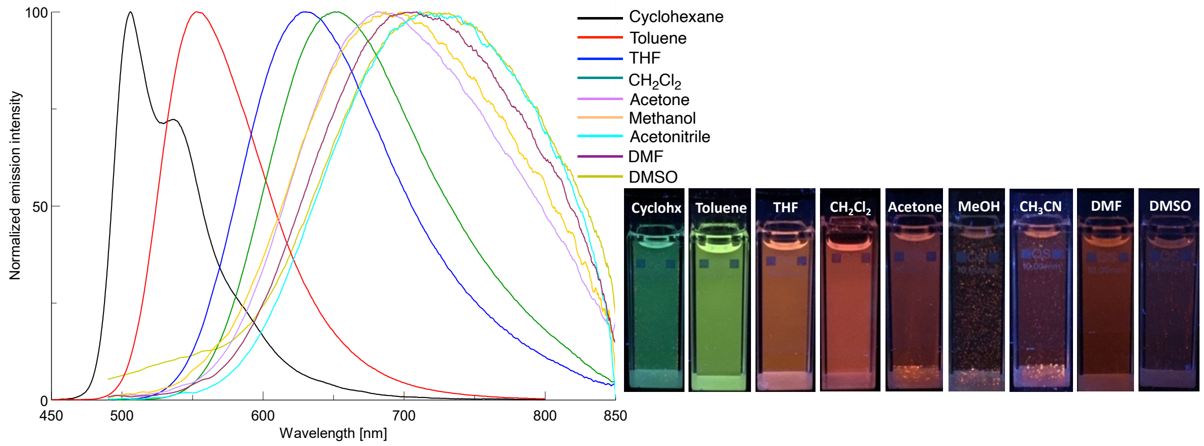

Figure S54. Left: normalized emission spectra of **4e** in various solvents. Right: color photographs under 365 nm UV light.

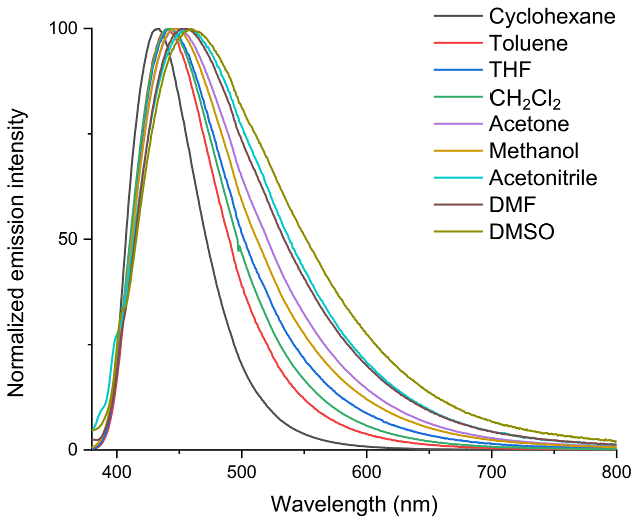

Figure S55. Normalized emission spectra of **3** in various solvents.

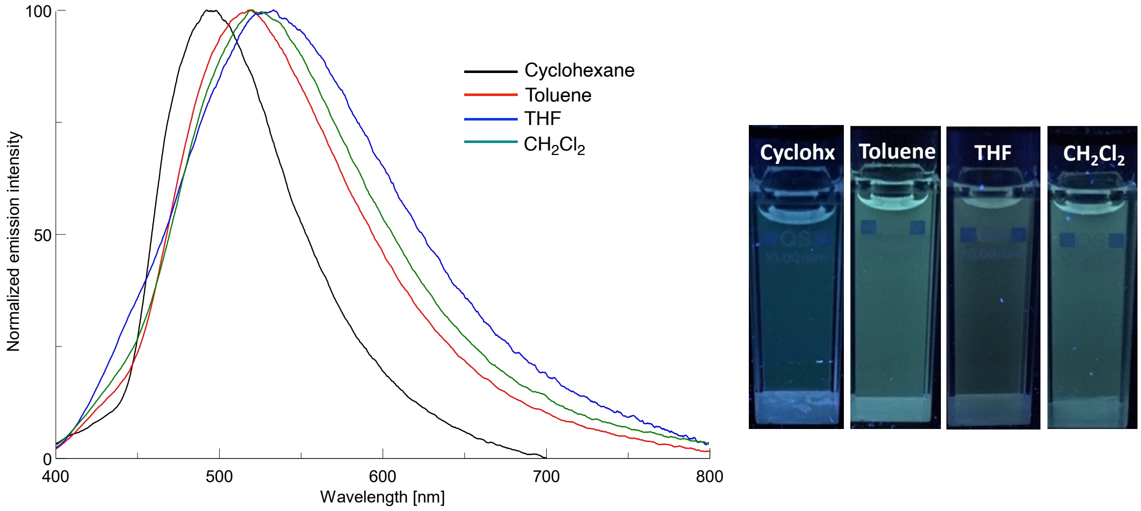

Figure S56. Left: normalized emission spectra of **4d** in various solvents. Right: color photographs under 365 nm UV light.

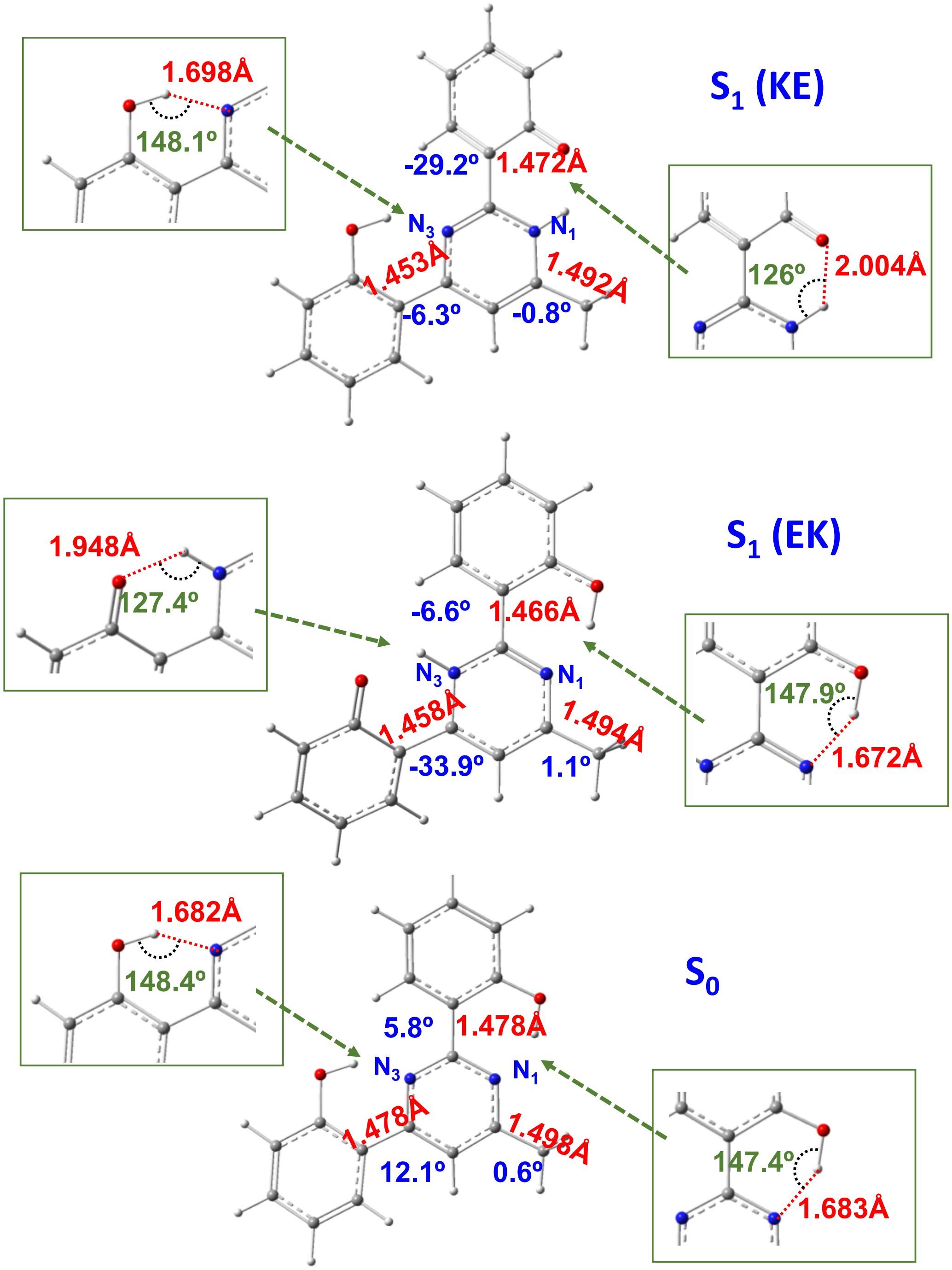

Figure S57. Selected bond lengths and dihedral angles in the S_0 and S_1 states for compound **1** calculated in CH_2Cl_2 at the M06-2X/6-31+G** level of theory.

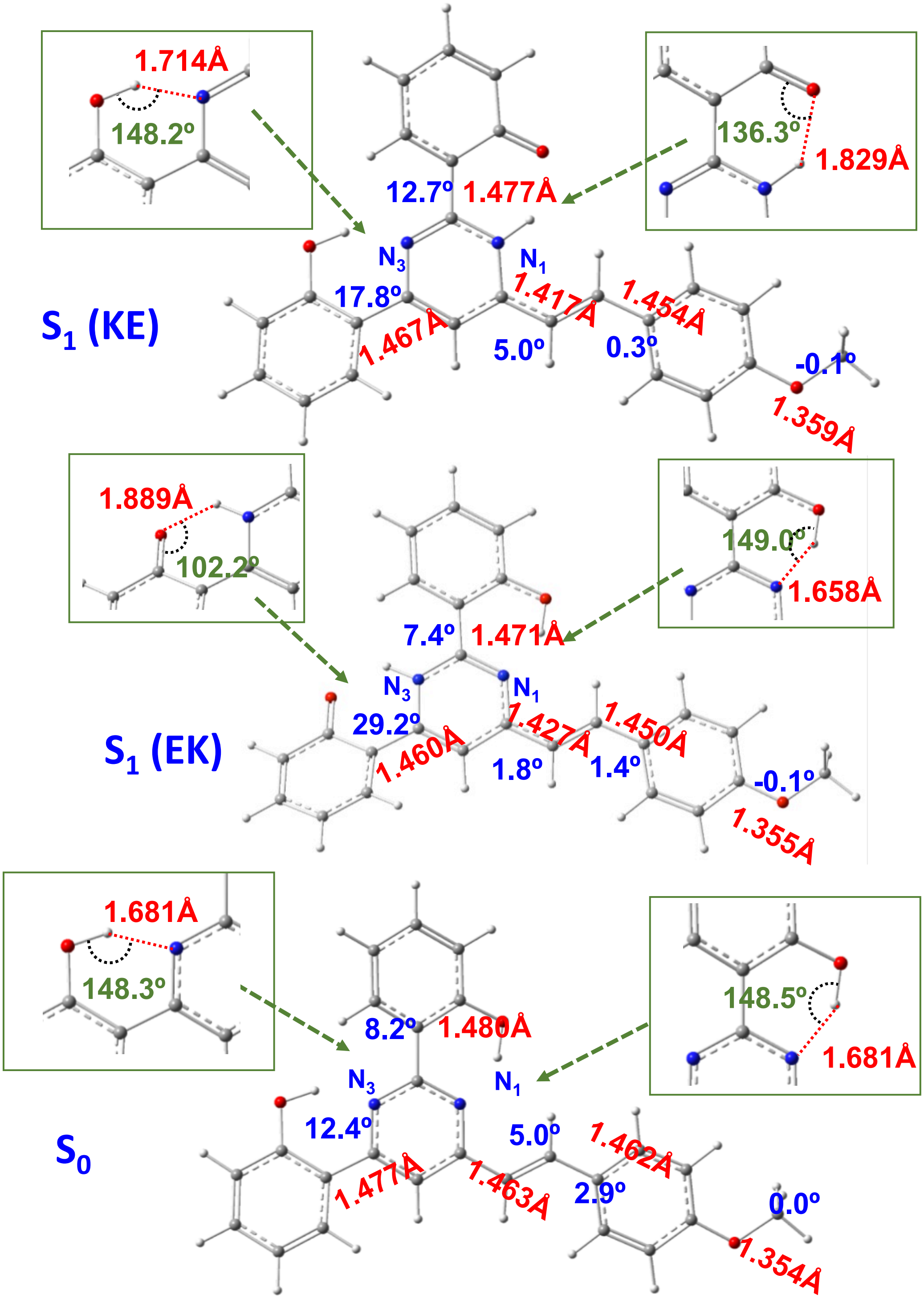

Figure S58. Selected bond lengths and dihedral angles in the S_0 and S_1 states for compound **2a** calculated in CH_2Cl_2 at the M06-2X/6-31+G** level of theory.

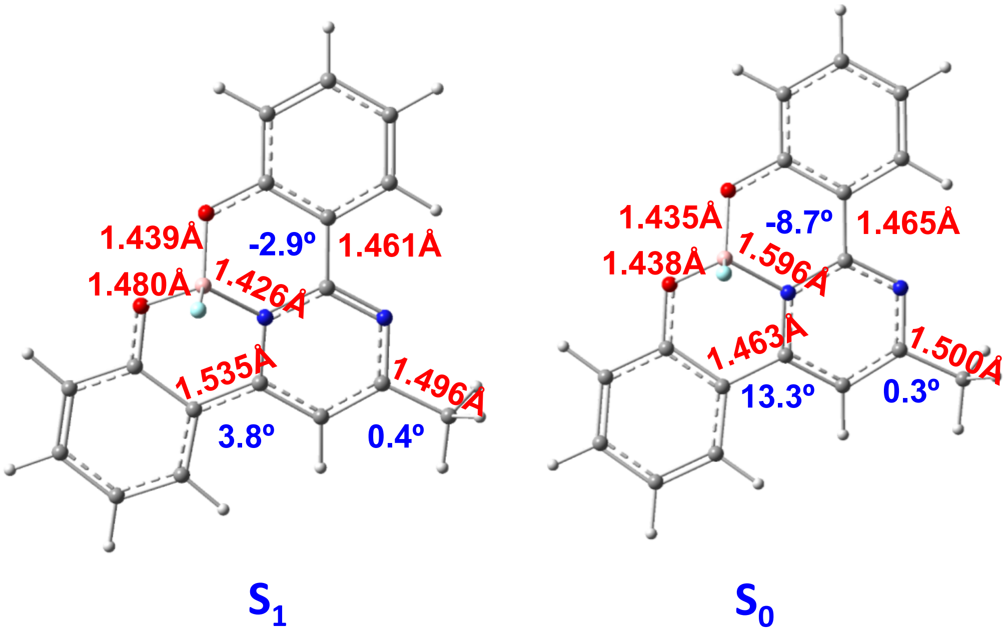

Figure S59. Selected bond lengths and dihedral angles in the S_0 and S_1 states for compound **3** calculated in CH_2Cl_2 at the M06-2X/6-31+G** level of theory.

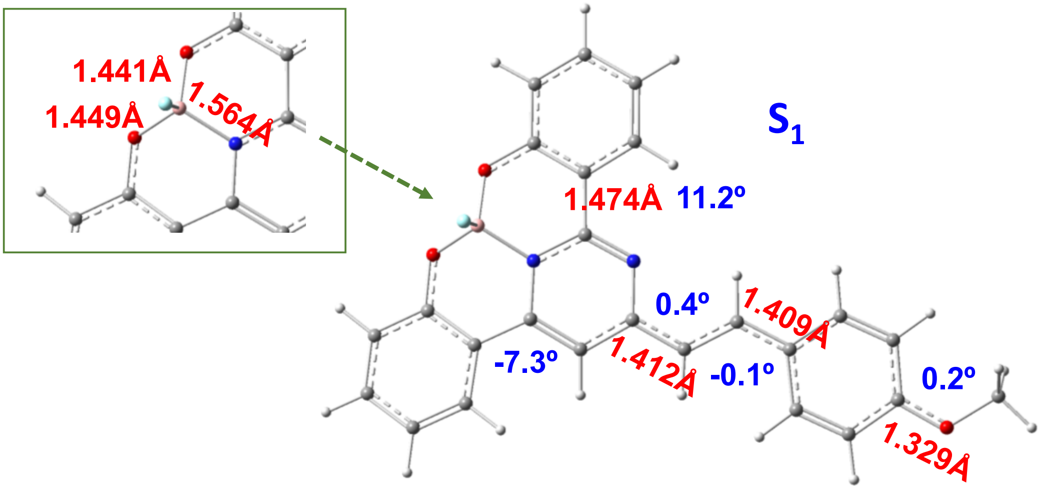

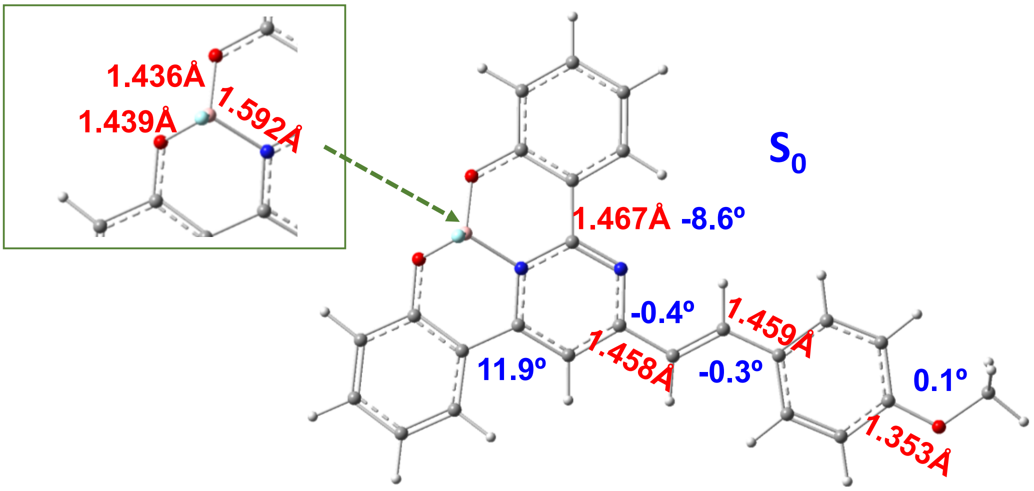

Figure S60. Selected bond lengths and dihedral angles in the S_0 and S_1 states for compound **4a** calculated in CH_2Cl_2 at the M06-2X/6-31+G** level of theory.

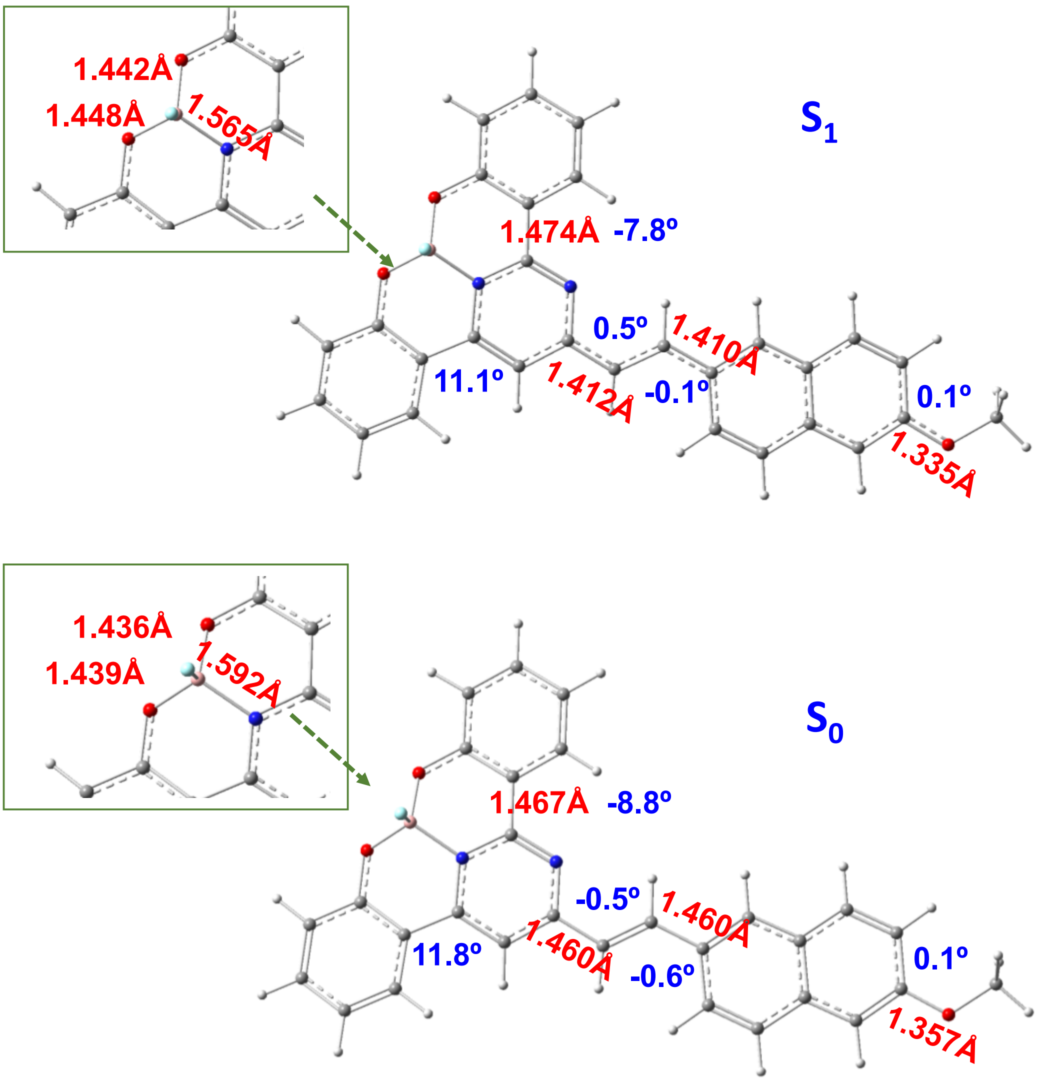

Figure S61. Selected bond lengths and dihedral angles in the S_0 and S_1 states for compound **4b** calculated in CH_2Cl_2 at the M06-2X/6-31+G** level of theory.

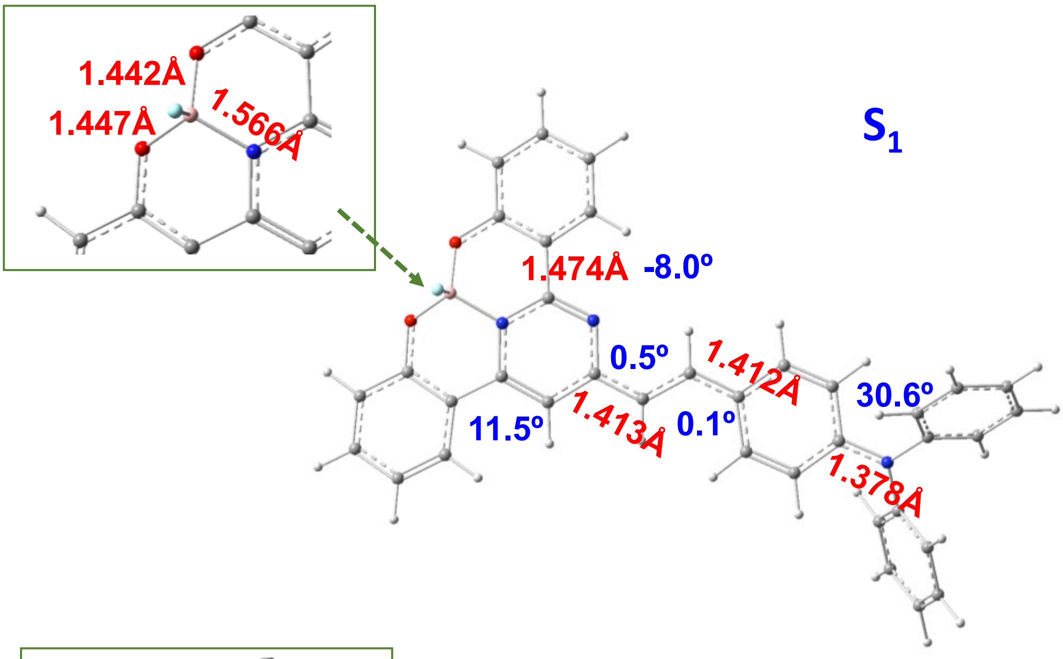

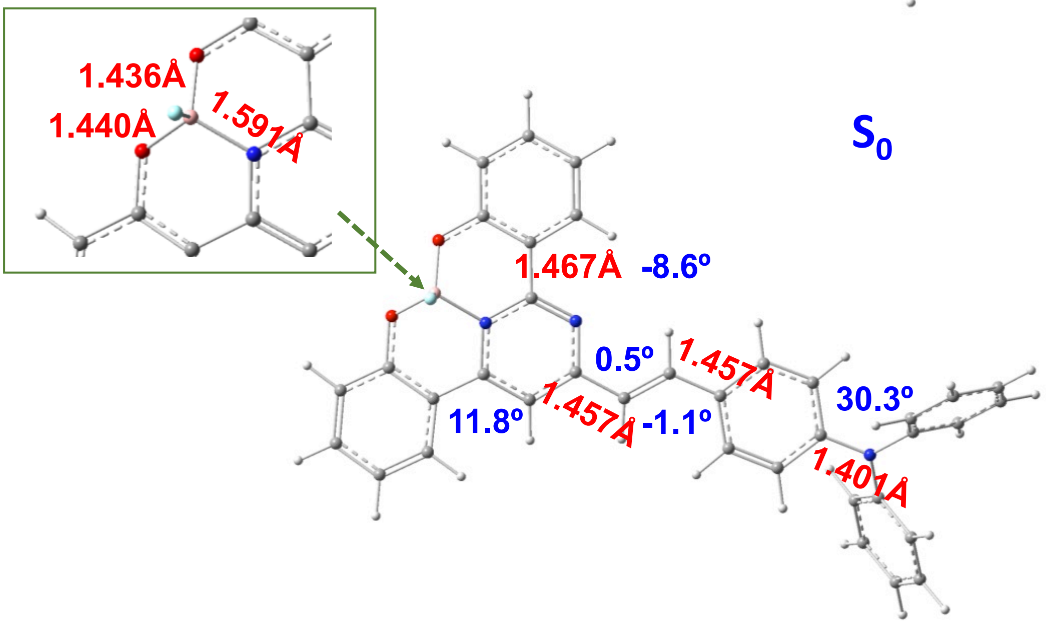

Figure S62. Selected bond lengths and dihedral angles in the S_0 and S_1 states for compound **4e** calculated in CH_2Cl_2 at the M06-2X/6-31+G** level of theory.

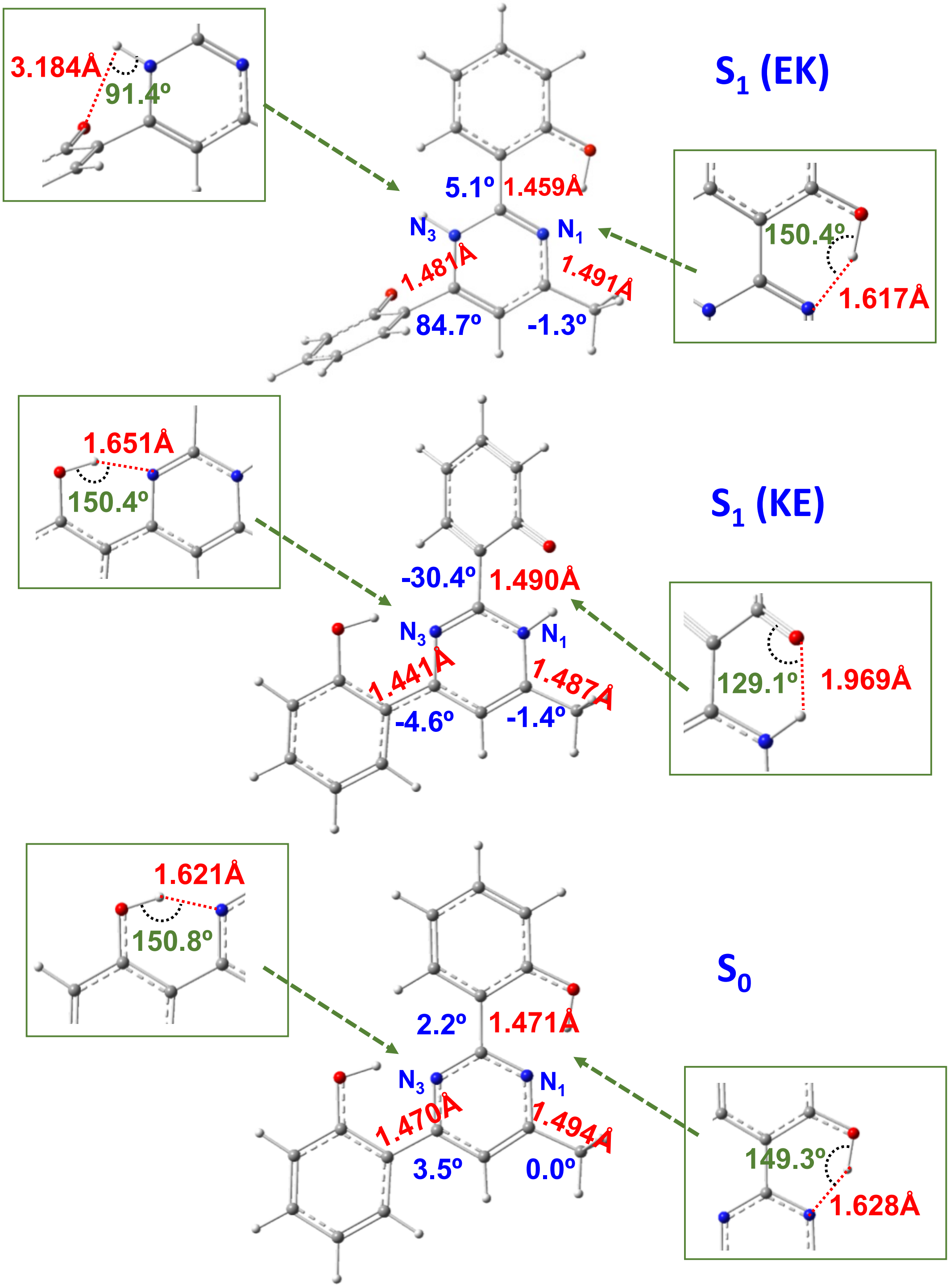

Figure S63. Selected bond lengths and dihedral angles in the S_0 and S_1 states for compound **1** calculated in CH_2Cl_2 at the PBE0/6-31+G** level of theory.

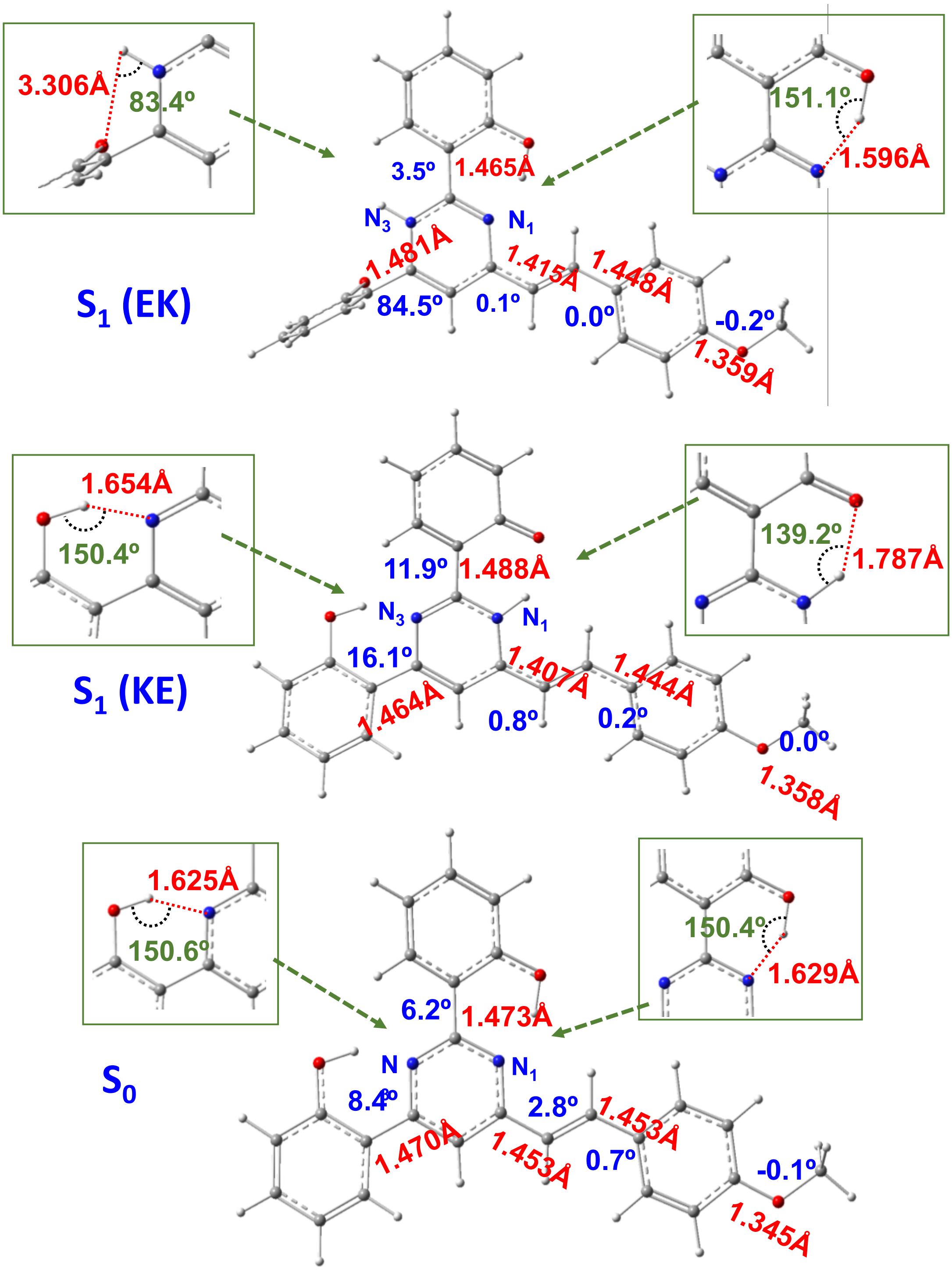

Figure S64. Selected bond lengths and dihedral angles in the S_0 and S_1 states for compound **2a** calculated in CH_2Cl_2 at the PBE0/6-31+G** level of theory.

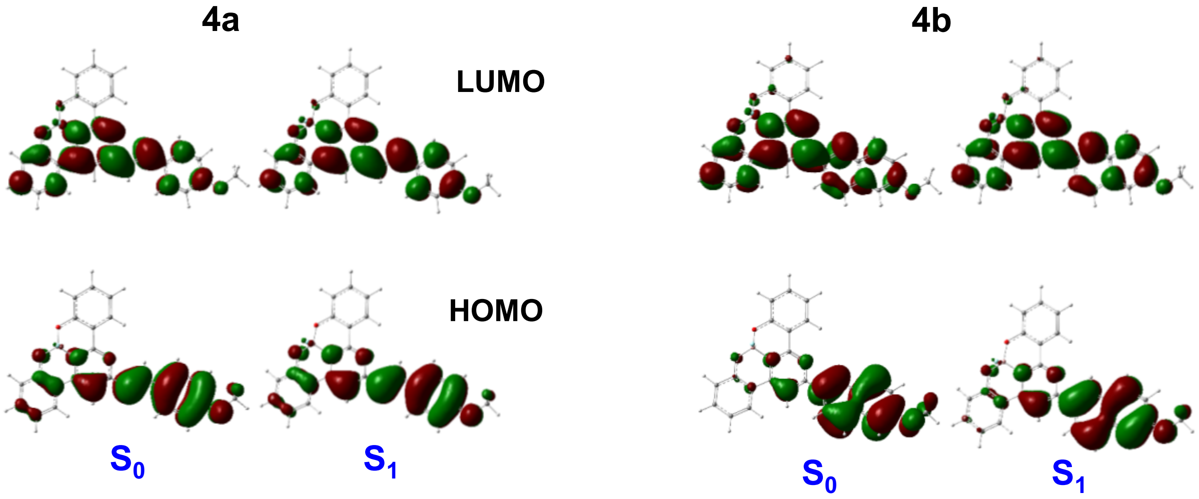

Figure S65. Molecular orbitals in CH_2Cl_2 solution calculated for the ground and excited states at the M06-2X/6-31+G** level of theory (isocontour plots 0.02 au) for compounds **4a** and **4b**.

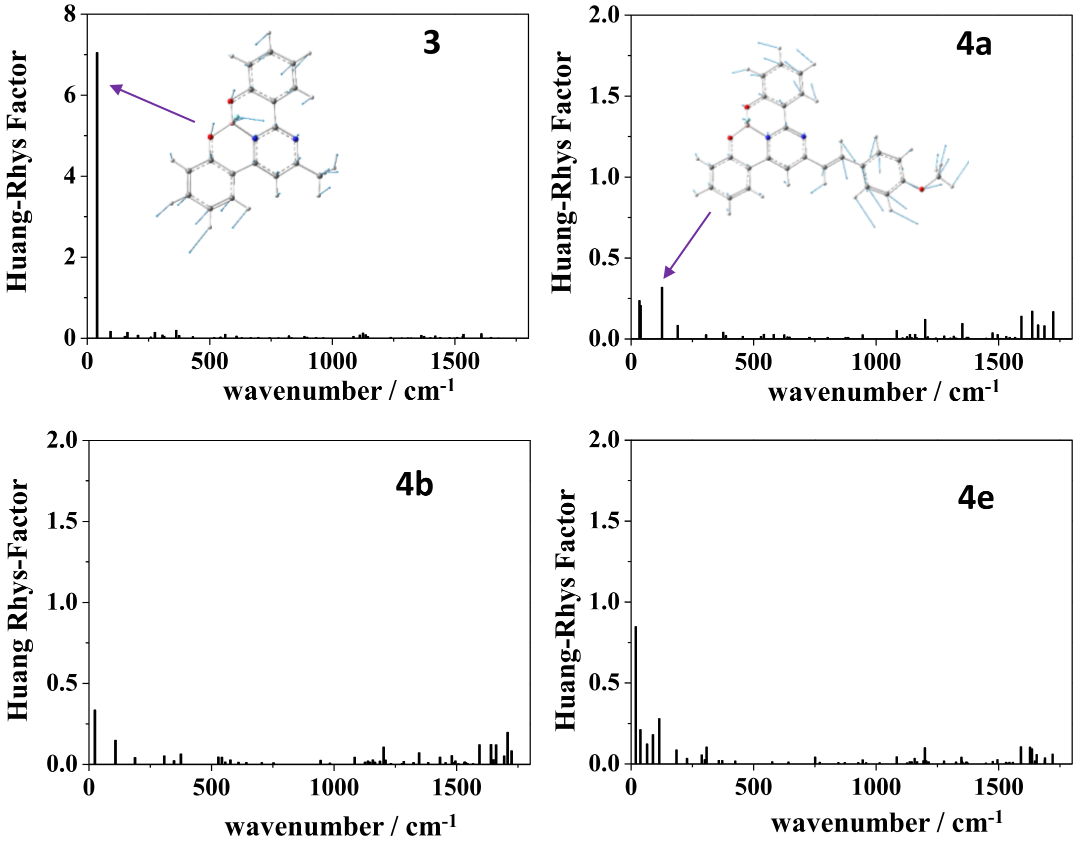

Figure S66. Huang-Rhys factors calculated for the ground state of compounds **3**, **4a**, **4b**, and **4e** in CH_2Cl_2 at the M06-2X/6-31+G** level of theory.

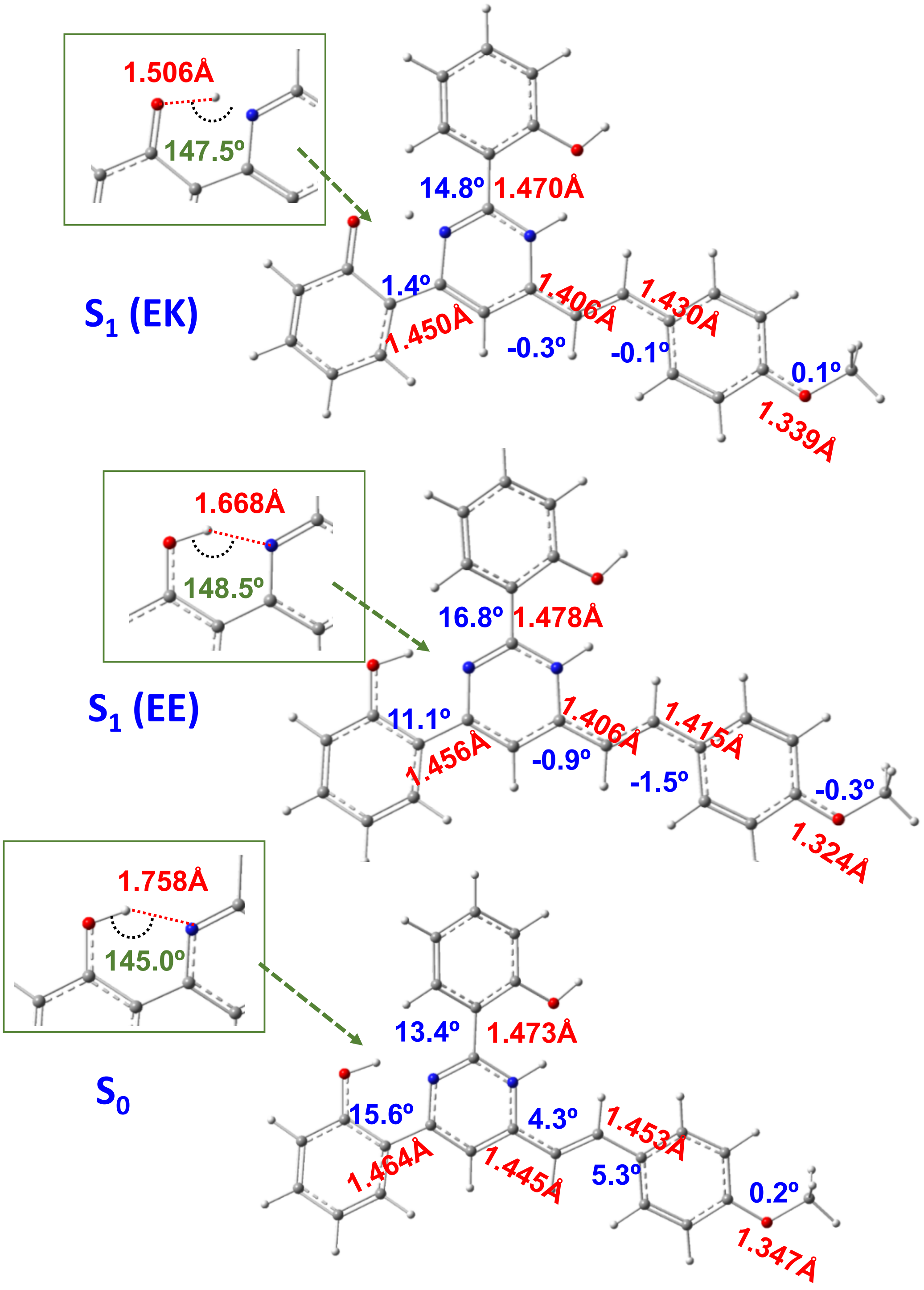

Figure S67. Selected bond lengths and dihedral angles for compound **2aH$^+$(N1)** in the S_0 and S_1 states calculated in CH_2Cl_2 at the M06-2X/6-31+G** level of theory.

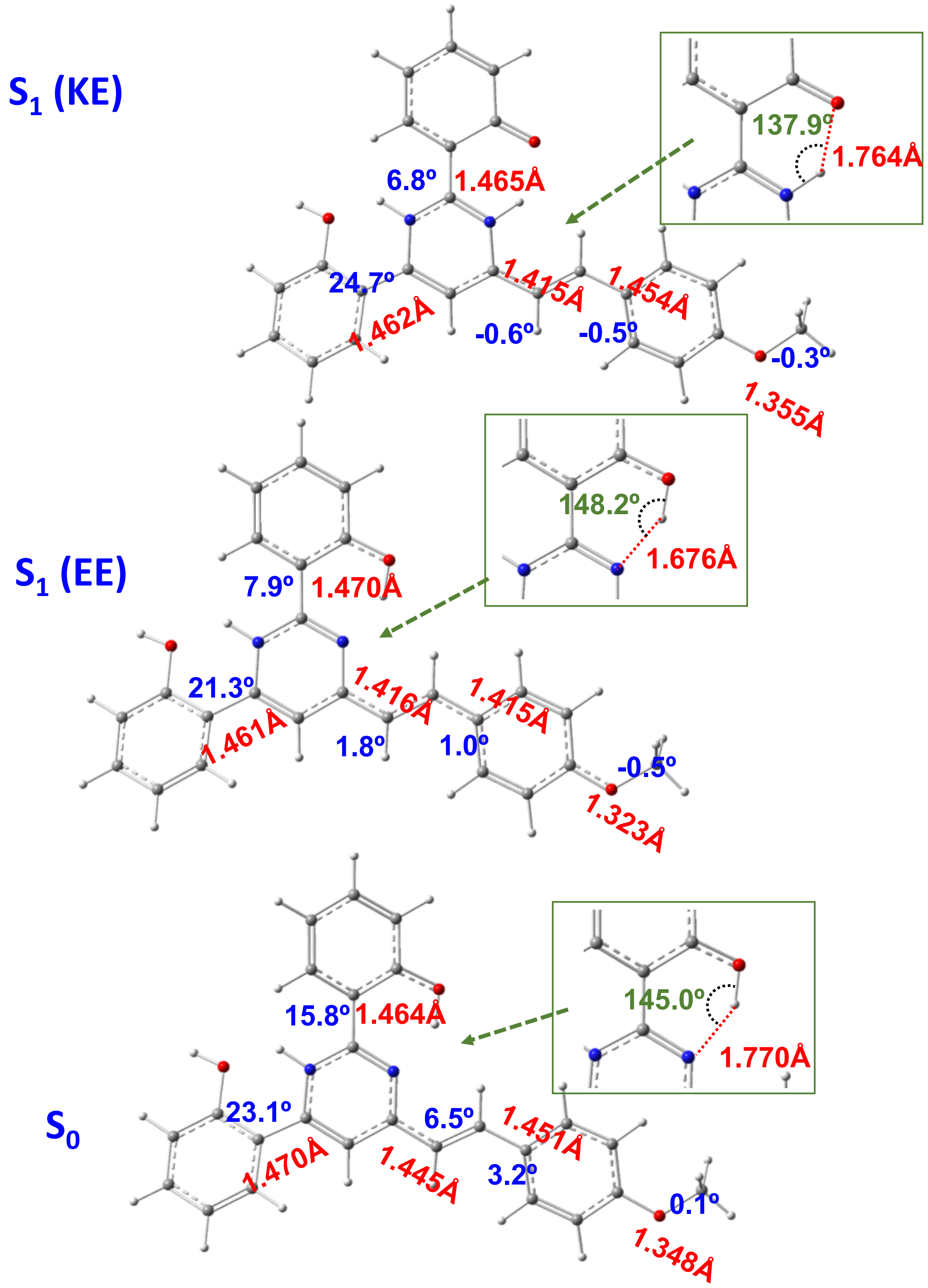

Figure S68. Selected bond lengths and dihedral angles for compound **2aH$^+$(N3)** in the S_0 and S_1 states calculated in CH_2Cl_2 at the M06-2X/6-31+G** level of theory.

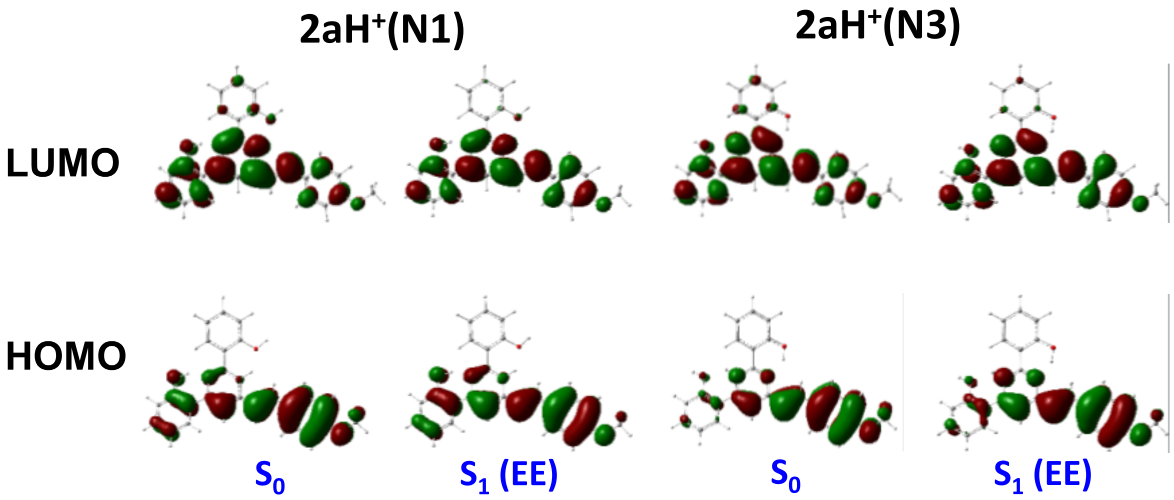

Figure S69. Molecular orbitals in CH_2Cl_2 solution calculated for **2aH$^+$(N1)** and **2aH$^+$(N3)** in the ground and excited states at the M06-2X/6-31+G** level of theory (isocontour plots 0.02 au).
